# Genome-wide gene expression and RNA half-life measurements allow predictions of regulation and metabolic behavior in *Methanosarcina acetivorans*

**DOI:** 10.1186/s12864-016-3219-8

**Published:** 2016-11-16

**Authors:** Joseph R. Peterson, ShengShee Thor, Lars Kohler, Petra R.A. Kohler, William W. Metcalf, Zaida Luthey-Schulten

**Affiliations:** 1 Department of Chemistry, University of Illinois at Urbana-Champaign, 505 S Mathews Ave, Urbana, 60801 IL USA; 2 Center for Biophysics and Computational Biology, University of Illinois at Urbana-Champaign, 1110 W Green St, Urbana, 60801 IL USA; 3 Department of Microbiology, University of Illinois at Urbana-Champaign, 601 S Goodwin AveIL, Urbana, 60801 USA; 4 Carl R. Woese Institute for Genomic Biology, University of Illinois at Urbana-Champaign, 1206 W Gregory DrIL, Urbana, 60801 USA; 5 Beckman Institute, University of Illinois at Urbana-Champaign, 405 N Mathews Ave, Urbana, 60801 IL USA

**Keywords:** Methanogens, RNA half–lives, Genome scale metabolic modeling, Degradational regulatory control, Differential pathway usage, Metabolic phenotype

## Abstract

**Background:**

While a few studies on the variations in mRNA expression and half-lives measured under different growth conditions have been used to predict patterns of regulation in bacterial organisms, the extent to which this information can also play a role in defining metabolic phenotypes has yet to be examined systematically. Here we present the first comprehensive study for a model methanogen.

**Results:**

We use expression and half-life data for the methanogen *Methanosarcina acetivorans* growing on fast- and slow-growth substrates to examine the regulation of its genes. Unlike *Escherichia coli* where only small shifts in half-lives were observed, we found that most mRNA have significantly longer half-lives for slow growth on acetate compared to fast growth on methanol or trimethylamine. Interestingly, half-life shifts are not uniform across functional classes of enzymes, suggesting the existence of a selective stabilization mechanism for mRNAs. Using the transcriptomics data we determined whether transcription or degradation rate controls the change in transcript abundance. Degradation was found to control abundance for about half of the metabolic genes underscoring its role in regulating metabolism. Genes involved in half of the metabolic reactions were found to be differentially expressed among the substrates suggesting the existence of drastically different metabolic phenotypes that extend beyond just the methanogenesis pathways. By integrating expression data with an updated metabolic model of the organism (*i*ST807) significant differences in pathway flux and production of metabolites were predicted for the three growth substrates.

**Conclusions:**

This study provides the first global picture of differential expression and half-lives for a class II methanogen, as well as provides the first evidence in a single organism that drastic genome-wide shifts in RNA half-lives can be modulated by growth substrate. We determined which genes in each metabolic pathway control the flux and classified them as regulated by transcription (e.g. transcription factor) or degradation (e.g. post-transcriptional modification). We found that more than half of genes in metabolism were controlled by degradation. Our results suggest that *M. acetivorans* employs extensive post-transcriptional regulation to optimize key metabolic steps, and more generally that degradation could play a much greater role in optimizing an organism’s metabolism than previously thought.

**Electronic supplementary material:**

The online version of this article (doi:10.1186/s12864-016-3219-8) contains supplementary material, which is available to authorized users.

## Background

The stability of an RNA molecule, as measured by its half-life, is a critical factor in defining timescales for cellular events. It also sets the correlation time of transient adaptations relative to a cell’s growth rate, when compared to its more long-term “basal” phenotype. For example, rapid, high-fidelity responses to extreme external stimuli are mediated through small RNA (sRNA, siRNA, miRNA, etc.) whose function depends largely on search time and efficiency of stimulated co-degradation, sequestration or stabilization when in complex with the target mRNA [1]. On longer timescales degradation finely tunes abundances of critical RNAs [2], controls slow shifts in RNA levels during adaptation between different growth states [3], and contributes significantly to the noise in the steady-state distribution observed in populations of cells [4]. These observations and those of many other studies demonstrate that post-transcriptional control of RNA dynamics is critical to understanding the cellular state; however, the factors defining stability over an organism’s entire transcriptome have yet to be fully defined. Furthermore, the consequences of changing RNA stability on metabolic state remains unknown.

Significant strides towards understanding the individual factors affecting RNA stability have been made. To date, genome-wide analyses of RNA stability have been reported for many single-celled, model organisms including representatives of the families *Escherichia* [5–9], *Mycobacterium* [10], *Bacillus* [11, 12], *Sulfolobus* [13–15], *Halobacterium* [15], *Methanocaldococcus* [16, 17] and various yeasts [18–21]. However, the majority of species studied were fast-growing bacterial or eukaryotic species, and archaeal species account for only a small fraction of the whole-transcriptome reports. This study aims to extend our knowledge of RNA stability in archaea by characterizing it in *Methanosarcina acetivorans*, a versatile organism capable of growth and methanogenesis using many substrates and therefore of great importance in the global carbon cycle [22, 23]. The organism has also been implicated in a historical mass extinction as the fossil record shows increase in biogenic methane along with an increase in environmental nickel, an important cofactor in the methanogenesis pathways, and the evolution of the acetotrophic (acetate utilization) pathway [24].

Genome-wide RNA stability has been characterized in the first sequenced methanogen *Methanocaldococcus jannaschii* [16, 17]; however, this organism is a class I methanogen only capable of growth wherein electrons derived from hydrogen or formate are used to reduce CO_2_ [25]. More complex class II methanogens [25] such as those in the family *Methanosarcinaceae* are capable of growing on a diverse set of substrates including mono-, di-, and tri-methylated molecules as well as acetate, carbon monoxide, and H _2_/CO_2_; thus, requiring branched methanogenesis pathways and more complex regulation to optimize their growth to a particular environment. They also generally have genome sizes 2–4 times larger than *M. jannaschii*, requiring significantly more regulators, the number of which have been found to scale quadratically in the number of genes [26].

The study of RNA stability in *M. jannaschii* identified noncatalytic cleavage sites about 12–16 nucleotides upstream of the translation start site for about a quarter of genes examined, suggesting 5 ^′^ leader sequences play a role in post-transcriptional regulation of genes [17]. Several studies posited a similar mechanism could exist in class II methanogens. One study of the operon encoding the acetyl-coenzyme-A decarbonylase/synthase complex in *Methanosarcina acetivorans* identified post-transcriptional regulation to be important in acetotrophic and carboxydotrophic methanogenesis and hints at the possibility that altering transcript stability could play a more global genetic role [27]. A very recent study in a distantly related methanogen *Methanolobus psychrophilus* has demonstrated that both transcriptional and post-transcriptional regulation play important roles providing extra stability in this slow growing, cold-adapted organism [28]. Several studies have discovered small RNAs in the related species *M. mazei* [29, 30]; however, their role in regulating transcript half-lives have yet to be established. Whether post-transcriptional regulation is widespread and whether such regulation is mediated by targeted endonucleolytic degradation or small RNA regulation or translational initiation is yet unknown. Therefore, a characterization of RNA stability in class II methanogens will help us to determine what role degradation plays in the larger context of the cell’s economy.

Regulation of gene expression by change in half-life has recently been demonstrated in *L. lactis* and *E. coli* [7–9]. The authors of these papers proposed a method to determine “control coefficients” (which describe whether mRNA abundance is transcriptionally or degradationally controlled) from half-life and expression data. They found that change in growth rate on glucose manifest small shifts in half-lives and that only about ∼10% of genes were degradationally controlled. To determine the extent to which degradation plays a role regulating gene expression in *M. acetivorans* we performed whole-genome analyses of RNA expression and half-lives in two fast growth substrates (methanol and TMA) and one slow growth substrate (acetate) and applied the control theory. We found, in contrast to the studies in *L. lactis* and *E. coli*, significant shifts in half-life with growth rate and that degradation controls gene expression for up to 28% of genes.

This study provides the first global picture of differential expression and half-lives for a class II methanogen, as well as provides the first evidence in a single organism that drastic genome-wide shifts in RNA half-lives can be modulated by growth substrate. Furthermore, we demonstrate how combining half-lives with expression data can be used to predict transcription rates and average mRNA copies per cell which can in turn be used with computational modeling to predict metabolic phenotype. In the process, we updated the most recent genome-scale metabolic model for *M. acetivorans* to include newly characterized reactions. We used expression data to constrain metabolic fluxes to generate several hypotheses about changes in the metabolic state and metabolite production due to carbon source. We created a metabolic map onto which all information generated in the study could be displayed including reaction energies, enzyme commission numbers, metabolic subsystem, cluster of orthologous group categories, differential expression statistics, half-lives, regulation control coefficients and steady-state reaction fluxes. This effectively creates a visual database which can be used to understand regulation and the associated metabolic state.

## Results

### Half-life distributions

**Fig. 1:**
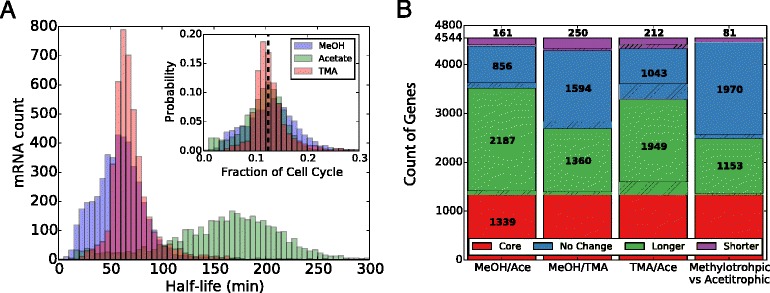
Shift in Half-Life With Growth Substrate. **a**) Genome-wide histograms of RNA half-lives for *M. acetivorans* growing in methanol (*blue*), TMA (*red*), or acetate (*green*) media. The shorter lifetimes in high-energy substrates are apparent when compared to the acetate distribution. The inset shows the distribution of half-lives after they have been scaled by doubling time (7.5hr [43, 95, 96], 8.9hr [43] and 24.6hr [43, 95, 97] for growth in MeOH, TMA and Acetate, respectively), demonstrating that the average transcript half-life is a constant fraction of the cell cycle, or about 12.7% ±3.5% the doubling time (*dashed line*). See Additional file 1: Figure S4 for a larger version of the inset. **b**) A breakdown of changes in half-life by pairwise comparison of growth conditions. Unregulated genes that show no statistically significant (t-test, *p*>0.01) shift in half-life under any of the conditions (1339 total; *red bar*) and those marked as “No Change” (*blue bar*) do not show significant changes when comparing the indicated conditions. Genes that are stabilized or destabilized when comparing the second condition to the first condition are labelled as “*longer*” (*green*) and “*shorter*” (*purple*), respectively. Hatched regions indicate the fraction of genes that are differentially expressed in addition to having different half-lives. As discussed more thoroughly in the text, almost half of the stabilized and destabilized genes are common when comparing methylotrophic conditions to acetotrophic growth, suggesting there is a similar stabilization mechanism based on either growth rate or substrate

We characterized the half-lives genome-wide for cells growing on acetate, methanol (MeOH) and trimethylamine (TMA) in order to identify changes in transcript “stability” under different growth conditions (see Fig. 1, Additional file 1: Table S1 and Figures S1 and S2). Half-lives were extracted from RNA transcriptome profiles measured at six timepoints following transcriptional arrest by the antibiotic actinomycin D as described in the methods. Transcriptome profiles at different timepoints for the same growth substrate were highly correlated (Pearson’s r >0.94, Additional file 1: Figure S3). The three biological replicates at each timepoint showed a minimum correlation coefficient of 0.99. Both of these observations indicate that our experimental procedure is reproducible (see Additional file 1: Figure S3).

Expression profiles were averaged at each timepoint and the time series normalized such that the most stable, core gene *sodB* (*MA1574*) [13] had a half-life of ∼2 hours. After normalization, most genes had positive half-lives. After fitting the degradation profile to a first-order decay function, 4486, 4487 and 3667 genes in methanol, TMA and acetate grown cultures were found to have positive half-lives with residual error in the fit below 50% of the value of the decay rate (i.e. coefficient of variation, CV <50%). Genes with negative half-life or large fitting residual were omitted in subsequent analyses. High-energy yield substrates methanol and TMA had similar average half-life—59 ± 25 min and 72 ± 29 min, respectively—while the lower-energy yield substrate acetate showed a significantly longer average half-life of 159 ± 59 min (standard deviation, *n*=3). Probability distributions of the half-lives for each growth substrate where highly statistically different (*p*<1.6×10^−133^, Mann-Whitney test) demonstrating that each substrate has unique degradation characteristics. Histograms of the RNA half-lives for the three substrates are shown in Fig. 1 a where the significant shift towards longer half-lives for growth in acetate is apparent; however, a sizable portion of the half-lives remain relatively unchanged as evidenced by the flat profile between 0 and 100 min.

In Fig. 1 b shifts in half-lives for individual transcripts comparing growth conditions and comparing methanogenesis type (methylotrophic vs. acetotrophic) are shown. A “core” set of 1339 genes showed no statistical change among any of the three conditions (t-test, *p*>0.01) which means that ∼25% of the genome is not differentially degraded; however, most other genes show some shift in half-life when comparing growth substrates. The observation that not all genes show similar shifts in half-life suggests the organism might employ a mechanism to selectively stabilize/destabilize certain mRNAs. More than 10 times as many genes were destabilized during methylotrophic growth than were stabilized, with a total of 1153 having longer half-lives in acetate than in methanol and TMA.

**Fig. 2:**
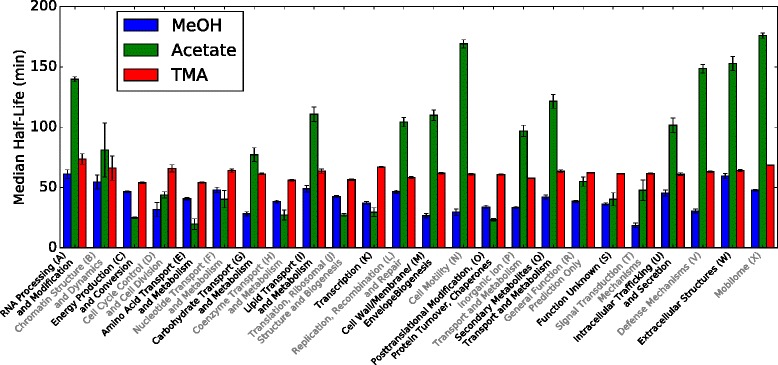
Half-Life Shift by Functional Class. The median half-lives for the 23 COG classes demonstrate different behaviors for low- and high-energy substrates. The shift in RNA half-lives between substrates are not uniform across functional classes, suggesting there exists a mechanism to selectively stabilize/destabilize the transcripts. See Additional file 1: Figure S5 for details about the median and quartiles. Uncertainties were calculated as the weighted standard deviation and are shown as error bars

To further understand the shift in half-lives seen in Fig. 1 b, genes were binned by clusters of orthologous groups (COGs, the 2014 revision [31]; arCOGs, the 2015 revision [32]). After computing statistics for the distribution of half-lives in each bin we found that the means many COG classes were significantly shifted between growth substrates (Fig. 2 and Additional file 1: Figure S5). About 11 classes of genes had significantly longer mean half-life when growing on acetate than methylotrophic conditions including RNA processing (A), carbohydrate transport and metabolism (G), lipid transport and metabolism (I), cell wall biogenesis (M), cell motility (N), inorganic ion metabolism (P), secondary metabolite metabolism (Q), intracellular trafficking and secretion (U), defence mechanisms (V), extracellular structures (W) and the mobilome (X). Additionally, three classes had significantly shorter half-lives including energy production and conversion (C), amino acid transport and metabolism (E), and translation, ribosome structure and biogenesis (J). It is interesting to note that the three classes that have shorter half-lives in acetate (slow growth) all play major roles in cell growth. Comparing MeOH and TMA growth most classes showed minor shifts except those involved in RNA processing (A), chromatin structure (B), energy production (C), lipid transport (I), replication (K) and extracellular structures (W). The selective stabilization by functional class indicates that *M. acetivorans* uses degradation to prioritize certain functions on different growth substrates.

Only 81 transcripts were stabilized in both methylotrophic conditions compared to acetotrophic growth (see Fig. 1 b). Using gene set enrichment analysis (GSEA) it was found that translation (ribosomal proteins and initiation factors) and methanogenesis (*mcr*, *cdh*, *mrp*, *hdr*) genes were highly enriched in the transcripts that were particularly stabilized during methylotrophic growth (*p*<4.9×10^−5^,*p*<2.8×10^−2^, respectively; computed via PANTHER [33]). In general, correlation of half-lives between any two growth conditions was low (|*r*|<0.15); however, half-lives for genes that were stabilized or destabilized when during methylotrophic growth had a significantly higher correlation (*r*=0.43,*p*<4×10^−53^, t-test). This observation might indicate similar stability control for growth in methanol and TMA. GSEA also indicated that transcripts involved with aromatic amino acid synthesis were especially stabilized, encoding growth rate controlled genes in Trp, His, Asp and Phe biosynthesis pathways (*p*<1.4×10^−2^; PANTHER).

We found that half-lives did not correlate significantly with RNA Gibbs energy of folding of the ORF as computed using state-of-the-art RNA folding software (*r*=0.018,*p*=0.23 by t-test, for both Andronescu2007 and Turner2004 parameters; data not shown). This confirms previous reports which found no correlation of the folding energy to RNA half-life [5, 11]. RNA stability was also not correlated with gene length (|*r*|<0.09; data not shown). Similarly, changes in RNA stability between growth conditions were not correlated to RNA folding energy or gene length, suggesting different attributes determine stability; perhaps the number of internal cleavage sites, 5 ^′^ or 3 ^′^ untranslated regions, susceptibility to different RNases or regulation by sRNAs.

### Differentially expressed genes

The regulation of gene expression may be a functional role for the selective stabilization of mRNA transcripts. To confirm this hypothesis, we needed to determine which genes were regulated. A comparison of gene expression for cultures growing exponentially in the three media was performed. Three methods [34–36], each employing different underlying assumptions about gene expression, were used to predict statistically differentially expressed genes (DEG). Because each method gave diverse results, we took the common set of DEG—hereafter referred to as the “consensus set”—to be a conservative estimate of the DEG (see Additional file 1: Figure S7). Our observed fold change in RNA expression between acetate, MeOH and TMA are in good agreement with the previous, but limited, qRT-PCR and microarray studies that have been published [37] with a Pearson correlation coefficient, r, of 0.82 (*p*<3×10^−9^; Additional file 1: Figure S6A). We also found that, while absolute protein count was very weakly correlated to RNA expression (*r*=0.12,*p*>0.1), fold change in protein levels [38–43] were highly correlated to change in RNA expression (*r*=0.63,*p*<2.2×10^−11^; Additional file 1: Figure S6B).

**Fig. 3:**
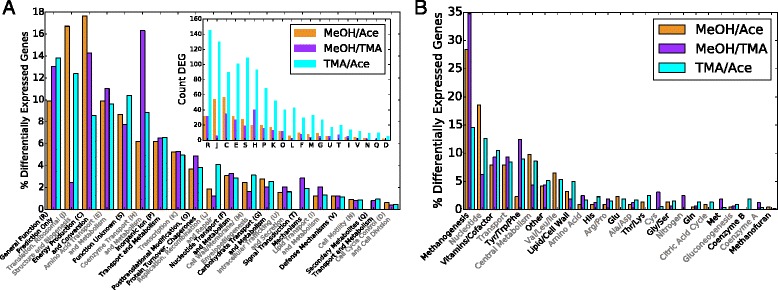
Breakdown of Differentially Expressed Genes. Breakdown of differentially expressed genes (DEG) comparing MeOH/Acetate (*orange*), MeOH/TMA (*purple*) and TMA/Acetate (*cyan*) by (**a**) COG class and (**b**) metabolic subsystem (metabolic genes include those that are associated with reactions in the metabolic model *i*ST807). The outliers in (**a**) include coenzyme/vitamin metabolism (H) and translation and ribosome biogenesis (J) when comparing MeOH and TMA. The inset in a shows the count of DEG in each category. The inset in (**a**) shows the count of DEG in each category. Genes with a *p-value* ≤ 0.01 were considered to be differentially expressed

Counts of differentially expressed genes can be found in Table 1. The consensus set consisted of 201 ±50 DEG comparing methanol and acetate (MA), 645 ±162 DEG comparing TMA and acetate (TA) and 211 ±62 DEG comparing methanol and TMA (MT) members with a p-value < 0.01 (see Additional file 1: Figure S7 for overlap between methods). The uncertainty in number of differentially expressed genes due to limited replicates was estimated to be 24–30% using a bootstrapping procedure (see Additional file 1: Section “**Uncertainty in Differentially Expressed Genes**” and Figure S2). Genes involved in energy metabolism (C), translation (J), coenzyme metabolism (H), and amino acid metabolism (E) are most drastically effected, though all COG classes have at least one representative gene (Fig. 3 a). We also computed differential expression for gene operons. To do so, we combined reads over gene operons (as predicted by four different methods [44–47]) and applied the same methods as for genes. We found that the consensus set DEG computed by operons largely reflected those computed by individual genes and that about 80% of genes were called as differentially expressed (see Table 1).

**Table 1:** Count of genes that are differentially expressed when comparing growth substrates predicted by several methods

Genes					
Comparison	edgeR	DESeq2	PoissonSeq	Consensus^b^	
MeOH vs Acetate	621	341	400	201 (126^d^)	
MeOH vs TMA	2839	258	2348	211 (112^d^)	
TMA vs Acetate	2762	757	1955	645 (335^d^)	
Methylotrophic vs	511	179	301	100	
Acetotrophic					
Operons^a^					
Comparison	DOOR2	Microbes Online	ProOpDB	Rockhopper	Consensus^c^
MeOH vs Acetate	205 (144)	183 (110)	189 (129)	207 (151)	157
MeOH vs TMA	202 (148)	198 (117)	211 (143)	212 (158)	163
TMA vs Acetate	662 (450)	701 (343)	667 (406)	655 (469)	571
Methylotrophic vs	112 (75)	97 (53)	91 (56)	112 (79)	76
Acetotrophic					

^a^Intersection of the sets of differentially expressed genes predicted by the three methods. ^b^Count of differentially expressed genes that are associated with reactions in the metabolic model. ^c^The differential expression procedure applied to reads summed over putative operons of a specific dataset, where the number reported is total genes called as differentially expressed, while the number in parenthesis is the total number of operons called as differentially expressed. ^d^Intersection of the sets of differentially expressed genes predicted to be in differentially expressed operons (because operon structures were not conserved across the methods)

**Fig. 4:**
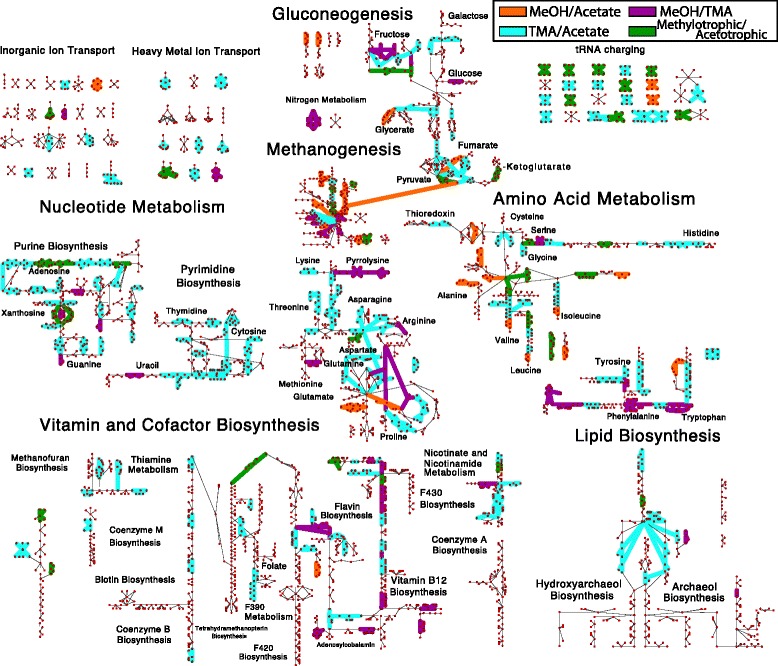
Mapping of Differentially Expressed Genes (DEG) on Metabolism. The map of all known metabolic reactions effected by differentially expressed genes comparing MeOH vs. acetate (*orange*), MeOH vs. TMA (*purple*), TMA vs. acetate (*cyan*) and MeOH/TMA vs. acetate (*green*). Reactions and metabolites are represented as *green diamonds* and *red circles*, respectively. Reactions are connected to participating metabolites by edges. Common metabolites are duplicated. Unregulated reactions are indicated by *thin lines*. Genes were considered differentially expressed if the *p*-value ≤ 0.01 as computed in all of the three methods: DESeq2, edgeR and PoissonSeq. Reaction and metabolite names can be seen by zooming into Additional file 1: Figures S19 and S20

Differentially expressed genes were largely associated with metabolic reactions. Of the 201, 211 and 645 differentially expressed genes identified, about half were associated with reactions in our genome-scale metabolic models for *M. acetivorans* [48, 49] (affecting 149, 110, 359 total reactions, respectively). Genes associated with energy metabolism account for only 8–18% of all DEG depending on compared substrates (Fig. 3 a), demonstrating that carbon source plays a larger role in defining metabolic state than merely by changing expression of methanogenesis genes (Fig. 3 b). Nucleotide, cofactor, aromatic amino-acid biosynthesis and transport metabolic pathways were also regulated extensively, each accounting for between 5 and 15% to total regulated genes (Fig. 3 b). Notably, genes in translation also contribute to ∼3–16% of all differentially expressed genes, suggesting a tight coupling between growth substrate and genes affecting growth rate (Fig. 3 a). In metabolism methanogenesis accounts for 15–35% of differentially expressed genes alone; however, genes involved in nucleotide, vitamin and cofactor biosynthesis as well as transport each constitute ∼10% of differentially expressed genes, suggesting that growth substrate has significant effect on the cell economy, likely affecting energy carrier balance and import and export of nutrients. Figure 4 shows a mapping of differentially expressed genes.

### Regulatory control coefficients

**Fig. 5:**
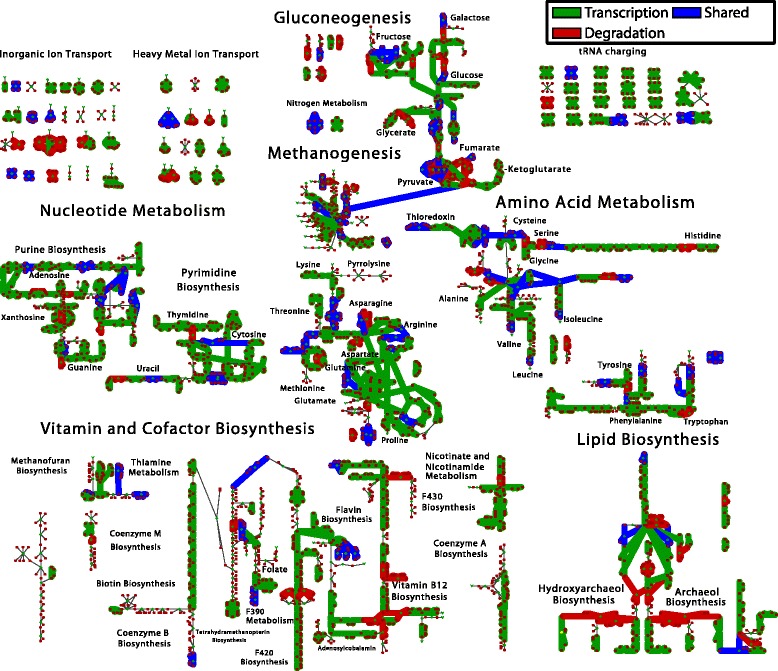
Control Coefficients Mapped onto Metabolism. A mapping of the control coefficients for changing mRNA expression levels between TMA and acetate. *Red* indicate reactions where mRNA levels are regulated by shifts in the degradation rate, while *green* indicates mRNA level shifts due to changes in transcription rate. *Blue* indicates reactions where mRNA levels are affected by both transcription and degradation rate. Reaction and metabolite names can be seen by zooming into Additional file 1: Figures S19 and S20

In light of the fact that half-lives for mRNAs change significantly between growth substrates and that the changes are specific to certain functional classes of mRNA, it is likely that selective degradation of mRNAs plays a regulatory role. A recent theory was proposed to determine whether change in transcript abundance for a gene between two growth conditions is determined by change in the degradation or transcription rate [7, 8]. The theory defined “control coefficients” that describe the effective change in mRNA level as resulting from degradation or transcription, under the assumption that gene expression is at steady-state (i.e. the population is growing exponentially and in homogenous growth condition). We computed the degradational (*ρ*_*D*_=−*dln* *γ*/*dln*[*mRNA*]; where *γ* is the degradation rate) and transcriptional (*ρ*_*T*_=*dlnk*_*trn*_/*dln*[*mRNA*]; where *k*_*trn*_ is the transcription rate) control coefficients for each of the genes using the half-life and expression data. See Additional file 1: Section “**Control Coefficients**” for a derivation and description of these control coefficients. See Fig. 5 for a mapping of control coefficients comparing TMA and acetate.

Three regulation regimes are of interest [7–9]: 1) primarily degradationally controlled, (*ρ*_*D*_≥1), 2) primarily transcriptionally controlled, (*ρ*_*D*_≤0) and 3) mixed degradation and transcription control 0<*ρ*_*D*_<1. The results for all genes can be found in Table 2. Our analyses show that between 16 and 28% of the changes in steady-state transcript levels are due to degradational control. Between 16 and 23% of transcripts show both degradational and transcriptional control effects. A summary of the control coefficients computed for differentially expressed genes can be seen in Table 3. Strikingly, more than 50% of all differentially expressed metabolic genes, defined as the 807 genes which are associated with reactions in the metabolic model (*i*ST807; see the next section), were primarily controlled by degradation. This high percentage underscores the substantial role that degradation plays in regulating metabolism in *M. acetivorans*.

**Table 2:** Classification of regulation type between growth conditions for all genes

	Transcriptionally controlled	Shared control	Degradationally controlled
	*ρ*_*D*_≤0	0 <*ρ*_*D*_<1	*ρ*_*D*_≥1
Comparison	Number (%^a^)	Number (%)	Number (%)
MeOH vs. TMA	2987 (67.2)	718 (16.1)	742 (16.7)
MeOH vs. Ace	2219 (61.2)	562 (15.5)	847 (23.3)
TMA vs. Ace	1763 (48.5)	844 (23.3)	1026 (28.2)

^a^Percentage of total genes for which half-lives and RNA reads were of sufficient quality to apply the control analysis (i.e. *CV*_*τ*_<0.5)

**Table 3:** Classification of regulation type for differentially expressed metabolic genes in the consensus set from Table 1. Metabolic genes are defined as those that are associated with a reaction in the metabolic model *i*ST807

	Transcriptionally controlled	Shared control	Degradationally controlled	Indeterminate^b^
	*ρ*_*D*_≤0	0 <*ρ*_*D*_<1	*ρ*_*D*_≥1	
Comparison	Number (%^a^)	Number (%^a^)	Number (%^a^)	Number (%^a^)
sectionMeOH vs. TMA	54 (25.6)	23 (10.9)	126 (59.7)	8 (3.8)
MeOH vs. Ace	25 (12.4)	49 (24.4)	97 (48.3)	30 (14.9)
TMA vs. Ace	107 (16.6)	149 (23.1)	320 (49.6)	69 (10.7)

^a^Percentage of DEG out of those for which half-lives and RNA reads were of sufficient quality to apply the control analysis (i.e. CV <0.5). ^b^Regulation coefficients could not be determined because half-life data was low quality (CV ≥0.5)

### Metabolic model for *M. acetivorans*

Modifications to the metabolic reconstruction and model for *M. acetivorans* were necessary in order to simulate the effect of regulation on pathway usage. We updated the genome-scale metabolic reconstruction *i*MB745 [48] by incorporating newly characterized pathways and gene:reaction mappings. Several additional model improvements dealt with amino acid synthesis and ligation (e.g. tRNA-charging). First, the pathway for pyrrolysine biosynthesis was added to reflect the requirement for this amino acid for cells growing on methylamine substrates (Additional file 1: Figure S8). Second, the alternate cysteine aminoacylation pathway, wherein *O*-phosphoserine is converted to cysteine while charged to tRNA ^*Cys*^, was added [50, 51] (Additional file 1: Figures S9 and S10). Third, tRNA-charging was explicitly included in the model, wherein the protein biomass composition was altered to require charged tRNAs instead of free amino acids. This change allows comparison of differential expression of tRNA genes with metabolic flux. Fourth, the biosynthesis pathway for methanofuran was updated based on recent characterization of the five *mfn* genes [52–55]. Methanofuran is a component in the methyl-oxidation branch of methanogenesis playing a role in a key redox step inter-converting a formyl group and CO_2_ and is required for all modes of methanogenic growth [37, 56]. Fifth, metabolic pathways for incorporating the methylated sulfur compound, methylmercaptopropionate, have been added/updated based on recent molecular biology studies [57]. Finally, several genes and a recently characterized reaction have been added to lipid biosythesis [58–60].

The model biomass equation was refined by incorporating osmolytes, salts, and metals [61] and gluconeogenesis intermediates [62]. Additionally, pyrrolysine was added as a requirement for growth on methylamines, allowing for the correct prediction of *pyl* gene knockouts when growing on these substrates (Additional file 1: Figure S11). The model was modified to allow uptake of cysteine, a component of the media [63], at a rate consistent with experiments. Newly required osmolytes *α*-glutamate, *N*-acetyl- *β*-lysine and glycine betaine fix several dead-end pathways in the model, thus increasing the predictive capability of the model (see “Discussion” section). Incorporating ion and metal requirements allows the model to predict how differential expression of membrane bound transporters affect osmolyte concentrations. Overall, the model can now take up all of the components of the Wolfe media [63] for which metabolites exist in the model (see Additional file 1: Table S2).

Altogether the new metabolic reconstruction consists of 759 non-biomass reactions (829 when including metabolite exchange) with 807 associated genes. The reconstruction was laid out as a map to allow visualization of metabolic fluxes and gene expression data (see Fig. 4 and Additional file 1: Figure S12). The map is available in formats compatible with the Cytoscape [64] and Escher [65] software. The map and modified FBA model (called *i*ST807) are available in several formats in the additional files accompanying this manuscript. See Additional file 1: Section ‘**Modifications to Metabolic Model**” for a complete discussion of map and model modifications and verification (see Additional file 2 for model and maps).

## Discussion

### Regulation of half-lives

We found that *M. acetivorans* growing on different substrates exhibits drastically different RNA half-life stabilities. A much stronger growth rate effect was observed than in the previous studies; whereas half-lives in *E. coli* were shifted by a factor of 1.5 for a 6 fold change in growth rate, in *M. acetivorans* a nearly linear shift in half-lives with growth rate was observed. To test the hypothesis that half-life is correlated to growth rate, we scaled the half-life distributions by the doubling time, effectively defining a fraction of the cell cycle that an RNA persists before degradation (see Fig. 1 and Additional file 1: Figure S4). As demonstrated, the scaled half-life distributions align with means that are statistically indistinguishable (*p*>0.33, t-test). This scaling indicates that for a given growth substrate, the cell will modulate mRNA stability such that the half-lives are, on average, a constant fraction of the cell cycle. We could not identify any differentially expressed RNases among our data which would facilitate these changes in half-lives indicating that another mechanism is in play (perhaps sRNA, riboswitches, etc.).

**Fig. 6:**
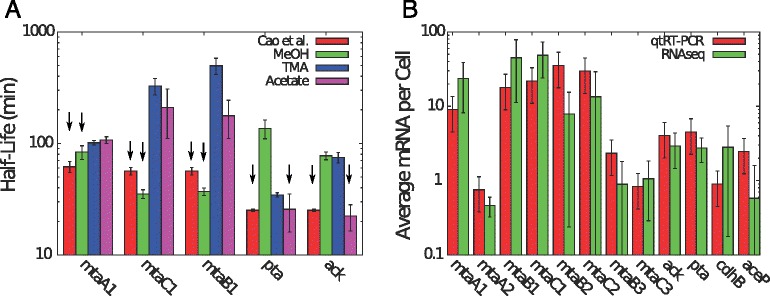
Comparison of Transcripts with *Methanosarcina mazei* [66] **a**) A comparison of mRNA half lives measured via our RNAseq data compared with a previous study using qtRT-PCR in the related organism *Methanosarcina mazei* growing in methanol or acetate. Cao et al. measured methyltransferase (*mtaA1*, *mtaCB1*) half-lives from methanol grown cells, while they measured acetoclastic gene (*pta*, *ack*) half-lives for acetate grown cells. As can be seen in the figure half lives match for methanol and acetate grown cells. Arrows indicate which bars correspond to the comparison to Cao et al. **b**) A comparison of mRNA copies per cell estimated via our RNAseq data, and previous studies that utilized RT-qPCR to quantify transcript abundance in the related organism *Methanosarcina mazei* grown in methanol (see Additional file 1: Figure S13 for acetate growth). Error bars are standard deviation of the mean for 3 replicates. Values from Cao et al. are for cells grown at 30 °C compared to our cells which were grown at 37 °C. All values agree within uncertanties except for *cdh*, *mtaA2*, and *mtaB2* indicating the organisms have similar expression profiles and our estimates for mRNA counts are good

Studies have examined how conserved mRNA half-lives are among related species. One study on two strains of *Bacillus cereus* showed high correlation among half-lives (*r*=0.72) [12] and another compared two species of the *Solfolobus* genus also finding high correlation (*r*=0.51) [14]. These studies show that RNA degradation is evolutionarily conserved and suggest that our study of RNA stability in *M. acetivorans* may be extended to related organisms such as *M. mazei* or *M. barkeri*. We compared our measured half-lives to five that had been previously measured in *M. mazei* [66] and found them to have similar values (Fig. 6 a). We also estimated mRNA copies per cell for 12 transcripts in methanol and acetate growth conditions (Fig. 6 b and Additional file 1: Figure S13 and Additional file 3). Transcript counts also matched those measured in *M. mazei* [66] suggesting these *Methanosarcina* species could have similar transcription and degradation characteristics, similarly to the two *Sulfolobus* species. In general some of the conclusions drawn from this study might hold for evolutionarily related methanogens (e.g. class II methanogens). However, half-lives of homologous genes are not correlated between distantly related organisms such as *E. coli* and *B. subtilis* [11], and therefore care should be used when extending the conclusions about individual transcripts here to distantly related methanogens (e.g. class I methanogens).

### Inheritance of gene regulation

Our genome-wide study identified many DEG in addition to those previously identified due to the higher number of experimental replicates (higher statistical power of differential expression test) and the greater number of compared growth conditions. The current study verified 80% of previously [37] identified DEG in *M. acetivorans*, indicating that the RNA data is of sufficient quality to match potentially higher accuracy methods such as qtRT-PCR. Additionally, our pattern of DEG comparing methanol and TMA was similar to the one reported for *M. mazei* [67]. A total of 42 of the 71 directly homologous genes had similar patterns of differential expression in our dataset were highly correlation in fold change (*r*=0.85,*p*<10^−5^). The similarity of transcript abundance and half-life and similarity between differentially expressed genes (see Fig. 6) suggest that regulation is conserved among these closely related organisms. Genes that are similarly differentially expressed between these two *Methanosarcina* species include methyltransferases for methanol and methylamine assimilation, a putative thiamine biosynthesis gene (*thiC*), genes involved in valine, leucine and aromatic amino acid biosynthesis (*aroDE*, *leuA*, and *trpABE*), cobalt metabolism enzymes (*MA1418* and *MA3250*) as well as many hypothetical proteins and regulators. The rest of the genes either had no homologs or were not determined to be differentially expressed. Genes that were not identified as differentially expressed could be optimized for the different environments in which *M. mazei* and *M. acetivorans* grow; perhaps due to the adaptation to freshwater and saline environments. A recent study of *M. mazei* strains along the Columbia River show differences in genomic content comparing those in fresh- and salt-water environments that resulted in differences in metabolic efficiency/utilization of TMA [68].

Genes coding for enzymes involved in biotin synthesis, including biotin synthase (*bioB*), were found to be very highly expressed (>4×) when growing on TMA compared to the other substrates. This along with the observation that growth on TMA of the closely related methanogen *Methanohalophilus mahii* was stimulated by addition of biotin suggesting that it plays a role in methylamine-based growth [69], perhaps as a cofactor involved in vitamin and lipid biosynthesis.

### Identification of regulated transcription factors

Over 200 putative transcription factors have been annotated in the DBD transcription factor database [70]. We found that 10, 9 and 13 of these transcription factors were significantly differentially expressed upon comparing MeOH vs acetate, MeOH vs TMA and TMA vs acetate, respectively. A number of the regulators have been characterized and are of particular interest. For example, the gene *msrA* (methanol-specific regulator A) was found to be highly expressed in both methylotrophic growth substrates confirming a previous report [41]. Additionally, *msrC* and *msrF* were found to be more highly expressed in TMA than acetate, also confirming previous experiments [41, 71].

For the uncharacterized transcription factors we examined their expression characteristics to attempt to identify regulatory roles and targets (see Additional file 1: Figure S14 for correlations). Two putative transcription regulators (*MA2055* and *MA3302*) were more highly expressed in acetotrophic growth, the latter of which has previously been suggested to be a global regulator of methanogenesis pathways and dubbed *mreA* (*Methanosarcina* regulator of energy-converting metabolism A) [72]. The former of these is an uncharacterized MarR-like protein that has a similar gene expression profile to another transcription regulator *MA2212*, being correlated with genes involved involved in acetotrophic methanogenesis and ATP production (Additional file 1: Section Figure S14; “**Differentially expressed genes**”). Two other *mre* like regulators were found to be differentially expressed: 1) *mreB (MA1671)*, when comparing TMA and acetate (and almost significant for TMA vs MeOH, *p*=0.0103), and 2) *mreD (MA3130)*, which was found to be more highly expressed in methylotrophic conditions and sits in a conserved cluster of genes that also contains *hdrABC*. The hdrABC homologs are differentially expressed under different growth conditions as previously reported [43]. These genes reroute metabolic flux allowing these microbes to outcompete other organisms [43, 73]. The proximity of *mreD* to *hdr* in the genome suggests it could regulate *hdrABC* differentiating methyltotrophic and acetotrophic growth, while *mreB* could differentiate methylamine growth from other conditions, potentially in optimizing one/several of the other *hdr* homologs; however, these hypotheses remain to be tested.

The putative nickel response regulator *MA1395* is highly conserved among all the methanogens and is anti-correlated to Hsp60 genes (*MA0086*/*MA1682*/*MA4413*) along with several key metabolic genes such as pyruvate synthase (*por*, *MA0031*–*MA0034*) and methenyltetrahydromethanopterin-cyclohydrolase (*mch*, *MA1710*), suggesting that during nickel starvation key metabolic enzymes are downregulated during methylotrophic conditions (see Additional file 1: Figure S14). It is, however correlated with quinolinate synthase genes (*MA0959*/*MA2716*), suggesting when nickel is taken up, more NADH/NADPH should be produced, and phoshoglycerate dehydrogenase (*MA0592*), which produces a precursor in the pathway that produces coenzymes F420 and F390. If *MA1395* does indeed regulate these genes it could act to sense available nickel in the environment and slow metabolism (via *mch* and *por*) while affecting redox balance (via production of NADH/NADPH and coenzyme F420). A previous study on regulation in *Methanococcus maripaludis* identified a homolog of *MA1395* (*MMP0719*) as being coexpressed with *mch* along with the methyltransferase *mtr*, the energy conserving hydrogenase *ehb* and genes involved in pyrophosphate uptake (*ppaC*) and purine biosynthesis (*purP*) [74]. Together these suggest an ancient role for *MA1395* that needs to be further studied.

### Regulation of general transcription factors

Our data is consistent with a previous study showing that the primary TATA binding protein (TBP; *tbp1*) transcript was similarly expressed under the three growth conditions [75]; however, it differs for comparisons of accessory TBPs wherein our data suggest that *tbp2* and *tbp3* are expressed at similar levels to each other, while the previous report showed that the latter of the two was much less highly expressed. Both [75] and our study show that *tbp3* is more highly expressed during methylotrophic than acetotrophic growth (almost identical fold changes in both studies), and this is supported by the observation that genes in amino acid metabolism and methylamine metabolism are differentially expressed upon its knockout as seen previously. Discussion of four additional transcription factors can be found in Additional file 1: Section “**Differentially expressed genes**”.

### Regulation of translation machinery

During methylotrophic growth, proline, lysine and arginine tRNAs are more highly expressed as seen in the “tRNA charging” reactions in Fig. 4. Additionally, valine, alanine and methionine tRNAs are more highly expressed under methanol growth than acetate growth, and threonine more highly during methylamine growth (see Fig. 4). Generally, they are 3–42 × more highly expressed in methylotrophic conditions, suggesting either: 1) there is a much higher requirement for these amino acids under methylotrophic growth, or 2) the slower growth in acetate can tolerate lower amounts of tRNAs, potentially due to the longer time allowed to find ribosomes while maintaining a protein production rate necessary for steady growth. Similarly, the genes coding ribosomal proteins are a factor of 8 times more highly expressed in methylotrophic growth conditions. These results lend additional support to the idea that cells differentially regulate ribosome numbers which have been shown in bacteria (*E. coli*; 6,800–72,000 depending on growth rate [76]) and archaea (*Haloferax volcanii*; 11,600–25,400 depending on growth rate [77]). This constitutes the first detailed analysis of differential expression of translation machinery in *M. acetivorans*.

### Regulation of vitamin and cofactor metabolism

Vitamin and cofactor biosynthetic pathways include many differentially expressed genes (see Fig. 4), suggesting they play important roles in each of the growth conditions. Six genes involved in nicotinamide (*nadA1*), coenzyme F420 biosynthesis (*cofH1*, *mptA*), and cobalamin biosynthesis (*cbiX*) are more highly expressed during methylotrophic growth. These enzymes are found at the beginning of a linear pathways or at the branch-point of two pathways, allowing their regulation to have a large impact on production of these cofactors. Additionally, 13 genes are most highly expressed in methylamine metabolism, including many in the pathway forming adenosyl-cobyric acid (*cbiCFHL*) and the final step thereof (*cobQ*), and those in heme production (*hemC*), riboflavin biosynthesis (*ribH*) and anthranilate synthase (*trpGE*). The gene involved in riboflavin biosynthesis is at a branchpoint of the coenzyme F420 biosynthesis and cobalamin biosynthesis, and thus has the potential to divert metabolic flux, in the case of growth on methylamines, towards production of cobalamin, consistent with the fact that cobalamin biosynthesis pathway transcripts are highly expressed. We hypothesize that larger amounts of adenosylcobalamin are required for growth in the methylamines with one possible explanation being that there are three different methyltransferase systems encoded by *mtmCBA*, *mtbCBA* and *mtmCBA* which process tri-, di- and monomethylamine, respectively, abstracting a single methyl group each. If enzymatic activity does not vary significantly between the three enzymes, and therefore a similar amount of each protein exists in a cell to maintain a certain metabolic flux, three times the equivalents of cobalamin would be needed compared with growth on methanol. The differential expression data indicates that enzymes involved in cobalamin synthesis are in fact 2.5-3.5x more highly expressed in trimethylamine growth than in methanol growth, supporting this hypothesis. Biochemical characterization could be used to test this hypothesis.

### Control of gene expression by transcription or degradation

Many DEG in the consensus set were represented in gene-protein-reaction relations as part of *i*ST807, suggesting that the regulation due to different growth substrates could have large effects on the distribution of metabolic fluxes. A composite showing reactions affected by differentially expressed genes demonstrates significant regulation throughout metabolism (Fig. 4). Key control points in linear pathways stand out, and we observe that regulation is generally clustered around branches in pathways (for example at the branchpoint between flavin biosynthesis and coenzyme F420 biosynthesis and extensively on the pathways leading from pyruvate towards different amino acids). Within the set of DEG, two obvious classes arise: methylotrophic and acetotrophic growth (contributing to 75 and 10% of the total variance computed via PCA; Additional file 1: Figure S1) which are classes with which to identify differential pathway usage.

Because the total concentration of transcripts associated with a gene are affected by both transcriptional rate and degradational rate the question of which factor plays the largest role is of interest. Dressaire et al. [8] recently proposed a method to determine whether the level of a transcript is primarily controlled by degradation, transcription, or both and applied it to *L. lactis* and found that degradation played a role in setting transcript level for maximally 12% of genes studied. The method was subsequently applied to *E. coli* by Esquerré et al. [7] showing a similarly small effect. In the latter case, a role of degradational control was found to play an important role in glycolysis, pentose phosphate, Entner-Doudoroff pathways and the TCA cycle. Furthermore, they identified a role of degradational control in setting the levels of key degradational machinery transcripts including several RNases and Hfq. Both of these studies, however, used chemostat experiments for cultures growing in one growth substrate, and the question remains whether degradation plays a larger role in optimizing an organism for different growth substrates. We applied the analysis to generate the transcriptional (*ρ*_*T*_=*dlnk*_*trn*_/*dln*[*mRNA*]) and degradational (*ρ*_*D*_=*dln* *γ*/*dln*[*mRNA*]) control coefficients, which describe the relative change in mRNA due to relative changes in transcription rate *k*_*trn*_ and degradation rate *γ* (see Additional file 1: Section “**Control Coefficients**”). In contrast to the previous single substrate experiments in *L. lactis* and *E. coli*, our analyses show that between 16 and 28% of the changes in steady-state transcript levels are due to degradational control (Table 2). A close analysis of the data leads to the striking conclusion that degradational control appears primarily at branchpoints and is enriched in amino acid metabolism, lipid metabolism and vitamin metabolism (Figs. 5 and 10 a, c, and e). This localization at pathway branchpoins could indicate an important uncharacterized role for degradational control. And because more than half of differentially expressed metabolic genes appear to be controlled by change in degradation rate it is likely that the change in degradation plays a significant role regulating metabolism in *M. acetivorans* (see Table 3). If a regulated gene is significantly destabilized (stabilized), the production of its protein is expected to proportionally decrease (increase). This is because the average protein count for a gene should go as $\langle P\rangle \propto k_{t} k_{r} \tau /k_{dil}$ where *k*_*t*_ is the transcription rate, *k*_*r*_ is the translation rate, *τ* is the half-life and *k*_*dil*_ is the doubling rate. This argument neglects translational regulation with growth condition, which has been shown to exist in Eukaryotes [78] and in haloarchaea up to 30% of all transcripts [79, 80]. Translational regulation will additionally effect the *k*_*r*_ in this equation, potentially causing a nonlinear response when coupled with the change in *k*_*t*_ or *k*_*d*_.

### Modelling metabolic phenotype

**Fig. 7:**
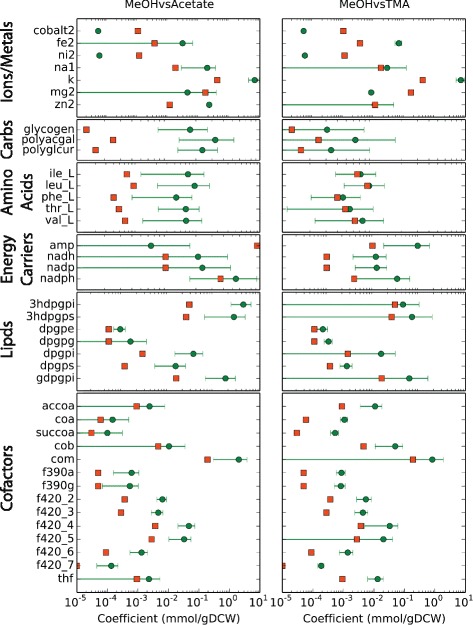
Fitted Biomass Coefficients. A comparison of fitted biomass coefficients. *Orange squares* indicate the coefficients for growth in MeOH while the *green circles* indicate the optimized biomass coefficients. *Large error bars* indicate that the coefficient can take on many values while still being optimal. Only metabolites with a significant shift comparing either MeOH to acetate or MeOH to TMA are included in the plot (all fitted biomass coefficients can be found in Additional file 1: Figure S15)

**Fig. 8:**
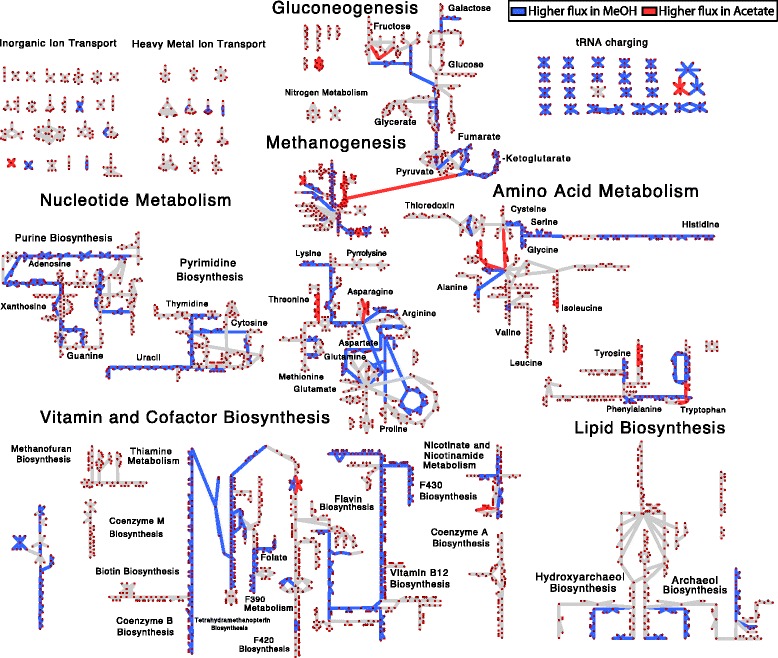
Metabolic Flux Differentces between MeOH and Acetate. Predicted changes in flux comparing growth on methanol to growth on acetate. Pathways that carry more flux (>2 fold change in flux) when grown on acetate are indicated by *red* while those that carry more flux when grown on methanol are indicated by *blue*. Unaffected pathways are shown as *grey lines*. Reaction and metabolite names can be seen by zooming into Additional file 1: Figures S19 and S20

**Fig. 9:**
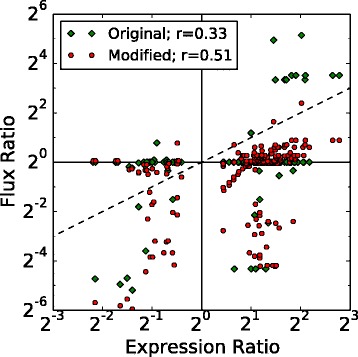
Metabolic Flux vs Gene Expression. Ratio of metabolic fluxes compared to ratio of gene expression for growth in different media. Each point represents a mapping between one reaction and one gene; therefore each reaction or gene may be represented by multiple points. If the same biomass requirements are used for the different growth substrates few of the reactions show any difference in flux (*green diamonds*) and there is weak correlation between expression and flux. The differences in fluxes that are observed are primarily due to genes encoding proteins that act in methanogenesis. By relaxing the assumption that biomass coefficient are constants across all growth substrates the model can be fit to improve the correlation between regulation and metabolism (*red circles*). After fitting, many additional pathways are predicted to carry different flux, as demonstrated by more points moving off the horizontal towards *y*=*x* (*dashed line*)

**Fig. 10:**
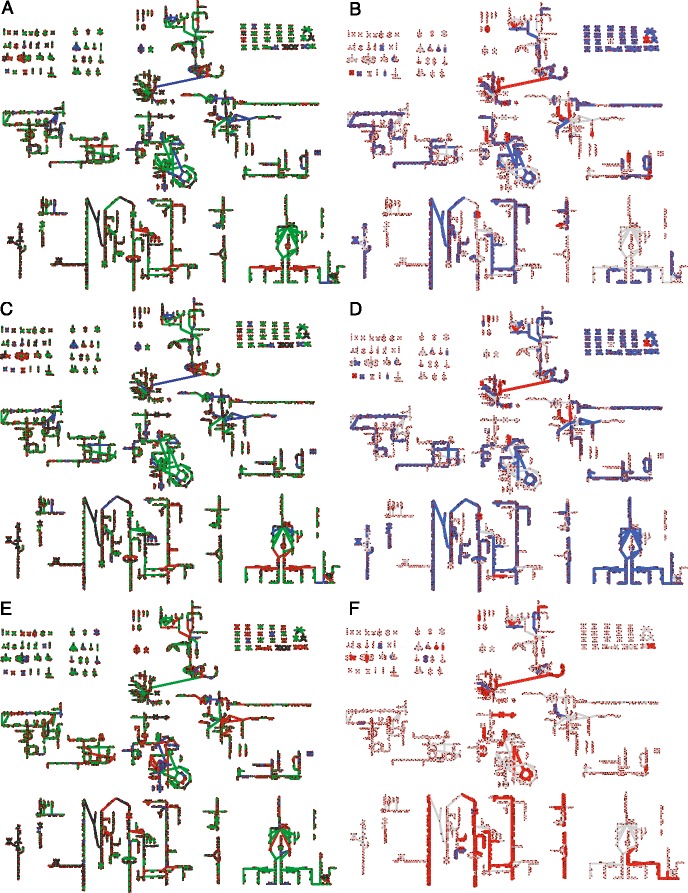
Control Coefficients and Fluxes contrasting all Substrates. Comparisons of control coefficients (**a**, **c**, **e**) to predicted metabolic fluxes (**b**, **d**, **f**). (**a**, **b**) MeOH vs Acetate, (**c**, **d**) TMA vs Acetate, (**e**, **f**) MeOH vs TMA. Control coefficients (**a**, **c**, **e**) indicate that reactions are transcriptionally controlled (*green*), degradationally controlled (*red*) or shared control (*blue*). Mappings of predicted metabolic fluxes indicate higher flux in the second substrate (*red*) versus lower flux in the second substrate (*blue*). Larger versions of (**a**, **b**) can be seen in Figs. 5 and 8. Larger versions of (**b**-**f**) can be seen in Additional file 1: Figures S19, S20, S21, and S22. The names for each reaction and metabolite can be seen by zooming into the the larger versions of the maps in the Additional file

The question then arises: How does the regulation of metabolic genes affect metabolism and what role does degradation play in defining metabolic state? To connect the regulation to metabolic function, we integrated the differential expression data with the updated genome-scale metabolic model to predict change in fluxes through metabolic pathways when the organism grows on different substrates. Briefly, the coefficients of biomass components, which describe a cell’s physiological requirement for that molecule, were allowed to vary between growth substrates and fitted such that the deviation of the metabolic flux ratio from gene expression ratios of DEG in those pathways were minimized (see Additional file 1: Section “**Additional Modeling Methods and Results**” for a full description of the model; fitted biomass coefficients can be seen in Fig. 7 and Additional file 1: Figure S15). Prior to this procedure, only flux changes in methanogenesis were correlated to expression data. After fitting the biomass coefficients flux ratios were more highly correlated to expression ratios and many more pathways carried different flux (Figs. 8, 9, 10 and Additional file 1: Figure S16). The results of this procedure are hypotheses about which pathways carry more or less flux when the organism is grown in one condition compared to another. For instance, when comparing MeOH and acetate growth we find that in general most pathways carry significantly more flux when growing MeOH (Fig. 8, blue lines). Those reactions that carry more flux under acetotrophic growth outside of methanogenesis are primarily involved with biosynthesis of several amino acids (Ile, Thr, Trp, Asn, Cys) and interconversion of alcohols and aldehydes (Fig. 8. red lines). The results comparing MeOH and TMA are more varied, especially with regards to ion and metal transport and carbohydrate metabolism (see Fig. 10 f). By examining these results we can ascribe the function each differentially expressed gene has in defining the metabolic phenotype. The results contrasting the three substrates are mapped onto the metabolic network in Fig. 10 b, d, and f. The largest effect is seen in acetate growth, where the majority of biosynthetic pathways carry less flux, especially those that generate amino acids and nucleotides as well as the cobalamine and coenzyme B pathways. Vitamin and cofactor metabolism show the majority of change compared to methanol. Decreased adenosylcobalamin and coenzyme F420 biosynthesis usage in acetate compared to methanol are associated with many differentially expressed genes (see previous section). Similarly, increased coenzyme F420 biosynthesis when growing on TMA compared to MeOH is associated with differentially expressed genes (*cbiCFHL*, *cobQ*). Our procedure correctly predicted the increase of coenzyme M in acetate grown cells compared with MeOH grown cells [81]; however, there are few studies that have examined biomass composition, and the validity of our predictions remains to be tested, especially from a quantitative perspective. The modeling results show how the DEG in this study effect metabolism connecting, for the first time, regulation to metabolic phenotype for this organism. A detailed analysis of flux changes and fitted biomass coefficients can be found in Additional file 1: Section “**Additional Modeling Methods and Results**”.

### Conservation of differentially expressed genes

**Fig. 11:**
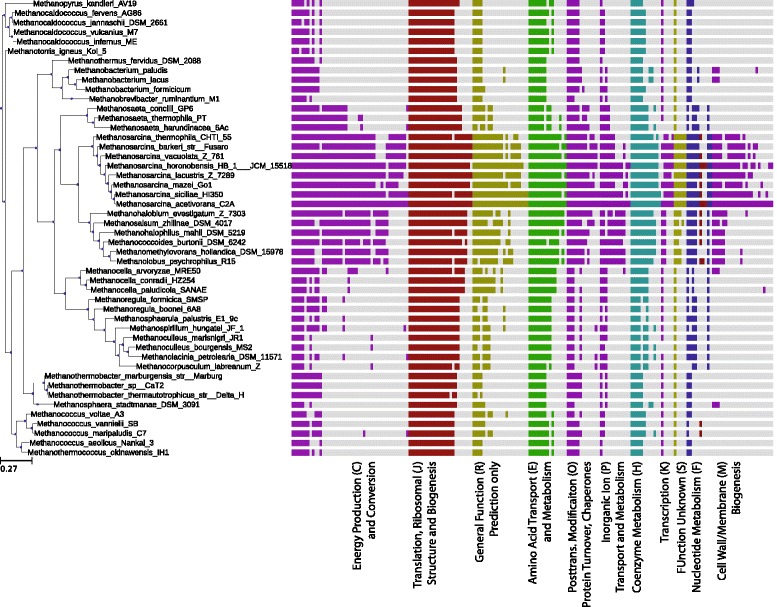
Phylogeny of Differentially Expressed Genes. Conservation of the genes that are differentially expressed between MeOH and acetate growth. Each *vertical bar* indicates that a homolog for the differentially expressed gene exists in the indicated species (computed as the bidirectional best hits functionality in the ITEP software [92] with an E-value cut-off of 10^−5^ for a database of ∼125000 proteins). Most differentially expressed genes are highly conserved among the *Methanosarcinales*; however a core set of genes are conserved across all methanogens

The similarity of mRNA degradation rates among related species grown under similar conditions—as evidenced by comparison of our results to the limited studies on *M. mazei* [66] and findings of other studies in other organisms [12, 14]—suggests that metabolic control points and regulatory modes identified in one organism could be inferred in the metabolism of similar organisms. From an evolutionary stand-point related organisms subjected to similar environments would likely retain regulatory controls that optimize their fitness. A simple analysis shows that most of the differentially expressed genes are conserved among the *Methanosarcinae* (as shown in Fig. 11, and Additional file 1: Figures S17 and S18). The amount of conservation drops off as one moves further away from *Methanosarcinae* and towards the simpler methanogens which lack significant metabolic capabilities that are found in the *Methanosarcinae*. This is clearly illustrated by the energy production (e.g. methanogenesis, electron transport) genes where the hydrogenotrophic methanogens lack significant portions of genes that are responsible for enabling the utilization of growth substrates beyond carbon dioxide. Despite the metabolic differences, a large fraction of all differentially expressed genes are still conserved across all the methanogens, especially those for translation. The problem that remains is to discover the structure and elements of the regulatory network and explore how they evolved.

## Conclusion

In this study we have demonstrated that the carbon source regulates significantly more than just methanogenesis in *Methanosarcina acetivorans*, with genes all across the genome affected especially those involved in growth (e.g. transcription, translation) and metabolism (amino acid, nucleotide and vitamin biosynthesis). We found that while mRNA half-lives scale with doubling time, the effect was not uniform across functional classes, suggesting that the cell prioritizes certain capabilities at lower growth rates most likely to account for lower available energy. By combining the expression data with the half-life data we were able to identify genes that were likely targets of transcriptional regulation (e.g. transcription factors) or degradational regulation (e.g. sRNAs, RNases, riboswitches), providing testable hypotheses that can direct molecular studies of regulation within the organism. For example, we identified ∼32 putative regulators and their targets in *Methanosarcina acetivorans* and found that about 6 of the transcription factors and their targets were highly conserved across the order of *Methanosarcinales* and some were conserved across among all the methanogens. We hypothesized functions for those regulators, based on similarly conserved genes, which can be readily tested with molecular biology experiments.

We found that most differentially expressed genes were involved in metabolism and about half of them were under degradation control. This is the first study to find such a prominent effect of degradation control in metabolism. As the RNases are not differentially expressed, this suggests that *M. acetivorans* may make extensive use of sRNAs and riboswitches to fine-tune the degradation of mRNAs that encode metabolic proteins according to their environment. We tuned coefficients of the biomass reaction of our metabolic model to increase correlation between fluxes and expression data for each carbon source. By doing so we could determine how the metabolite demand along certain pathways varies with growth substrate. This procedure allows us to account for the differential expression of genes in those pathways. Altogether the half-life, differential expression, and correlated fluxes data allows us to build a richer picture of regulation than possible with transcriptome-only studies.

This work reveals many new features about regulation and metabolism in methanogens that inspired several hypotheses for further testing. Molecular genetic studies with corresponding transcriptomic information will be necessary to clarify the role of the differentially expressed transcription factors. Future bioinformatic and genetic studies will be required to confirm the presence and define the function of post-transcriptional regulators, especially any sRNAs. Additional proteomics data could confirm the changes in pathway fluxes that we predict for growth under various substrates. Such studies will allow the construction of a combined regulatory/metabolic network model that can predict how methanogens impact everything from the gut microbiome to the global carbon cycle.

## Methods

### Experimental

#### Strains, media, and growth conditions

*M. acetivorans* C2A strain (wild-type, WWM82 [82]) was grown in single cell morphology [83] at 37 °C in high-salt (HS) medium containing either 125 mM methanol, 50 mM TMA or 120 mM acetate [63, 84]. Handling and manipulation of all strains was carried out under strict anaerobic conditions in an anaerobic glove box, using sterile anaerobic media and stocks. Growth was quantified by measuring the optical density at 600 nm (OD_600_, Milton Roy Company Spectronic 21 spectrophotometer).

#### RNA isolation procedure

RNA was isolated as previously reported [85]. Briefly, *M. acetivorans* C2A wild type was adapted to grown, TMA or acetate for 33 generations. Cells were grow in batch cultures tubes. The total RNA was isolated from mid-exponential phase cultures (OD_600_ = 0.4 for MeOH/TMA, 0.2 for acetate growth cells) using TRIzole (Invitrogen, Carlsbad, CA) and the Zymo Direct-zol RNA MiniPrep kits (Zymo Research, Irvine, CA). Specifically, at mid-exponential phase 2 mL of culture were added to 2 mL TRIzole and 4 mL ethanol was added. Samples were processed with the Zymo Direct-zol RNA MiniPrep kit and RNA was eluted at 50 *muL*. The RNA samples were depleted of the 16s- and 23s-rRNA through hybridization to complementary biotinylated oligonucleotides and were subsequently removed with streptavidin-magnetic beads (modified from [86]). Samples were stored at −80^∘^ *C*. Total RNA concentration and integrity were assessed using a Nanodrop^®^ and Agilent BioAnalyzer, respectively. The A _260/280_ ratios measured by the BioAnalyzer were generally >2.0 and Nanodrop indicated between 200 and 800 ng/ *μ* *L* RNA obtained. Triplicate biological cultures were processed for each time-point. An additional two and five cultures were processed from mid-exponential phase for TMA and methanol, respectively.

#### RNA transcription inhibition

In order to estimate half-lives for RNA transcripts, a series of RNAseq experiments were performed at timepoints after halting transcription. Transcription was halted by addition of 1 mL of ∼ 80 *μ* *M* of actinomycin D to cultures growing in exponential phase (OD_600_ = 0.4 for MeOH/TMA, 0.2 for acetate growth cells). At 6 or 7 times after transcription was halted 2 mL of culture was withdrawn (5, 10, 20, 30, 60, 120, and 240 min). RNA was isolated as described above. All half-life experiments were performed with three biological replicates.

#### Sequencing

Construction of cDNA libraries and high-throughput sequencing of RNA were carried out by the Roy J. Carver Biotechnology Center at University of Illinois at Urbana-Champaign using an Illumina HiSeq2500. All sequence data generated in this report are available online in the GEO database (accession number GSE77738). See Additional file 1: Section “**Additional Methods and Materials**” and Table S1 for details. Additionally, three RNAseq datasets were taken from the GEO database accession GSE64349.

### Computational

#### Data quality control and normalization

Quality of the RNA reads in each experiment were assessed using the FastQC tool (http://www.bioinformatics.babraham.%20ac.uk/projects/fastqc/). Individual reads with systematic sequencer error (blocks of unassignable nucleotides, N) were removed, and then reads were trimmed. The adapter sequence was trimmed, constituting between 6 and 12 bases off the head or tail of the read. In some cases 2 bases were trimmed from the end of the read when the Sanger Phred quality score at that base had a score below 20. Trimmed reads were mapped to the reference genome (accession number NC_003552 [87]) using Rockhopper v2.0.2 [46]. The software was set to map single ended reads strand-specifically to the genome. Only the highest scoring mapping for each read was retained. A minimum seed of one-third the read’s length was used and the only reads mapping more than 85% of the bases exactly were kept. Resulting reads were considered for further analysis.

Mapped reads were further processed by normalization and averaging. Two normalization schemes will be considered: 1) reads are normalized per kilobase (gene length) then per one million reads (referred to as RPKM), and 2) reads are normalized per kilobase (gene length), then averaged across operons (see section “Operon regulation”) and finally normalized per one million reads (referred to as ORPKM). After normalization, the triplicate biological replicates were averaged for each O/RPKM and the standard deviation computed. These O/RPKM values were used for subsequent analyses (see Additional file 4 for combined data).

#### Life-time fitting and RNA stability estimation

For each distinct experimental growth condition RPKM values for genes were normalized so that the superoxide dismutase (*MA1574*, *sodB*), which has been characterized as having one of the longest known half-life in the archaeon *Sulfolobus solfataricus* [13], had a half-life of 2 hours to match that measured in *S. solfataricus*. The degradation of each gene was fit to a first-order decay reaction:

1 $$\begin{array}{@{}rcl@{}} R_{i} = R_{i,0}e^{-k_{i}t} \end{array} $$

using the Levenberg-Marquardt nonlinear least-squares method in SciPy [88]. Here *R*_*i*,0_ is taken to be the RPKM for the gene *i* at time 0 and *k*_*i*_ is the decay rate. The half-life *τ*_*i*_ is then calculated:

2 $$\begin{array}{@{}rcl@{}} \tau_{i} = \frac{ln(2)}{k_{i}} \end{array} $$

Standard fitting residuals were used to compute *p*-values for the fits as well as for statistical significance testing of half-lives between and within growth conditions. Genes with uncertainty in the fitting value *τ*_*i*_ of greater than 50% were omitted from subsequent analysis. These half-lives can be found in the Additional file 5.

RNA structural stability (folding energy) was estimated for each gene by folding the open-reading frame, as annotated in the genome NCBI genome NC_003552, using the RNAFold package [89] using both the Turner 2004 [90] and Andronescu 2007 parameters [91] leaving all other parameters as their default value.

#### Differential expression calling

Differentially expressed genes (DEG) were computed using three statistical models: edgeR v3.8.5 [34], PoissonSeq v1.1.2 [35], and DESeq2 v1.6.3 [36]. For all methods the default normalization provided by the packages was used in computing the DE statistics. A description of the workflow for each of the statistical models can be found in Additional file 1: Section “**Additional Methods and Materials**” and code to reproduce the results can be found in Additional file 6. Genes with a *p*-value ≤ 0.01 were considered to be differentially expressed. Differentially expressed genes can be found in the Additional file 7.

#### Operon regulation

Four sets of putative polycistronic operon structures for the genome of *M. acetivorans* were considered. One set was predicted by Rockhopper [46], and three sets were taken from the online databases: Microbes Online [44], ProOpDB [45], and DOOR2 [47]. Mapped reads were pooled across operons and differentially expressed analysis was performed (as computed in Section “**Differential expression calling**” and described in Section “**Differentially expressed genes**”).

#### Computing evolutionary conservation

The Integrated Toolkit for Exploration of Microbial Pan-genomes (ITEP) was used to compare conservation of differentially expressed genes [92]. ITEP was used to construct a database of ∼60 methanogens with published complete or nearly complete genomes. Default parameters were used to construct the database (E–value cutoff of 1.0 and 1 ×10^−5^ for nucleic acids and proteins, respectively; MCL clustering was run with the maxbit option with inflation and score cutoffs of 2.0 and 0.4, respectively). For each differentially expressed gene in *M. acetivorans*, the top scoring homolog in each other methanogen was identified, if one exists. These were ordered by degree of conservation and plotted on a phylogenetic tree that is rooted at *Methanopyrus kandleri* using the Python ETE Toolkit [93] (see Fig. 11 and Additional file 1: Figures S17 and S18).

#### Biomass coefficient fitting procedure

A new method was developed to integrate differential expression data with the metabolic model, as existing methods to integrate expression data into genome-scale metabolic networks have been shown to perform relatively poorly unless metabolomic data was also available and integrated [94]. We reasoned that since mRNA level and protein level generally have poor correlation—in our case, a Pearson *r*=0.63—that using expression data to make quantitative predictions would be problematic. Therefore, we devised a scheme designed to make qualitative predictions about changes in metabolic flux distributions and the metabolites that the pathways produce. The method takes a single growth substrate as the reference and then varies the biomass coefficients (the required moles of metabolite to create a new gram of dry cell mass) so that the ratio of fluxes between the two predictions matches the ratio of messenger expression for differentially expressed genes. The metabolite requirement in the new condition is then classified as being higher, lower or unchanged with respect to the reference growth substrate, suggesting targets for further experiment.

More specifically the method attempts to minimize an objective function; that is:

3 $$  min\left(\sum_{i=1}^{N_{DE}} \left(\sum_{r|i\in r} \left| \frac{v_{1,r}}{v_{2,r}} - \frac{m_{1,i}}{m_{2,i}} \right| \right)\right)  $$

for each biomass coefficient *b*_*j*_. In the equation *N*_*DE*_ is the number of differentially expressed genes, $\vec {m}_{c,n}$ is the expression level of gene *n* in growth condition *c*, $\vec {v}_{c,r}$ is the flux through reaction *r* that has a gene-protein-reaction rule that contains gene *i* in growth condition *c*. In order to do this, the the list of biomass coefficients is ordered randomly, and the new optimal biomass coefficient *b*_*j,opt*_ is found in order from the beginning to the end of the list. For each biomass coefficient, the uptake rate is varied such that the experimentally measured growth rate is achieved. This whole process is performed multiple times with permuted ordering for optimizing biomass coefficients and the final biomass coefficients are selected as the best for that round of optimization.

The average and standard deviation of these biomass coefficients are computed. If the original biomass coefficient is different from the range of newly sampled biomass coefficients (*p*<0.01, t-test) the biomass coefficient is considered significantly different. If the new coefficient is larger (smaller) it indicates that metabolite is required in higher (lower) amounts than in the reference condition. A large standard deviation indicated that many different selections for that biomass coefficient could give equally good scores (Eq. 3).

We used 96 random orderings of the 67 biomass coefficients found in Fig. 7 and Additional file 1: Figure S15. Nine were significantly different comparing TMA (reference condition) to acetate, 12 were significantly different comparing MeOH (reference condition) to acetate, and 16 were significantly different comparing MeOH (reference condition) to TMA.

## Additional files

Additional file 1 Suppementary Information. Methods, results, discussion, figures and tables supplemental to the main text. (PDF 9707 KB)

Additional file 2 Metabolic Model and Maps. A zip file archive containing the *i*ST807 metabolic model in the SBML format, an Excel spreadsheet including descriptions of the metabolic model and the metabolic map in formats compatible with Cytoscape and Escher. (ZIP 1392 KB)

Additional file 3 Estimated RNA Counts Per Cell. Estimated count of each mRNA in an average methanol and acetate grown cell. (XLSX 277 KB)

Additional file 4 Read Counts. Unnormalized and normalized reads mapped onto the *M. acetivorans* reference genome. (XLSX 4782 KB)

Additional file 5 Half-Lives. A table of half-lives measured for genes in *M. acetivorans*. Only genes with positive half-life and coefficients of variation in the fit <0.5 are included. (XLSX 756 KB)

Additional file 6 Code. Code used to compute differentially expressed genes. (ZIP 3 KB)

Additional file 7 Differentially Expressed Genes. Differntially expressed genes as called by edgeR, DESeq2 and PoissonSeq as well as the consensus set of all three methods. Includes fold change and p-values. (XLSX 370 KB)

## Abbreviations

COG: Clusters of orthologous groups
DEG: Differentially expressed genes
GSEA: Gene set enrichment analysis
MeOH: Methanol
mRNA: Messenger RNA
ORF: Open reading frame
PCA: Principle component analysis
TBP: TATA binding protein
TCA: Tricaboxylic acid cycle
TMA: Trimethylamine
tRNA: Transfer RNA
sRNA: Small RNA
