# Supplementary material for: Genome-wide gene expression and RNA half-life measurements allow predictions of regulation and metabolic behavior in Methanosarcina acetivorans
Source: BMC Genomics. 2016 Nov 16;17:924. doi: 10.1186/s12864-016-3219-8 (PMC5112694; doi:10.1186/s12864-016-3219-8)
Supplement: Additional file 1 — Suppementary Information. Methods, results, discussion, figures and tables supplemental to the main text. (PDF 9707 KB) [file 12864_2016_3219_MOESM1_ESM.pdf]

1 SUPPLEMENTARY INFORMATION

1.1 Additional Methods and Materials

Each RNA sample came from an independent culture grown to exponential phase with an OD_{600} about 0.2 or 0.4 for slow (acetate) and fast-growing (MeOH, TMA) substrates, respectively. RNAseq was performed on a total of 69 RNA samples. Dataset descriptions and accession numbers may be found in Table S**S1**. With the exception of two TMA samples, the library preparation was carried out using the ScriptSeq™v2 kit from EpiCenter. The two TMA samples were prepared with the Illumina TruSeq™v2 kit. Three samples for growth on methanol were generated in a previous report [57]. RNA isolation was performed as described in the methods section of the manuscript. Mapped reads in each sample were well fit by log-normal with a standard deviation of ~1.0.

1.1.1 Differential Expression Calling Procedures

A total of 16 datasets were considered: 8 replicates for MeOH, 5 for TMA and 3 for acetate. The zero timepoint from the degradation study accounts for three datasets in each growth condition. Additionally, 5 MeOH and 2 TMA replicates were obtained for exponentially growing cultures. Three methods were used for calling differentially expressed genes: DESeq2, edgeR and PoissonSeq. These three methods were chosen due to their relatively different assumptions about underlying distributions and method for normalization. Trimmed and mapped reads were loaded as datasets into each program without normalization. Multifactor statistical modeling was used when performing differential expression calling, where available, taking the growth substrate as the first factor and the library preparation method as the second factor. This step is necessary as 67% of the total variation observed in the experiment when multifactor design is not considered is due to the library preparation kit. When using the two factor design, the first principle component accounted for variation between methylotrophic and acetotrophic growth, and the second accounted for differences between methylotrophic growth substrates TMA and MeOH (Fig. **S1**).

Computations were performed using the R packages edgeR v3.8.5 [34], PoissonSeq v1.1.2 [35], and DESeq2 v1.6.3 [36]. Genes were considered differentially expressed when the p-value was < 0.01. All differentially expressed genes can be found in Supplementary Table “DifferentiallyExpressedGenes.MultiFactor.xlsx". All R scripts used to perform differential expression calling may be found in the Supplementary File “DEGComputation.zip". Differential expression calling procedures were as follows:

DESeq2 – The library preparation was defined as the first experimental factor, and growth substrate as the

Table S1 A listing of all RNA-seq datasets used in the study. The first column designates the name of the sample file, followed by the growth condition, and the GEO database accession number for the sample.

Sample	Condition	Accession Number
	Steady-State[a]	
LK1_ATCACG_L007_R1_001	MeOH[c]	GSM2058125
LK9_TTAGGC_L003_R1_001	MeOH[c]	GSM2058137
LK17_GGCTAC_L004_R1_001	MeOH[c]	GSM2058150
PK19_CAGATC_L00M_R1_001	MeOH	GSM1569045
PK20_ACTTGA_L00M_R1_001	MeOH	GSM1569046
PK21_GATCAG_L00M_R1_001	MeOH	GSM1569047
Metcalf_C2AM1_R1.PF	MeOH	GSM2058211
Metcalf_C2AM3_R1.PF	MeOH	GSM2058212
LK25_ATCACG_L003_R1_001	TMA[c]	GSM2058193
LK31_CAGATC_L004_R1_001	TMA[c]	GSM2058199
LK37_ATCACG_L005_R1_001	TMA[c]	GSM2058205
Metcalf2_C2AT_R1.PF	TMA[d]	GSM2058213
Metcalf2_C2AT_R2.PF	TMA[d]	GSM2058214
LK43_CAGATC_L006_R1_001	Acetate[c]	GSM2058164
LK49_ATCACG_L007_R1_001	Acetate[c]	GSM2058175
LK55_CAGATC_L008_R1_001	Acetate[c]	GSM2058187
	RNA Degradation Study[b]	
LK2_CGATGT_L007_R1_001		GSM2058127
LK10_TGACCA_L003_R1_001	MeOH 5min	GSM2058139
LK18_CTTGTA_L004_R1_001		GSM2058152
LK7_ATCACG_L003_R1_001		GSM2058128
LK11_ACAGTG_L003_R1_001	MeOH 10min	GSM2058141
LK19_ATCACG_L005_R1_001		GSM2058154
LK3_TTAGGC_L007_R1_001		GSM2058130
LK12_GCCAAT_L003_R1_001	MeOH 20min	GSM2058143
LK20_CGATGT_L005_R1_001		GSM2058156
LK4_TGACCA_L007_R1_001		GSM2058132
LK13_CAGATC_L004_R1_001	MeOH 30min	GSM2058145
LK21_TTAGGC_L005_R1_001		GSM2058158
LK5_ACAGTG_L007_R1_001		GSM2058134
LK14_ACTTGA_L004_R1_001	MeOH 60min	GSM2058147
LK22_TGACCA_L005_R1_001		GSM2058160
LK6_GCCAAT_L007_R1_001		GSM2058136
LK15_GATCAG_L004_R1_001	MeOH 120min	GSM2058148
LK23_ACAGTG_L005_R1_001		GSM2058162
LK26_CGATGT_L003_R1_001		GSM2058194
LK32_ACTTGA_L004_R1_001	TMA 20min	GSM2058200
LK39_TTAGGC_L005_R1_001		GSM2058206
LK27_TTAGGC_L003_R1_001		GSM2058195
LK33_GATCAG_L004_R1_001	TMA 30min	GSM2058201
LK40_TGACCA_L005_R1_001		GSM2058207
LK28_TGACCA_L003_R1_001		GSM2058196
LK34_TAGCTT_L004_R1_001	TMA 60min	GSM2058202
LK41_ACAGTG_L005_R1_001		GSM2058208
LK29_ACAGTG_L003_R1_001		GSM2058197
LK35_GGCTAC_L004_R1_001	TMA 120min	GSM2058203
LK42_GCCAAT_L005_R1_001		GSM2058209
LK30_GCCAAT_L003_R1_001		GSM2058198
LK36_CTTGTA_L004_R1_001	TMA 240min	GSM2058204
LK75_TTAGGC_L005_R1_001		GSM2058210
LK44_ACTTGA_L006_R1_001		GSM2058166

second factor. DESeq2 was run using the parallel implementation and the option “addMLE=TRUE", contrasting the growth conditions. Differential expression statistics were sorted by the adjusted p-value (Benjamini–Hochberg method) and stored to file. Additionally, a variance stabilizing transformation was performed on the data before a PCA analysis. The first two principle components were plotted to show the separation by growth types (Fig. **S1**).

edgeR – The library preparation was defined as the first experimental factor followed by the growth condition. Normalization factors were computed and a generalized linear model (GLM) was estimated. The dispersion trend over multiple genes was then calculated followed by the per gene (tag–wise) dispersion. Finally, the GLM model was fit, and the adjusted p-value (Benjamini–Hochberg method) was computed and the data stored.

PoissonSeq – Total mapped reads for each gene were scaled by a factor 0.1 so that the PoissonSeq method did not overflow. The differential expression calling routine was run with the “pair parameter" set to false and the data type taken to be “two–class" (substrate and library preparation kit). A total of 100,000 permutations were performed per comparison. Data was sorted by adjusted p-value (using the PoissonSeq default method: permutation plug–in) and stored to file.

1.1.2 Uncertainty in Differentially Expressed Genes

A nonparametric bootstraping approach was used to estimate the uncertainty in the number of differentially expressed genes. Briefly, the DESeq2 workflow was applied to subsets of the all the RNAseq data sets and the counts of DEG were enumerated. All combinations of sets of RNAseq data ranging from two to the maximum number of replicates were generated for each growth condition (MeOH, TMA and Acetate). The Cartesian product of these sets were generated, and the DESeq2 workflow was used to estimate the total number of differentially expressed genes between the three conditions. For a given total count of datasets ($N_{MeOH}+N_{TMA}+N_{Acetate}$), the average and standard deviation in the number of genes called as differentially expressed with confidence p$\leq$0.01 were computed. Mathematically,

$$\forall c \in \{\text{MeOH}, \text{TMA}, \text{Acetate}\} \quad \textbf{(S1)}$$

$$\forall i \in \{2, ..., N_c\} \quad \textbf{(S2)}$$

$$S_c = \bigcup_i \left\{ \binom{N_c}{i} \right\} \quad \textbf{(S3)}$$

$$P = S_{\text{MeOH}} \times S_{\text{TMA}} \times S_{\text{Acetate}} \quad \textbf{(S4)}$$

where c is the condition, N_c is the count of datasets measured in that condition, S_c is the set of all combinations

containing 2 to N_c datasets and P is the product of all unique datasets. This accounted for about 26,000 sets of differential expression combinations. At each $N \in 2, N_c$ the coefficient of variation in number of DEG was computed (Fig. **S2**). This analysis demonstrates that the CV decreases as additional datasets are included. The CV is a measure of the uncertainty in the set of DEG; we estimate an uncertainty of 24-30% when using all 16 datasest.

1.2 Additional Experimental Results

1.2.1 Half-Life Data

To assess reproducibility of the experimental procedure, correlations were computed across timepoints. The profiles were averaged across all three replicates before correlations were computed. As can be seen from the correlation matrix in Fig. **S3**a, MeOH, TMA, and acetate correlate highly within condition and cluster together.

Limited data for half-lives of genes in *Methanosarcina* currently exist. Only half-lives for 5 genes have been reported from the related organism *Methanosarcina mazei* zm-15 [67] where they measured the half-lives of the methyltransferase genes (*mtaA1*, *mtaCB1*) from methanol grown cultures and acetoclastic genes (*pta*, *ack*) from acetate grown cultures. As can be seen in Figure **??**A, the half-lives we measured agree quite well and within one order of magnitude in the worst case. These differences might be due to uncertainty in the measurements, differences inherent to the different organisms, and the fact that Cao et al. measured half-lives at 30°C—and showed that there are different temperature stabilities for transcripts—while we measured half-lives from cells growing at 37°C. In general, they agree quite well and provide confidence in our measurements. The comparison also highlights an interesting fact, that the different transcripts have different stabilities after being grown in different conditions. This supports our hypothesis that half-lives are differentially stabilized/destabilized in different conditions and wy the control coefficients are important to compute, likely by post-transcriptional modification as Cao et al. concluded [67] or via small RNA regulation.

A scaled value for each of the half-lives was computed for each condition. The scaled half-life value is computed as $HLs = HL/(DT \times 60)$ where the HL is the unscaled half-life and DT is the doubling time in that condition in units of hr. Growth rates used for scaling of data for were taken as the average of experimentally determined values reported in literature. For MeOH, TMA and acetate, growth rates used were 7.5hr [95, 96, 43], 8.9hr [43] and 24.6hr [97, 95, 43], respectively. This scaled half-life represents the fraction of the cell cycle that a transcript will remain stable. As Fig. **S4** demonstrates, regardless of the growth substrate, on average the RNA molecules persist for about the same fraction of the cell cycle. The scaled half-lives are statistically the same across all conditions ($p>0.33$, t-test) with an average value of 12.7%±3.5% of the cell cycle. Because growth rate is linearly proportional to ATP production rate [48], and it is generally assumed that growth rate and

growth yield are co-optimized in prokaryotic organisms [98], we can hypothesize two scenarios that cause this constant fraction of the cell cycle. First, the cell is optimized to use as little ATP as possible while maintaining a level capable of allowing the translation of new proteins at the correct rate and thus is linearly proportional to the ATP production rate. Since RNA turnover is a trade-off between degradation rate and production rate, the latter of which should be directly proportional to the energy required to ligate nucleotides into new RNA molecules, their steady-state values should be proportional to ATP production. An alternative but related second scenario is also possible; namely, that RNA maintains a constant fraction of the total cellular weight regardless of the growth rate. In this scenario, it is not the ATP consumption requirement in RNA production, but instead the production and degradation kinetics that set the steady-state amount of RNA in a cell. Since cell mass is proportional to ATP production rate and growth rate, the steady-state RNA is indirectly related to ATP production rate through the maintenance of constant mass fraction. One might argue that because the ATP cost of RNA production is such a low fraction of total energy expenditure, the latter of these two explanations is more likely.

1.2.2 Differentially Expressed Genes

The coefficient of variation (CV) computed using our uncertainty estimation method (Supplemental Section **Uncertainty in Differentially Expressed Genes**) decreases as the number of RNAseq dataset replicates increase (Fig. **S2**). The falling CV is consistent with prior studies that showed for DESeq (the precursor to DESeq2) as well as other similar methods such as edgeR and PoissonSeq, the sensitivity rate (fraction of true positives) increases with number of replicates [99, 100]. At 15 datasets, the CV is about 30% of the mean. Linearly extrapolating the trends to 16 datasets results in a CV of 24% of the mean. Therefore, we estimate that the uncertainty in the number of differentially expressed genes is between 24 and 30% of the total number.

Among the differentially expressed genes, four putative regulatory proteins stood out: *MA0866*, *MA1395*, *MA2212* and *MA4346*. We compared the expression profiles across the three substrates to DEG that had nearly the same pattern of conservation (±2 genes) and these genes were found to be highly correlated/anticorrelated to the regulators(Fig. **S14**). Analysis of these similarly conserved genes leads to interesting predictions that the regulators could be either directly regulating the group of genes, or is coregulated with them by another transcription factor.

The first highly conserved regulator *MA0866* encodes a PhoU type protein that likely plays a role in phosphate uptake. As expected, its expression is highly correlated with a phosphate related genes including a phosphate transporter subunit (*pstS*, *MA0889*), nicotinate phosphoribosyltransferase (*pncB*, *MA2533*) as well as TCA cycle enzymes citrate synthase (*MA0249*) and malate dehydrogenase (*mdh*, *MA0819*). Additionally,

it is anticorrelated to the gene responsible for the final step of lysine synthesis (*lysA*, *MA0762*). These results suggest the gene could play a role in maintaining phosphate and energy balance in the cell, if it were to regulate these enzymes.

MA2212 is a TrmB-like regulator. It is notable because it is correlated highly with acetotrophic genes *ack* (*MA3606*), *pta* (*MA3607*), and *cam* (*MA2536*) as well as subunits of the ATP synthase (*MA2433*/*MA2435*/*MA2440*)

The final regulator with high correlation to genes with similar conservation *MA4346* was specific to the family *Methanosarcina* but most genes that were similarly had nonspecific or no annotated function.

1.2.3 Estimating mRNA Levels

An estimate of the average copy number of each mRNA in an average cell, N_i for each of the three growth substrates were computed using the following equation,

$$N_i = x_{\mathrm{RNA}} \cdot \rho_{\mathrm{cell}} \cdot V_{\mathrm{cell}} \frac{a_i}{m_i} \tag{S5}$$

subject to the constraint

$$m_{RNA} = \sum_i^N a_i m_i \tag{S6}$$

where a_i is the fraction of total mRNA mass m_{RNA} that the transcripts from a single gene accounts for, which is taken to be linearly proportional to the RPKM values from the RNAseq data; ρ_{cell} is the density of an *E. coli* cell taken from the CyberCell Database [101]; V_{cell} is the volume of the cell computed from our previous characterization of cell dimensions [85]; x_{RNA} is the mass fraction of total cell mass that is RNA, which is taken from the metabolic model (24% of total cell dry mass) [48]; N is the total number of genes considered in the analysis; and m_i is the molar mass of the transcript of interest. Since the volume of cells grown in TMA were not measured, but the growth rates are similar to those for cells grown in MeOH, the volume were assumed to be the same. Total RNA for a single “average" cell was estimated to be 23.8 and 10.6 fg from MeOH/TMA and acetate grown cells, respectively. The counts of each RNA estimated using this analysis for the three substrates can be found in Supplementary File “EstimatedRNACounts.xlsx".

As a quality check, the numbers computed through this analysis were compared to those that were reported in Cao et al [67] measured for *M. mazei* growing in MeOH and acetate using RT-qPCR as shown in Fig. **S13**. In Cao et al [67], the values were originally reported per 100,000 16s-rRNA transcripts. We rescaled these

numbers to be proportional to 14,000 rRNA transcripts, the average number of ribosomal protein Rpl18p count we measured previously [85] using a single-molecule pulldown [102]. As can be seen in the figure, all transcript counts except *mtaA2*, *mtaB2*, and *cdhB* are statistically indistinguishable, suggesting that estimates for mRNA numbers are good. The minor disagreement for the three transcripts could be due to the difference in the two species.

1.2.4 Control Coefficients

The confounding effects of changes in transcription and degradation rates on average mRNA level that occur with changes in growth rate can be deconvoluted. We attempt to estimate the effect of each by using a recently reported method that computes the extent that transcription and degradation have in setting the steady-state level of mRNA in a cell [7, 8, 9]. The analysis is based on the assumption that the cell is at steady-state, implying the transcription rate and the degradation rate are balanced, or

$$k_{trn} = \gamma \cdot M + \mu \cdot M, \tag{S7}$$

where k_{trn} in the transcription rate, γ is the mRNA's degradation rate constant as computed from RNAseq data, μ is cell growth rate and M is the average copy number of a transcript. The transcription rate—which is a proxy for change in growth rate (ribosomal count, etc.) and changes in promotion or repression of a gene—and degradation rate—changes due to active or passive degradation by RNAses or post-transcriptional control by sRNA—are computed per mRNA. Writing down the total differential and manipulating, the contribution of each process can be estimated as

$$dM = \frac{dk_{trn}}{\gamma + \mu} - k_{trn}\frac{d\gamma}{(\gamma+\mu)^2} - k_{trn}\frac{d\mu}{(\gamma+\mu)^2}. \tag{S8}$$

Rearranging and noticing that at steady state, $M = k_{trn}/\gamma$ yields

$$\frac{dM}{M} = \frac{dk_{trn}}{k_{trn}} - \frac{d\gamma}{\gamma+\mu} - \frac{d\mu}{\gamma+\mu} \tag{S9}$$

which can then be written as a relation between relative changes in transcript count due to changes in each rate,

$$1 = \frac{dlnk_{trn}}{dlnM} - \frac{dln\,(\gamma+|\mu|)}{dlnM} - \frac{dln\,(\mu+|\gamma|)}{dlnM}, \tag{S10}$$

where here we use $|\cdot|$ to denote that this value is held constant in this term. Two cases for this equation can be considered: 1) the degradation of mRNA due to dilution is negligible, $\gamma >> \mu$, and 2) degradation due to

dilution cannot be neglected. In the former, the contributions are due to transcription ρ_T and degradation ρ_D, or

$$1 = \frac{dlnk_{trn}}{dlnM} - \frac{dln\gamma}{dlnM} \quad \textbf{(S11)}$$

$$= \rho_T + \rho_D. \quad \textbf{(S12)}$$

In the latter, the contributions are due to transcription ρ_T, degradation ρ_D and growth (dilution) ρ_G, or

$$1 = \frac{dlnk_{trn}}{dlnM} - \frac{dln(\gamma+|\mu|)}{dlnM} - \frac{dln(\mu+|\gamma|)}{dlnM} \quad \textbf{(S13)}$$

$$= \rho_T + \rho_D + \rho_G. \quad \textbf{(S14)}$$

The majority of mRNA satisfy $\gamma >> \mu$. Therefore, we proceed neglecting ρ_G. Dressaire et al. applied Eqn. **S11** to *L. lactis* growing in chemostats at different rates and found that only a few percent of genes are degradationally controlled [8]. Esquerré et al. applied the same analysis to *E. coli* growing in chemostats at several different rates [7]. Both studies ignored the dilution effects, citing the small average half-lives relative to the doubling times studied (1-8%) similar to our average 12.7%. In contrast to these studies, we found a significantly higher number of genes that appear to be degradationally controlled (16-28%). This percentage was even higher when considering only genes associated with metabolic reactions (48-60%). Control coefficients calculated between each of the three growth substrates can be found mapped onto the metabolic network in Figs. **S19** and **S20**.

1.3 Additional Modeling Methods and Results

1.3.1 Modifications to metabolic model

COBRAPy [103] was used to handle the flux balance computations and all changes to the metabolic model. The *M. acetivorans* model (*i*MB745) [48] required additional improvements in order to accurately predict the metabolic behavior when grown in the standard high-salt medium [63] used for the RNA seq experiments. All components of this medium that could be taken up by the metabolic model are listed below and were turned on with a default lower bound of -1000 $\frac{mmol}{gDW \cdot h}$.

In addition to refining the methanofuran biosynthesis pathway, the alternate aminoacylation pathway for cysteine and the pyrrolysine biosynthesis pathways—two evolutionarily significant pathways—were added to the

Table S2 Components of high-salt medium used to grow methanogens [63]. Starred (*) metabolites currently do not have exchange uptake reactions in model.

Core components	NaCl, MgCl, $CaCl_2$, $NaHCO_3$, KCl, KH_2PO_4, NH_4Cl, Na_2S^*, Cysteine, resazurin*
Trace elements	$Fe(NH_4)_2(SO_4)_2$, $CoCl_2$, $MnSO_4$, Na_2MoO_4, Na_2WO_4, $ZnSO_4$, $NiCl_2$, $CuSO_4$, $H_3BO_3^*$, $Na_2SeO_3^*$, nitrilotriacetic acid*
Vitamins	p-aminobenzoic acid, Ca pantothenate, riboflavin, thiamine HCl, biotin, folic acid, vitamin B_{12}, pyridoxine HCl*, α-lipoic acid*, nicotinic acid*

model. Addtionally, reactions to allow uptake of methylmercaptopropionate (MMPA) were added, and the gene-reaction-protein rules for methylated sulfur compound metabolism were significantly revised according to new genetic evidence [57]. The new metabolic model *i*ST807 maintains its predictive capability from the previous model and reproduces the methanogen's inability to grow on methylamine substrates without pyrrolysine. The resulting model growth rates, methane production, and carbon dioxide production are in good agreement with the experiments (Fig. **S11**).

Alternate cysteine aminoacylation pathway In 2005, O'Donoghue, et al. [50] predicted the existence of the alternate cysteine aminoacylation pathway within a handful of methanogens, including *M. acetivorans*, which was later confirmed [51]. This indirect charging pathway for cysteine, shown in Fig **S9**, is unique to archaeal species. In certain methanogens, such as *M. jannaschii*, it is the only mechanism to charge cysteine onto its tRNA. Many archaeal species obtained the canonical cysteine charging pathway through horizontal gene transfer, explaining the presence of both pathways within *M. acetivorans.* Since *i*MB745 did not include this evolutionarily significant alternate cysteine charging pathway, it was incorporated into the modified metabolic model.

Briefly, SepRS acylates the precursor O-phosphoserine onto the cysteinyl-tRNA, then SepCysS converts the acylated O-phosphoserine into cysteine. *i*MB745 originally had the SepRS reaction only, leaving this pathway incomplete. The SepCysS reaction was added to the model and connected to the canonical cysteine aminoacylation reaction to complete the alternate pathway (Fig. **S9**). It is important to note that, to our knowledge, the actual sulfur source in the SepCysS reaction remains unknown. However, it has been shown that sodium sulfide provides the highest activity *in vitro* [104]. We decided to use hydrogen sulfide as the sulfur source because it is a sulfide produced within the organism. The parsimonius FBA solution of the model with this modification interestingly showed that it only uses the canonical charging pathway even if both pathways are turned on and will only use the alternate pathway if the canonical pathway is knocked out. The RPKM values from the RNAseq data, however, suggests that SepRS is expressed at least twice as much as CysRS on average

across MeOH, Acetate, and TMA (Table **S10**). In order to constrain the pathways to reflect this, flux variability analysis was first used to determine the allowable flux ranges for SepRS and CysRS. The maximum allowable flux through CysRS was then set to equal half that of SepRS. In *i*MB745, allowing any cysteine uptake led to an overproduction of ATP and resulted in unrealistically high growth rates. To reflect media component consumption accurately in addition to the SepRS/CysRS constraints, *i*ST807 constrains the cysteine uptake at a maximum rate of 0.35 $\frac{mmol}{gDWh}$ which is the uptake rate that minimized the differences between simulated and experimental growth rate on methanol, acetate, and carbon monoxide. Dynamic flux balance analysis was also performed to verify that this cysteine is not growth-limiting over a period of about 38 hrs as consistent with experiment [39]. These constraints successfully forced flux through both cysteine aminoacylation pathways.

Pyrrolysine Biosynthesis Pathway MMA methyltransferase is responsible for activating MMA for a methyl transfer to a cognate corrinoid protetin. In 2002, the crystal structure [105] of this enzyme from *M. barkeri* revealed the presence of pyrrolysine (pyl) within the catalytic site. Later studies by Mahapatra, et al. [106] on *M. acetivorans* showed that this methanogen could not grow on any methylamine substrates (MMA, DMA, and TMA) without the gene for pyl-tRNA, demonstrating that pyl is required for growth on methylamine substrates. The pathways involving pyl synthesis in *i*MB745 were present but flawed. First, the pyl-tRNA charging pathway mistakenly used the alanyl-tRNA instead of the known pyl-tRNA. Second, the hypothesized pyl biosynthesis pathways were outdated and no fluxes ran through them even when successfully simulating the model on methylamine substrates. The model essentially allowed for growth on methylamine substrates without the synthesis of pyl anywhere. These two errors were fixed by replacing the alanyl-tRNA with pyl-tRNA in the pyl aminoacylation pathway and replacing the pyl biosynthesis pathway with the most recent and accepted pathway from Gaston, et al. [107] shown in Fig. **S8**.

In order to force the model to recognize that pyl is required for growth on methylamine substrates, the biomass reaction was modified to reflect amino acid use more accurately. The first modification altered the biomass reaction to draw in aminoacylated tRNAs instead of free amino acids, keeping the coefficients the same. This change was based on the realization that it is amino acids charged onto their tRNAs that eventually become part of the cell biomass through protein synthesis rather than any free amino acid produced in the cell. The second modification adds a pyl-tRNA term to the biomass when simulating growth on methylamine substrates. For non-methylamine growth, the model sets the coefficient for pyl-tRNA to zero. For methylamine growth, the model turns on the coefficient. This coefficient was estimated from the approximate number of CTA codons in the *M. acetivorans* genome. CTA is canonically a stop codon but regulatory mechanisms exist within *M. acetivorans* to express this as the pyl amino acid. Since the fraction of CTA codons actually coding for pyl is unknown in this methanogen, it was taken to be 50% as an upper limit estimate. This gives a pyl-tRNA

biomass coefficient of 0.081 $\frac{mmol\ pyl}{gDW protein}$.

Biosynthesis Pathway During the curation of *i*MB745, the methanofuran biosynthesis pathway for *M. acetivorans* was largely hypothesized based on the known pathway from *M. janaschii* at the time. Since then, concrete experimental evidence documenting gene-reaction associations for the methanofuran biosynthesis pathway in *M. janaschii* has been published by the White lab [52, 53, 54, 55]. The homologous genes *MA4436*, *MA0636*, and *MA1475* in *M. acetivorans* were identified and respectively incorporated into the model for the reactions MFRS1, MFRS2, and MFRS3. MFRS6 and MFRS7 were deleted from the model to match the experimentally verified pathway in *M. janaschii*.

Adding Osmolytes to Biomass Reaction In 1995, Sowers and Gunsalus [61] published a study in which they measured the concentrations of unbound cations within *Methanosarcina spp.* in media with varying osmolarity. It was found that Mg^{2+}, Na^{+}, B, Zn^{2+}, Ca^{2+}, Fe^{2+}, Ni^{2+}, and Co^{2+} concentrations remained relatively constant despite the changing extracellular osmolarities. These unbound cations were incorporated into the biomass reaction with the coefficients: K^{+}, 0.4; Mg^{2+}, 0.163; Na^{+}, 0.017; B, 0.012; Zn^{2+}, 0.011; Ca^{2+}, 0.0038; Fe^{2+}, 0.0035; Ni^{2+}, 0.011; and Co^{2+}, 0.001 mmol/gDW. The osmolyte N-epsilon-acetyl-beta-lysine was included in a the biomass expression at a ratio of 1.11 mmol/gDW.

Adding Gluconeogenesis Intermediates/Products to Biomass Reaction A recent paper measured glycogen, gluconeogenesis fluxes and gluconeogenesis intermediate concentrations in *M. acetivorans* growing on methanol in exponential growth and stationary phase [62]. The glycogen content was significantly higher than assumed in the *i*MB745 model. As such, we have added/updated the biomass coefficients for glycogen and these intermediates based on these new quantitative measurements. The high glycogen content ($\sim$ 0.93651mmol/gDCW), consumes significant energy of the cell during growth; therefore, the ATP maintenance cost had to be lowered to match growth experiments. A final value of 44.1 mmolATP/gDCW. Comparing this value to the previous value of 65.0 mmolATP/gDCW indicates that nearly 33% of energy derived by the methanogen is used in storing glycogen. This could confer evolutionary advantage when nutrients are scarce.

1.3.2 Modelling Alternate Biomasses for Different Growth Substrates

Biomass for growth on acetate and TMA were fit taking MeOH to be associated with the published biomass coefficients. Additionally, acetate was fit using TMA as the starting biomass coefficients. Fit biomass coefficients can be seen in Figure **S15**. Flux comparisons for MeOH vs Acetate and MeOH vs TMA can be seen in Figs. **S21** and **S22** respectively, where it is demonstrated that significant changes to fluxes in amino acid and

cofactor biosynthetic pathways are predicted. Many coefficients can vary significantly, as indicated by the large standard deviations and it is unclear as to whether physiologically requirements differ. However, a handful of biomass components were statistically different ($p<0.01$, t-test, $n=96$) in the second condition compared with the first, possibly suggesting a different physiological requirement (a greater or smaller fraction of total biomass when growing in one media compared to another). When comparing methylotrophic to acetotrophic growth, our fitting procedure suggests that nickel, cobalt and AMP requirements decreases, while cellular zinc and potassium increase. The cobalamin cofactors in methyltransferases contain cobalt and the downregulation of these enzymes under acetotrophic growth is consistent with a decrease in requirement for cobalt [39]. The decreased requirement for nickel is counterintuitive as methyl-coenzyme M reductase and carbon monoxide dehydrogenase are both nickel containing and upregulated on acetate growth. Decrease in AMP requirement could be due to slower growth relating to the phosphate balance.

The average coenzyme M (CoM) increases by a factor of 10 going from MeOH to acetate, similar to results from a recent paper that showed CoM, and sulfide content in general, increases roughly by a factor of 2.8–3x for acetate grown cells [82]. Interestingly, glycogen galactan and polyglucuronate are predicted to be produced at higher levels, along with phosphorylated *myo*-inositol phospholipids, which are possibly used in producing extracellular matrices that are common in cell aggregates for acetate grown *M. acetivorans* [97].

The fitting procedure suggests a decreased biomass requirement for adenosylcobalamin and coenzyme B (CoB) when grown in acetate as compared to TMA. The former can be explained by the fact that methyltransferase which are composed of at least one corrinoid cofactor are severely downregulated when grown on acetate (and generally not of use to acetotrophic methanogenesis). Most cofactors were required in higher levels for growth on TMA than on MeOH, including coenzyme A, both the adenosinyl- and guanosinyl-coenzyme F390 analogs, succinyl-CoA and tetrahydrofolate. The reason for these increases is unclear.

The modeling indicated that several variants of coenzyme F420 was generally lower while grown on MeOH than on acetate or TMA, however the reason for this is unclear. Reports in literature on various *M. barkeri* and *M. mazei* strains are conflicting; one study indicating that F420 concentrations are significantly higher comparing MeOH to acetate grown cells [108], while other studies found a higher level for acetate grown cells [109, 110, 111]. Interestingly, one report found that different variants of the coenzyme F420 predominated in different *Methanosarcina* species. Further experiment and modeling is required to uncover the role of the various analogs and their regulation.

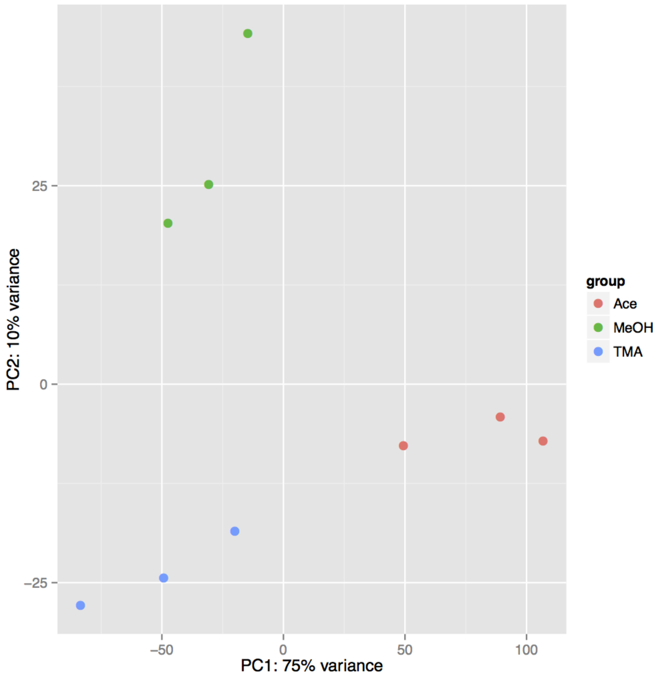

Figure. S1 Principle component analysis results of the zero time RNA expression datasets computed via DESeq2. The first component separates methylotrophic and acetotrophic growth accounting for most of the variation seen, while the second component distinguishes the two methylotrophic substrates. The PCA reaffirms the traditional classification of acetotrophic and methylotrophic growth as being orthogonal.

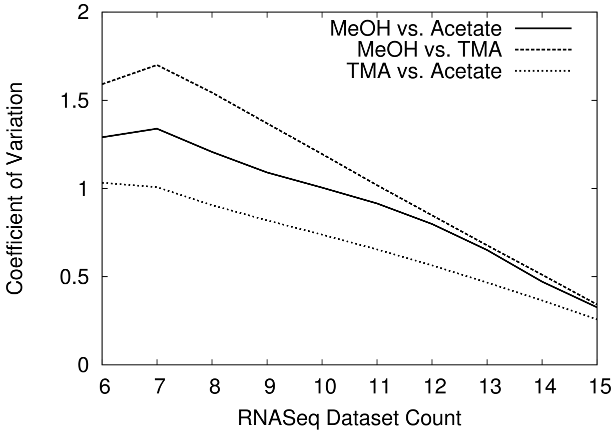

Figure. S2 Estimated coefficient of variation in the number of differentially expressed genes as a function of the number of RNAseq datasets considered as computed in Section S1.1.2. The CV decreases as the number of datasets increases, suggesting the method become converge on a consistent set of differentially expressed genes. Using these curves we estimate a CV of about 0.25–0.3 when using all 16 datasets.

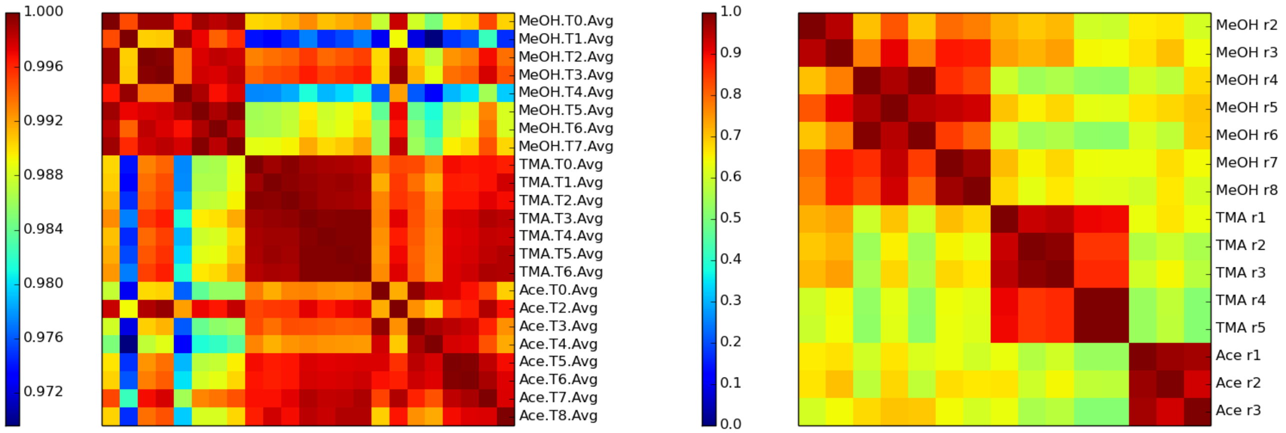

(a) **(b)**

Figure. S3 Pearson correlation matrices. (A) Comparison at various time points after transcriptional arrest. Each pixel represents the correlation between the averages of from three replicate measurements, computed over the full expression profiles. (B) Comparison of data normalized using the procedure of DESeq2 [36] for the 8 methanol, 5 TMA and 3 acetate replicates. The three growth conditions form obvious clusters, supporting the idea that the experiments were reproducible. Color bars shows the magnitude of the correlation coefficient.

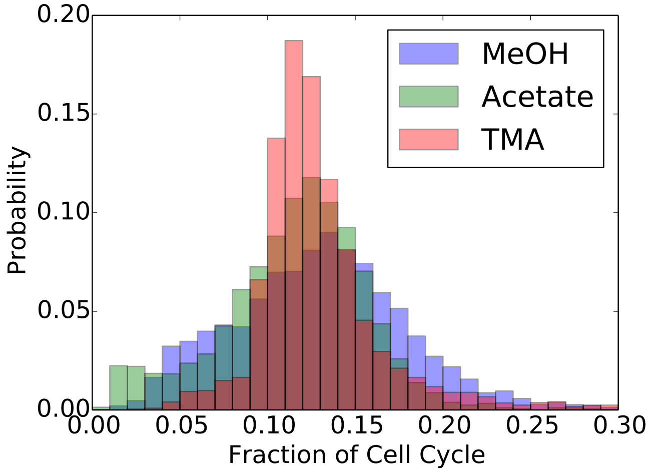

Figure. S4 The distributions that result after the half-lives have been normalized by doubling time in the respective condition. The scaled half-life is a measure of the fraction of the cell cycle that an RNA molecule is likely to persist. As can be seen, the scaled distributions overlap and the mean fraction of the cell cycle that an RNA persists (0.127±0.035) are not statistically different (p>0.33, t-test.)

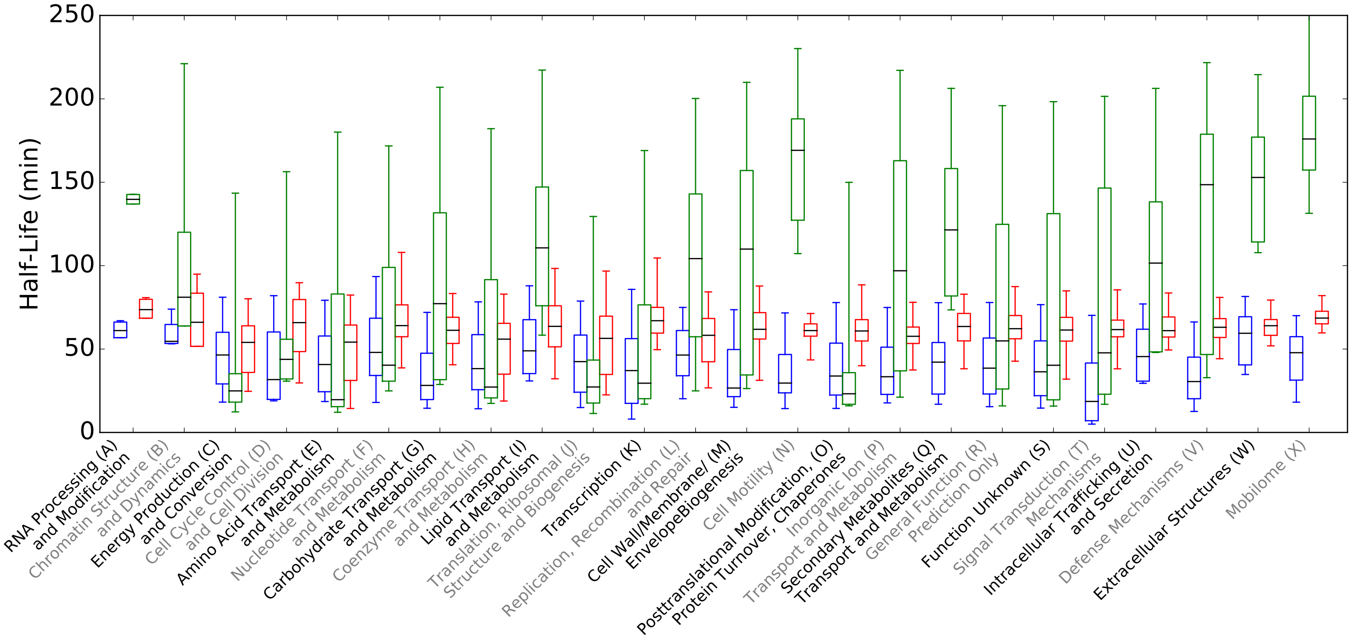

Figure. S5 mRNA half-life statistics by class showing median and quartiles for MeOH (blue), acetate (green) and TMA (red) growth. Percentiles were computed using the weighting method of Edgeworth [112]. The overall range (whiskers) of the distributions are generally the same across classes, however the quartiles and median can be significantly different, supporting the conclusions in the main text that mRNAs are selectively stabilized/destabilized depending on function.

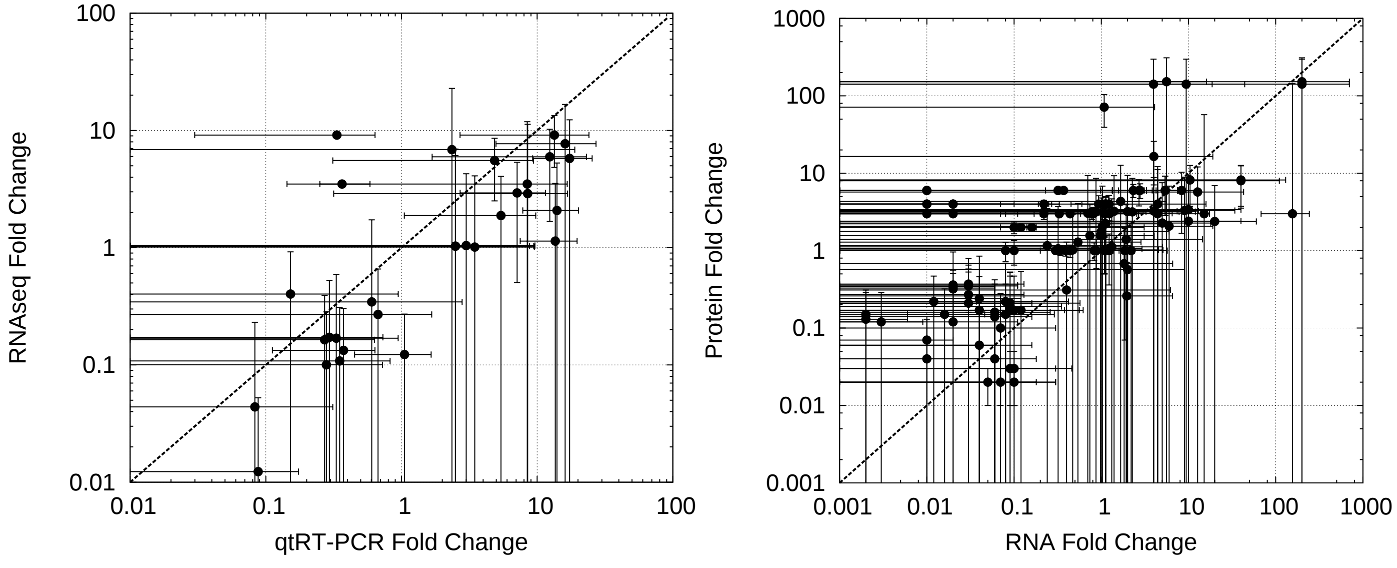

(a) (b)

Figure. S6 Comparison of RNAseq data to previous experiments. The line indicates the exact diagonal. (A) A comparison of fold change between conditions computed from our RNAseq data of this study to qtRT-PCR or Microarray data from previous studies shows a linear relationship with a slope of 0.96 and an overall correlation of 0.82. (B) A comparison of fold change from our RNAseq data to fold change in reported protein abundances demonstrates a correlation of 0.63.

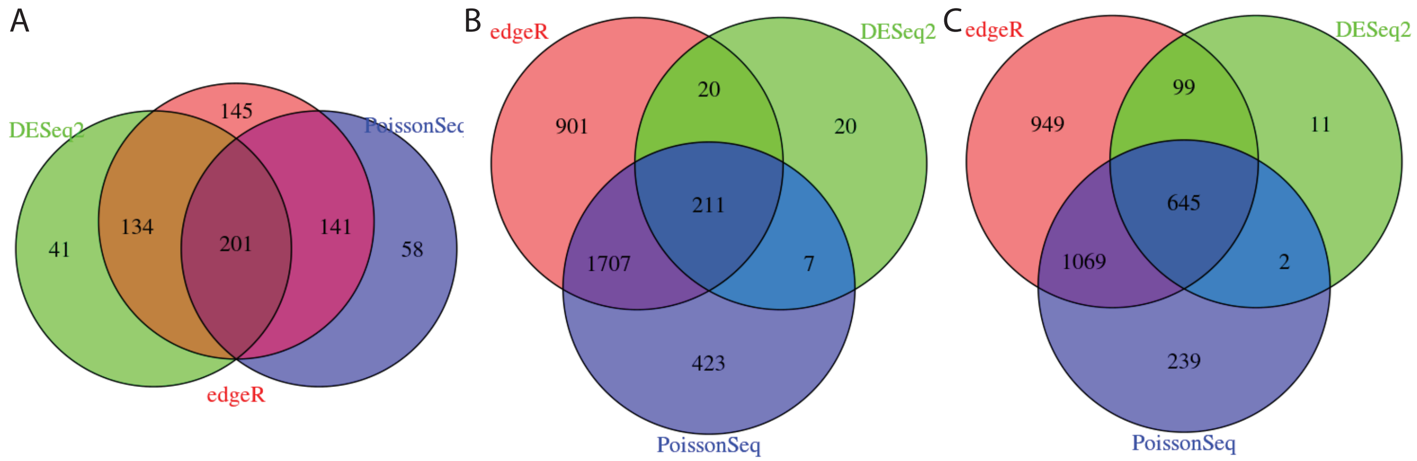

Figure. S7 Count of differentially expressed genes where p ≤ 0.01 predicted by each method when comparing: (A) Methanol vs. Acetate, (B) Methanol vs. TMA, and (C) TMA vs. Acetate. In general DESeq2 is the most conservative method. The overlap drastically reduces the number of DEG and provides a more certain set of predictions.

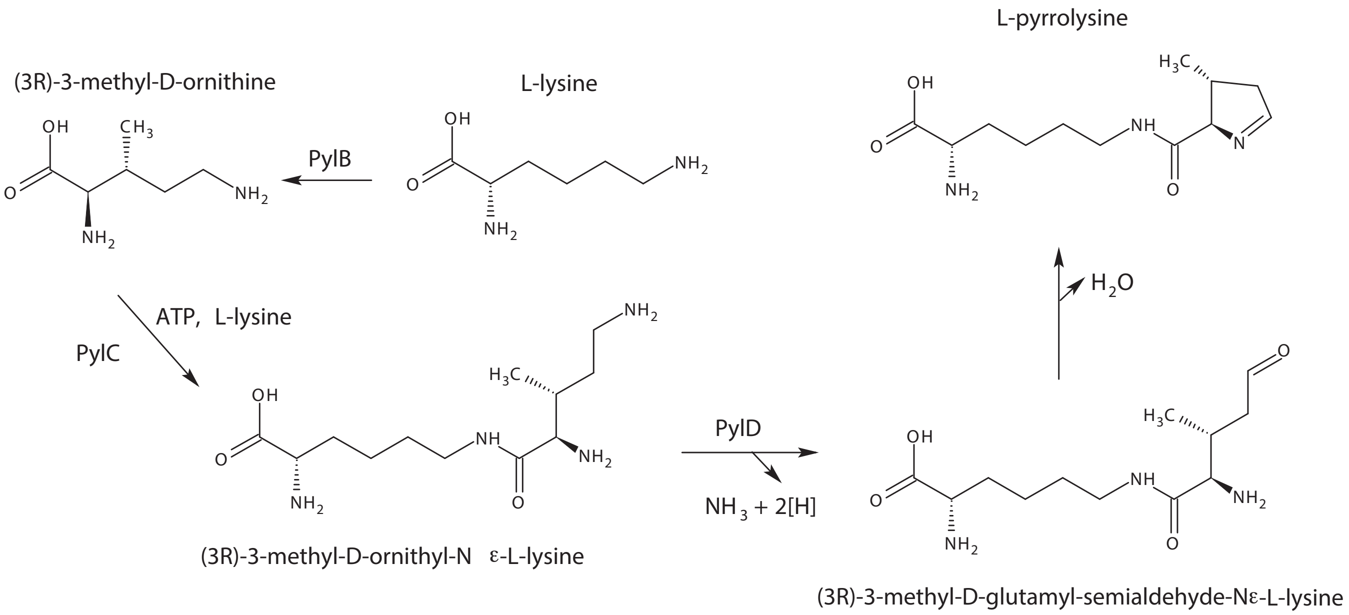

Figure. S8 Pyrrolysine biosynthesis pathway

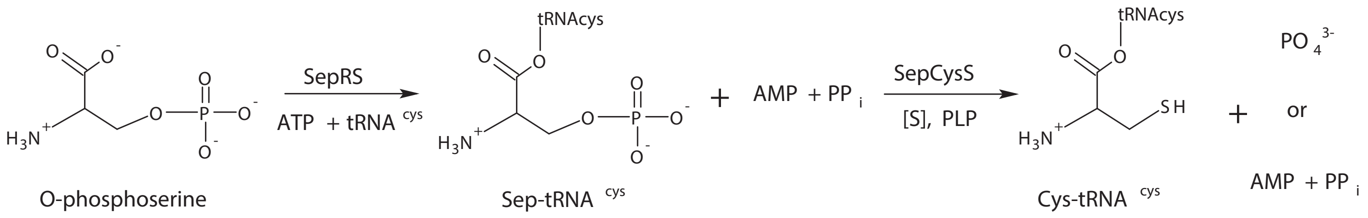

Figure. S9 Alternate cysteine aminoacylation pathway involving SepRS and SepCysS enzymes.

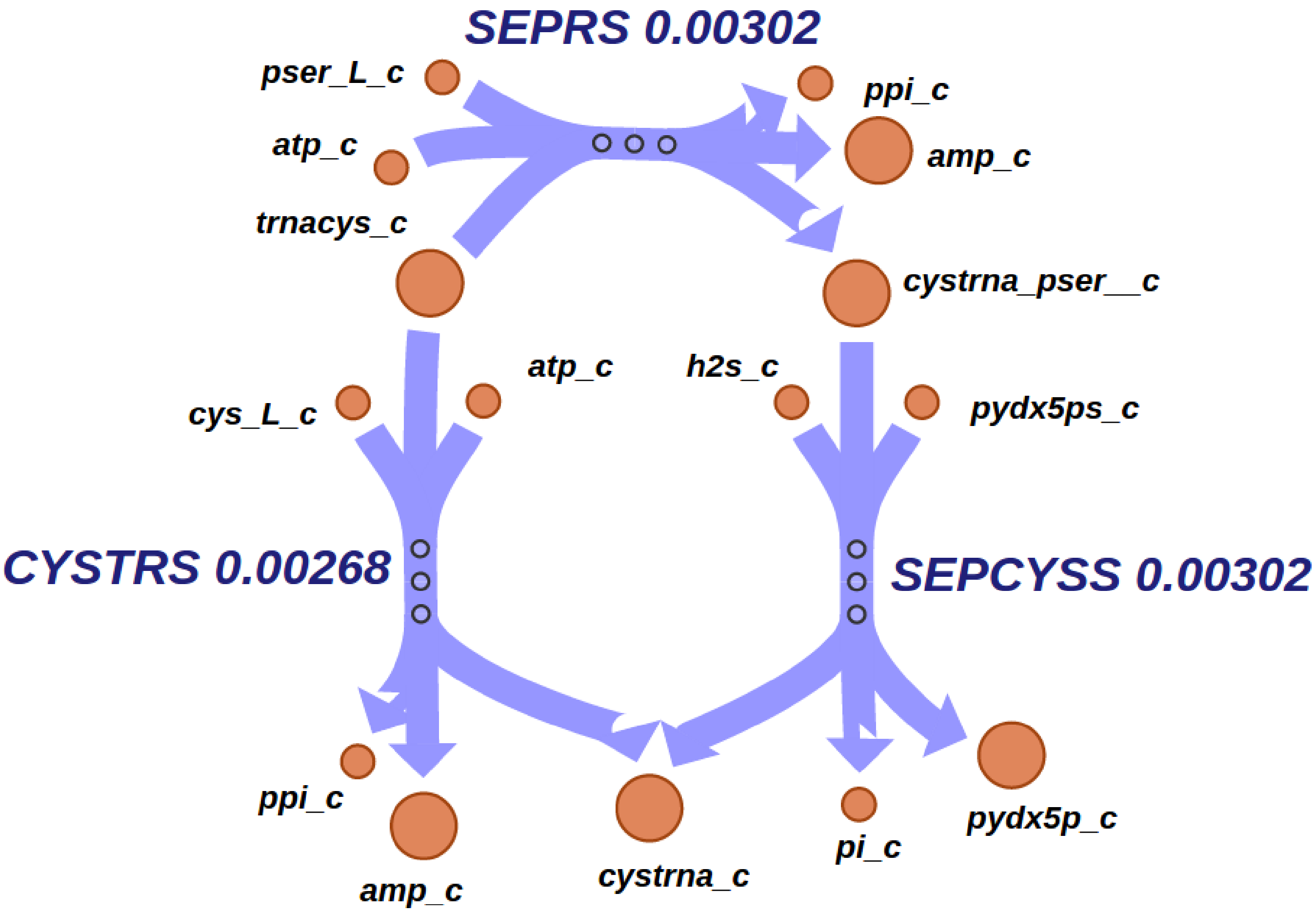

Figure. S10 Flux distribution of CysRS and SepRS under MeOH growth

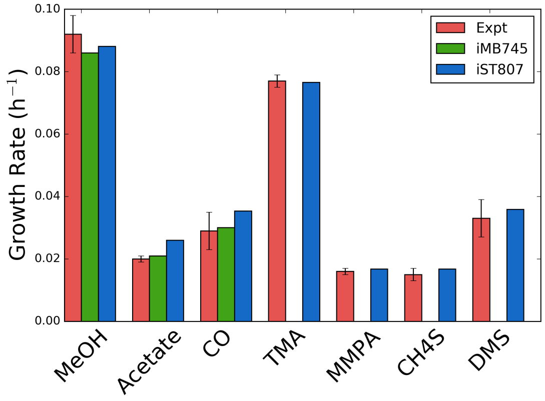

(a) Growth rates

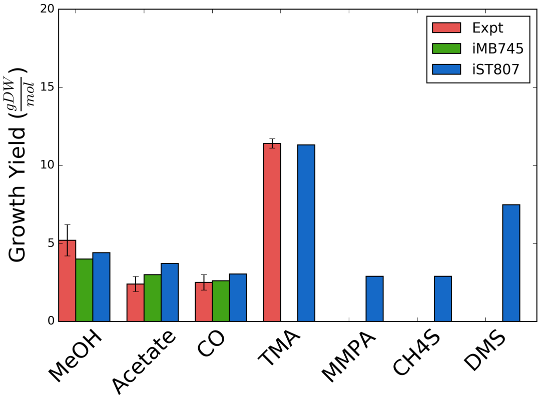

(b) Growth yields

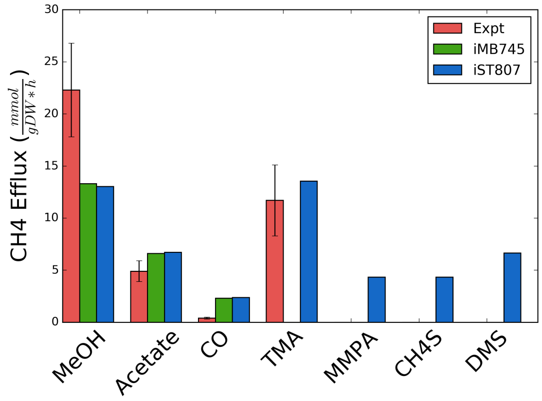

(c) CH_4 production

Figure. S11 Model predictions compared with experimental data. A) Growth rates (hr^{-1}) [97, 41, 95, 113, 43, 57, 114, 115, 96, 27, 74]. B) Growth yields (gDW/mol substrate) [97, 116, 117]. Note that experimental TMA growth yield was computed from TMA growth rate and a fitted TMA uptake rate as there were no experimental uptake values available. C) CH_4 production rate (mmol/hr/gDW) [43, 118, 117, 119]

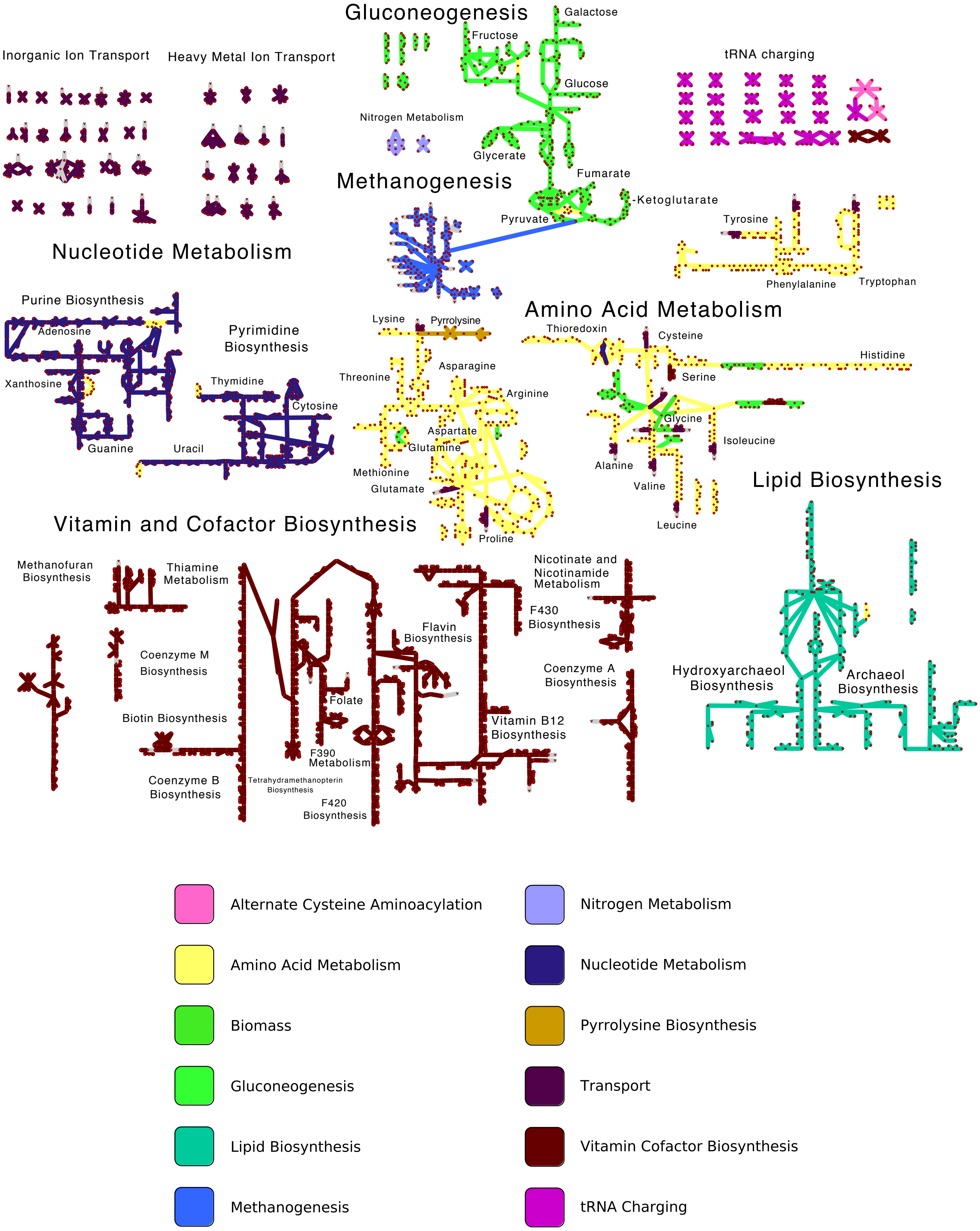

Figure. S12 Metabolic map of the updated *M. acetivorans* model published with this paper. Red dots denote reactions and metabolites while edges describe the connection of metabolites through reactions. In general, reactions are drawn such that metabolic flux flows down and to the right; however, there are many exceptions notably in gluconeogenesis and cofactor/lipid metabolism. Reactions are colored by metabolic classification. This map is available in format in formats compatible with Cytoscape [64] and Escher [65] in the Supporting File “ModelAndMaps.zip".

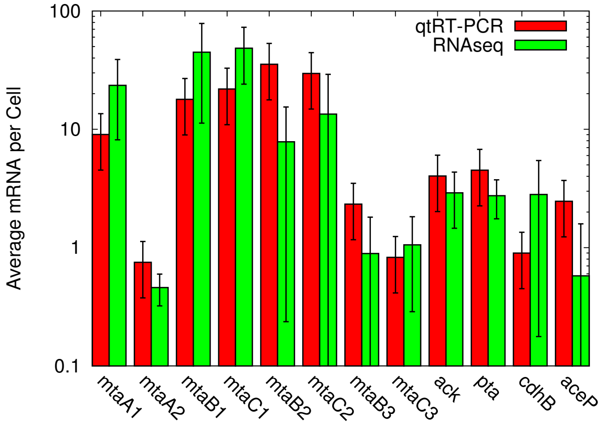

(a)

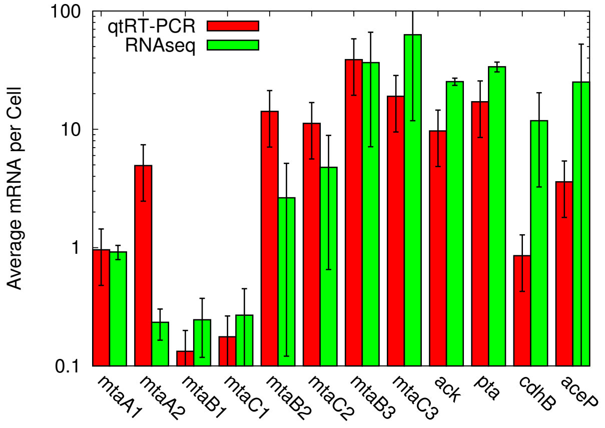

(b)

Figure. S13 A comparison of mRNA copies per cell estimated via our RNAseq data, and previous studies that utilized RT-qPCR to quantify transcript abundance in the related organism *Methanosarcina mazei* [67] grown in (A) methanol and (B) acetate. Error bars are standard deviation of the mean for 3 replicates. Values from Cao et al. are for cells grown at 30°C compared to our cells which were grown at 37°C. All values agree within uncertanties except for *cdh*, *mtaA2*, and *mtaB2* indicating the organisms have similar expression profiles and our estimates for mRNA counts are good.

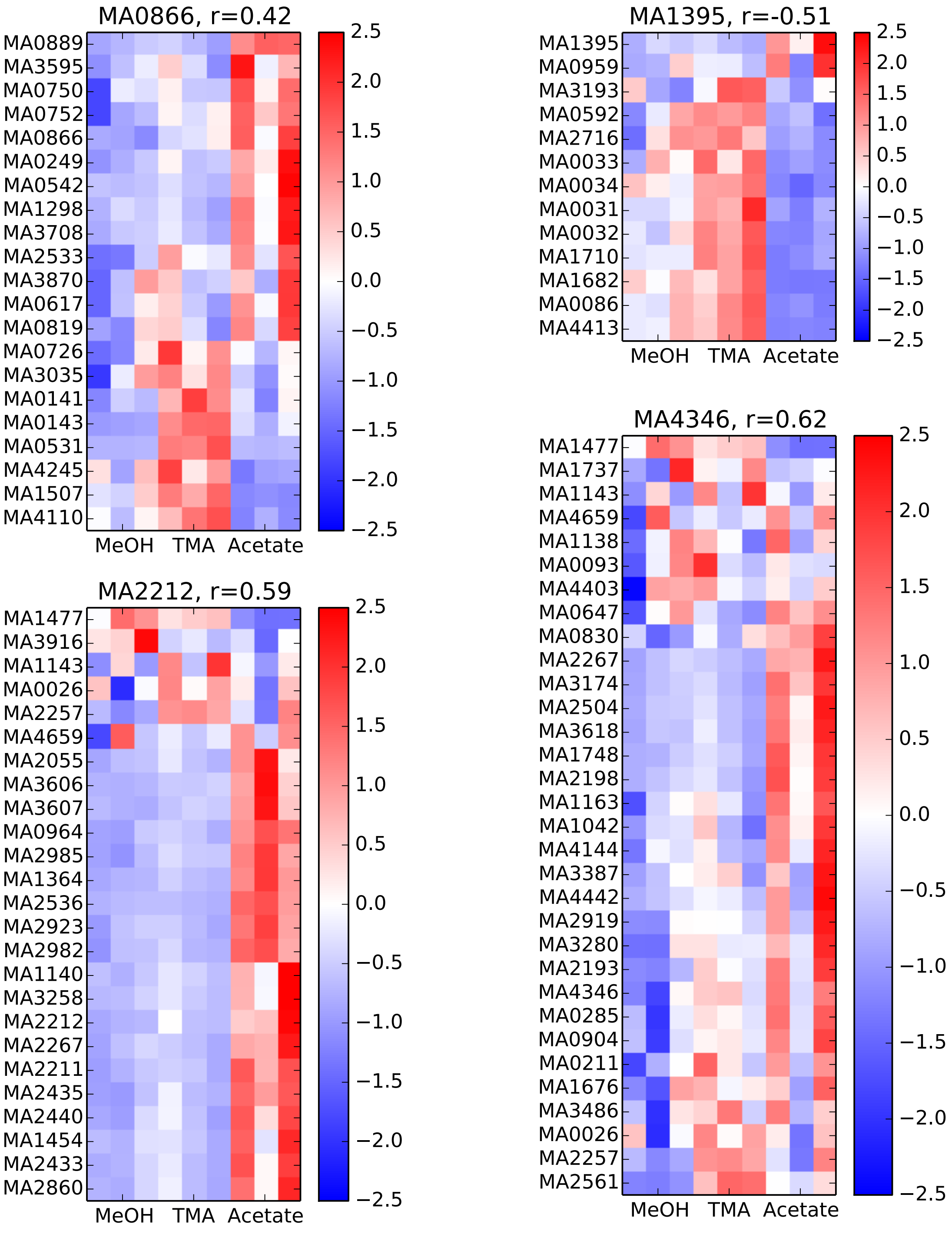

Figure. S14 Heatmaps of relative expression for 4 putative regulators that are differentially expressed between at least one pair of conditions. Each regulator is highly conserved among the *Methanosarinales*. *MA1395* is highly conserved among most methanogens and encodes for a nickel response regulator. The regulator is indicated in the title of each heatmap along with the correlation of the regulator's expression to the other genes that have the same conservation pattern.

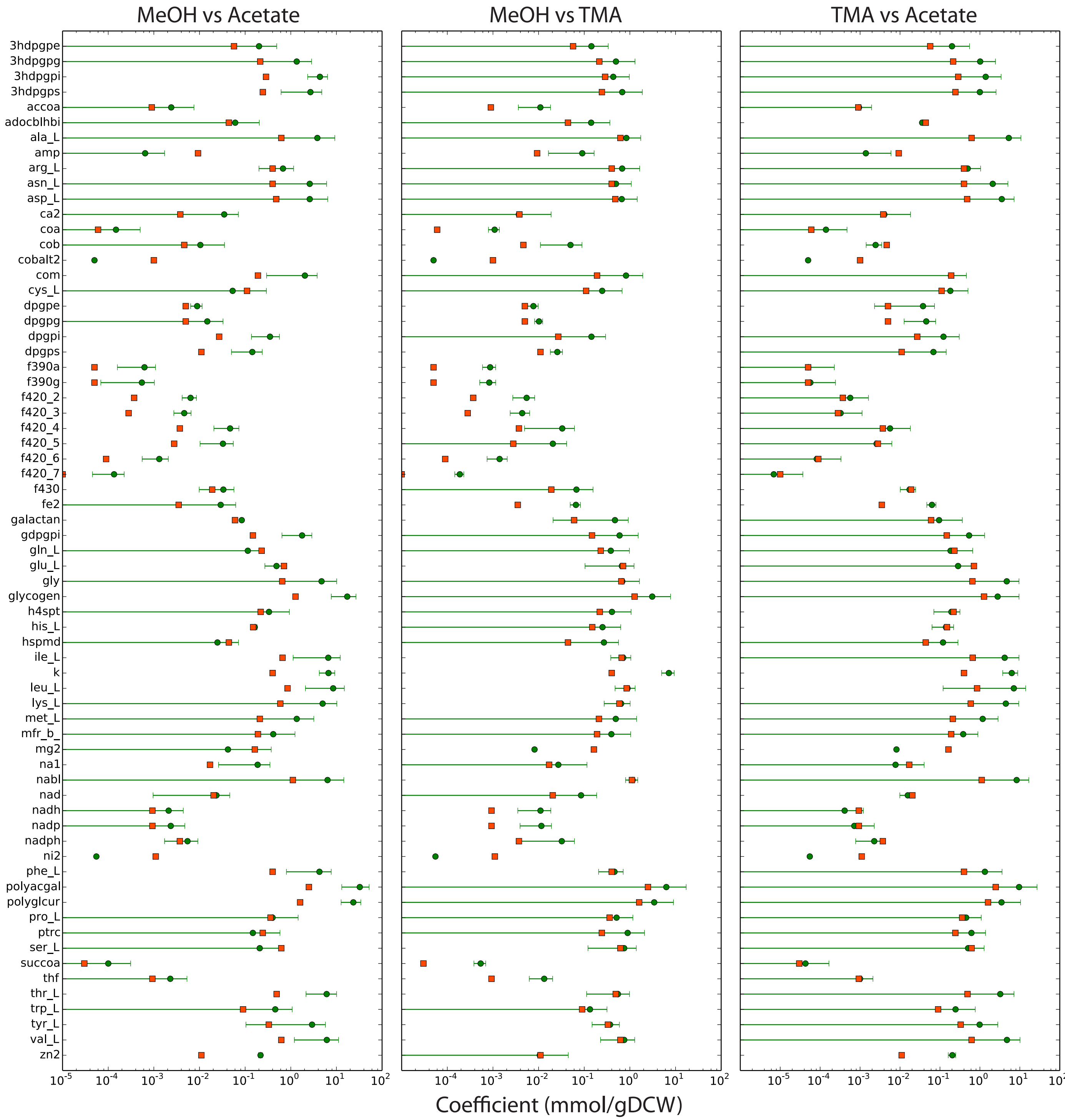

Figure. S15 Biomass coefficients after fitting metabolic flux distributions to (green circles) compared with biomass coefficients published with the original model (orange squares). In each case, the substrate listed first was taken to be the reference, and the differentially expressed genes going to the second substrate were used to fit the flux distributions and subsequently the biomass composition (see SI Section 1.3.2). Results shown for: (a) Methanol vs. Acetate, (b) Methanol vs. TMA, and (c) TMA vs. Acetate.

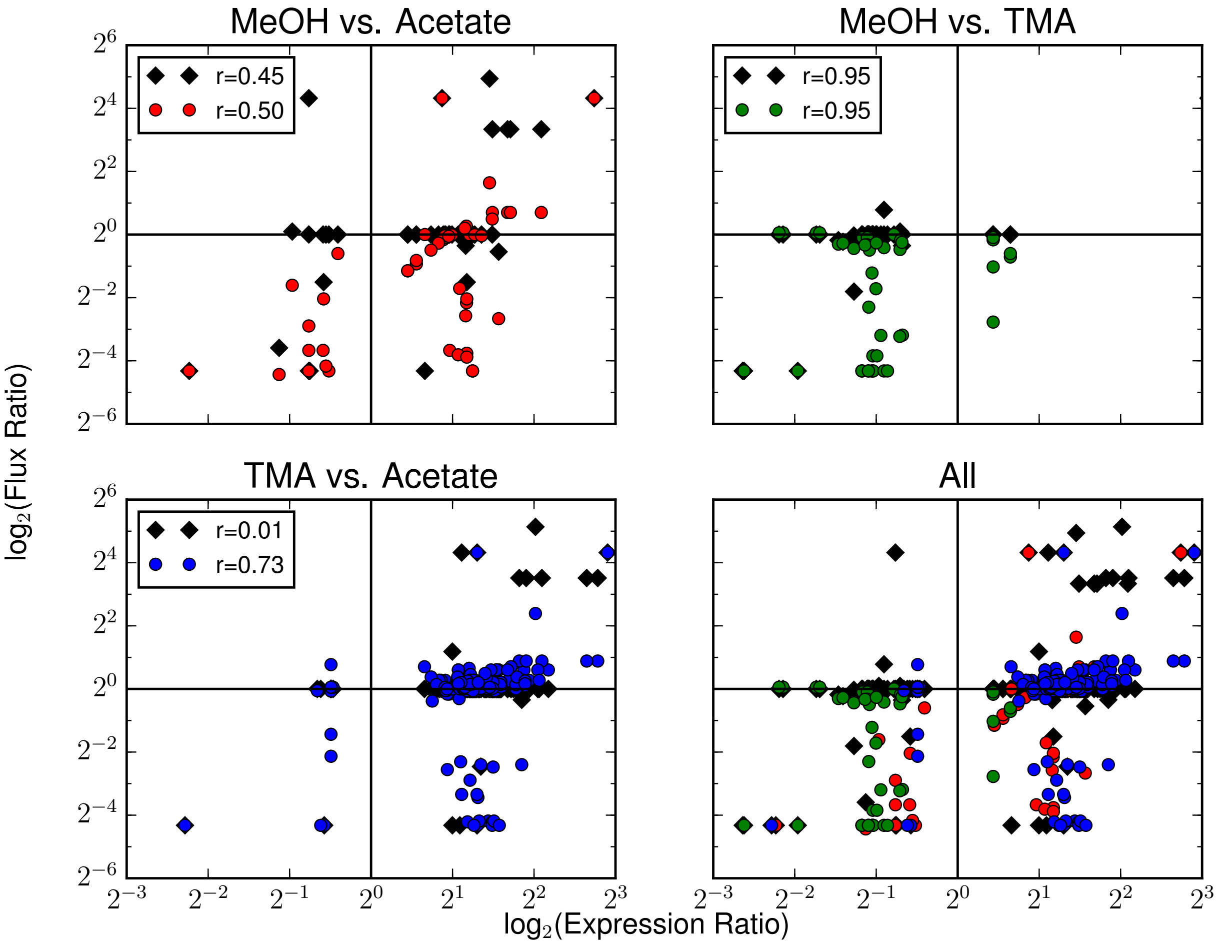

Figure. S16 A comparison of the ratio of fluxes of the first to second substrate computed over the whole metabolic model, to the ratio of DEG for that reaction. Predictions pre-fitting are shown as black diamonds while values after fitting are shown as circles. Overall, correlation to the experiment increases from r=0.33 to r=0.51.

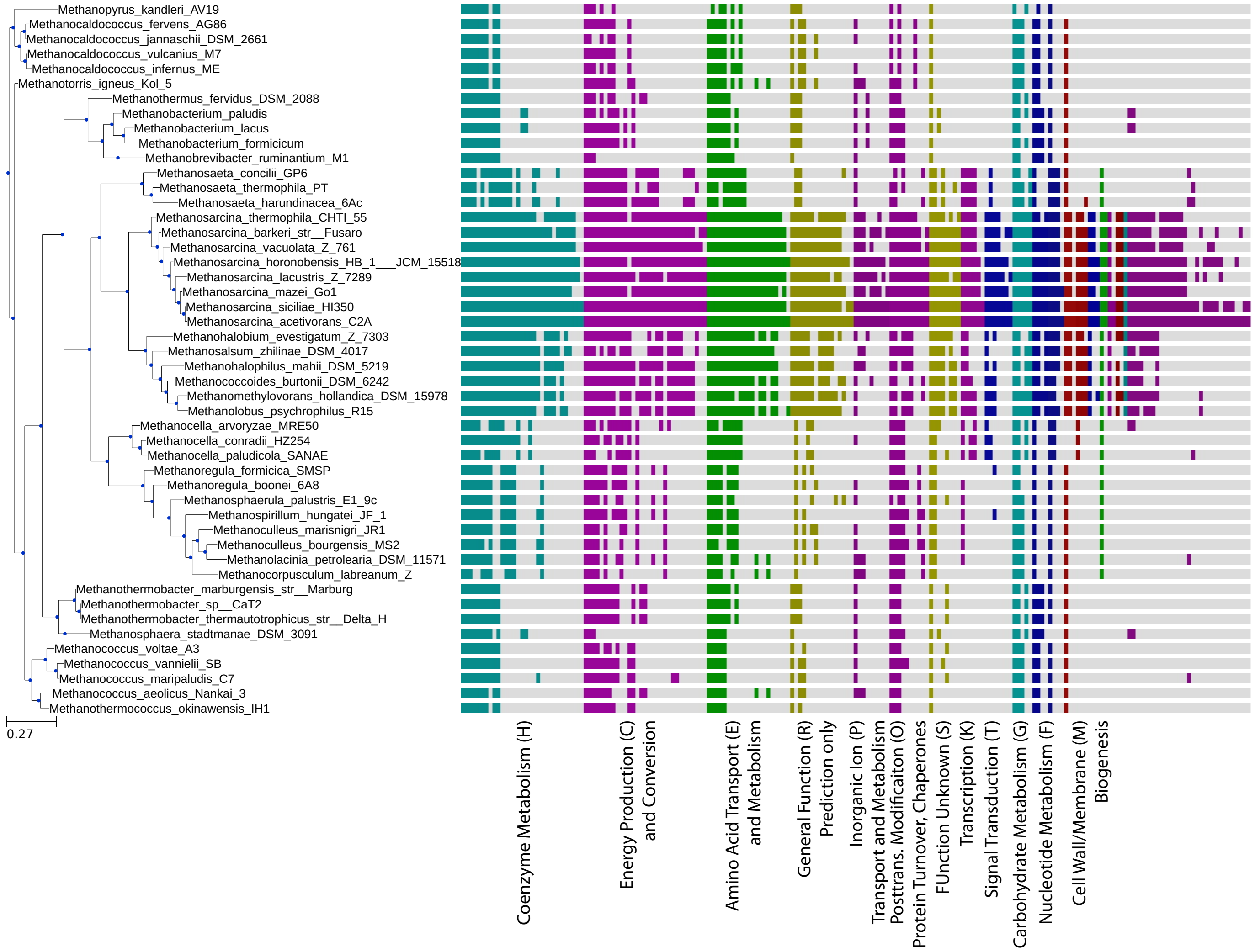

Figure. S17 Conservation of the genes that are differentially expressed between MeOH and TMA across the tree of methanogens. Each vertical bar indicates that a homolog for the differentially expressed gene exists in the indicated species (computed as the bidirectional best hits functionality in the ITEP software [92] with an E-value cut-off of 10^{-5} for a database of $\sim$125000 proteins). Most differentially expressed genes are highly conserved among the *Methanosarcinales*; however a core set of genes are conserved across all methanogens.

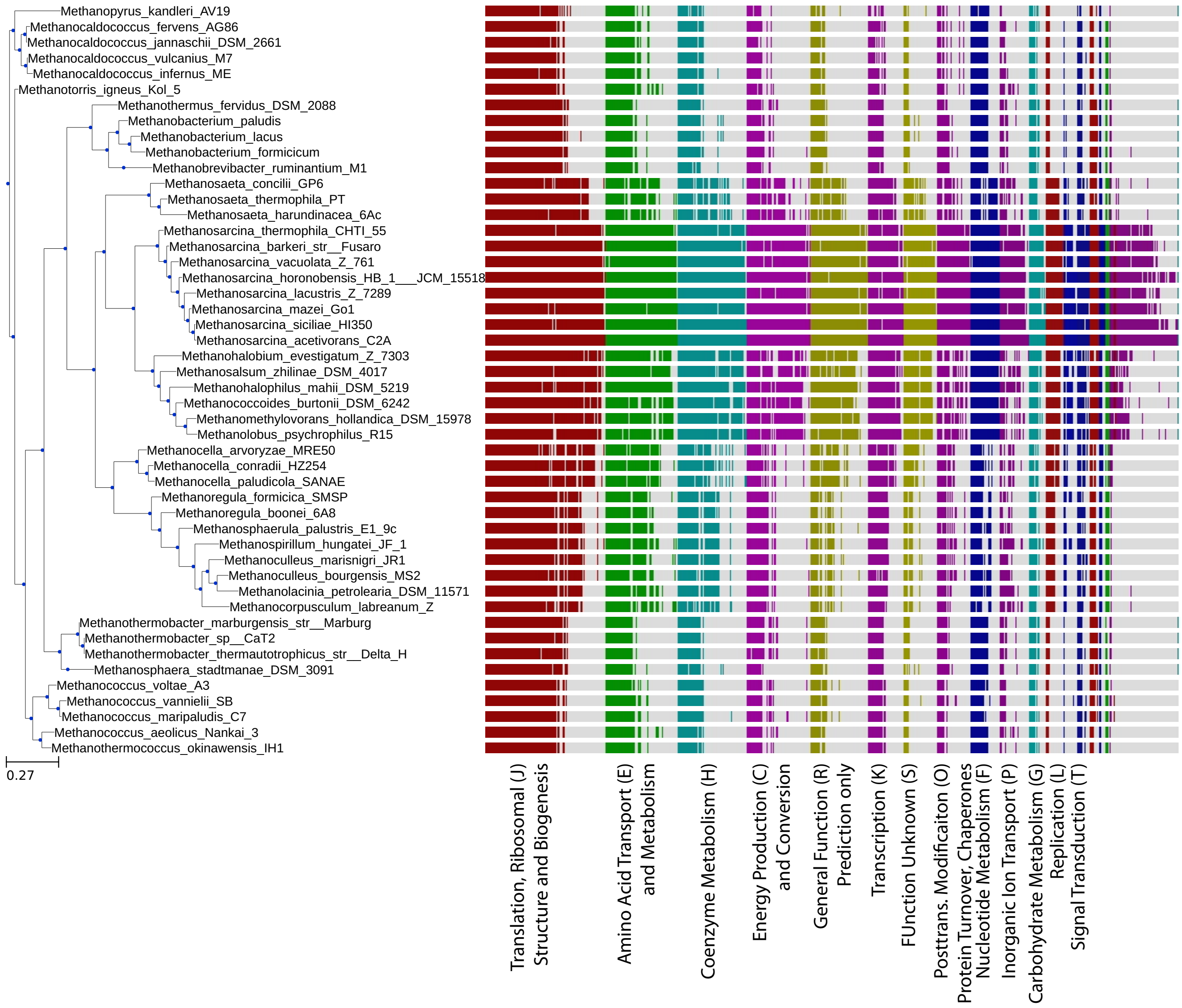

Figure. S18 Conservation of the genes that are differentially expressed between TMA and Acetate across the tree of methanogens. Each vertical bar indicates that a homolog for the differentially expressed gene exists in the indicated species (computed as the bidirectional best hits functionality in the ITEP software [92] with an E-value cut-off of 10^{-5} for a database of $\sim$125000 proteins). Most differentially expressed genes are highly conserved among the *Methanosarcinales*; however a core set of genes are conserved across all methanogens.

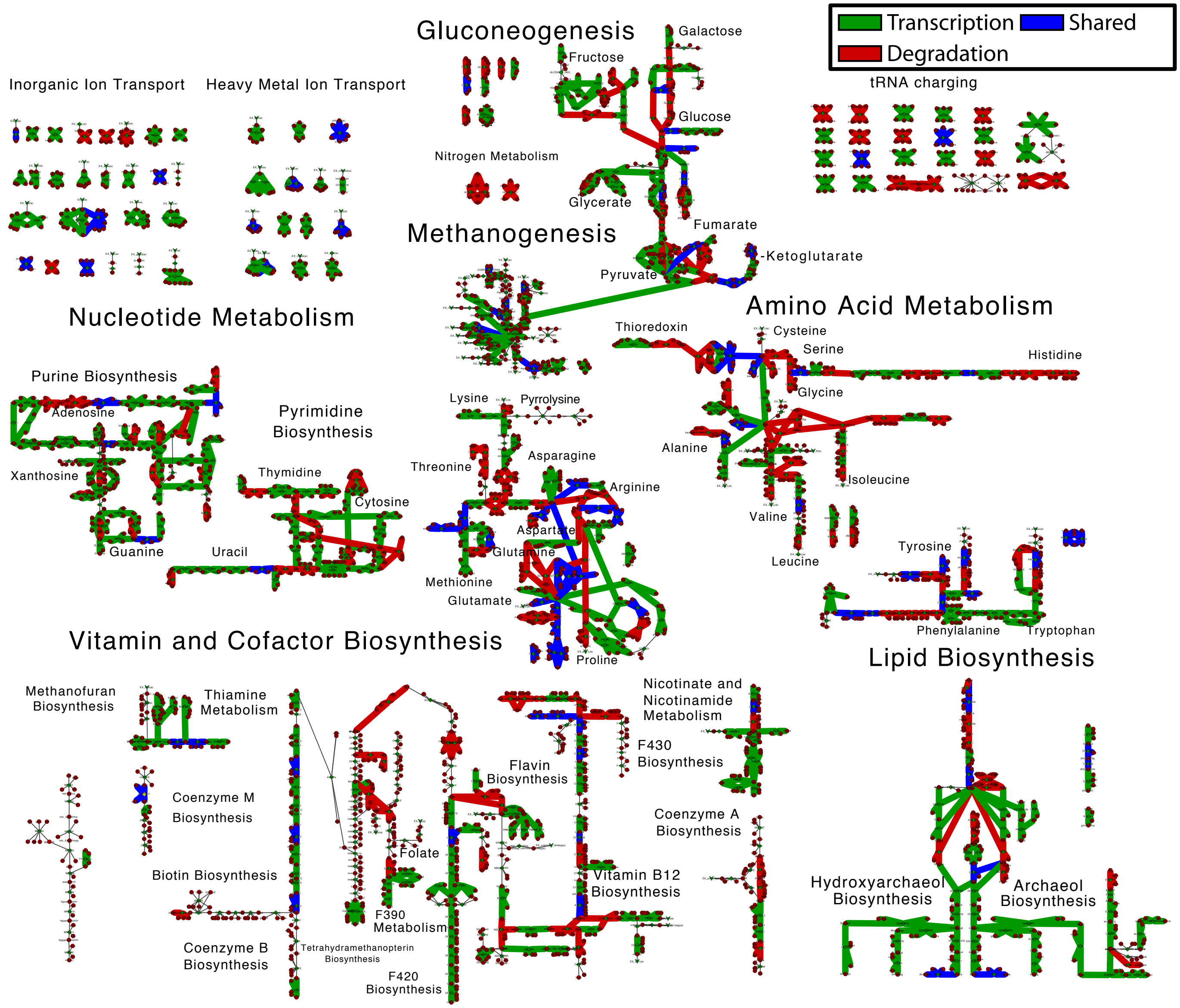

Figure. S19 A mapping of the control coefficients for changing mRNA expression levels between MeOH and TMA. Red indicate reactions where mRNA levels are regulated by shifts in the degradation rate, while green indicates mRNA level shifts due to changes in transcription rate. Blue indicates reactions where mRNA levels are affected by both transcription and degradation rate.

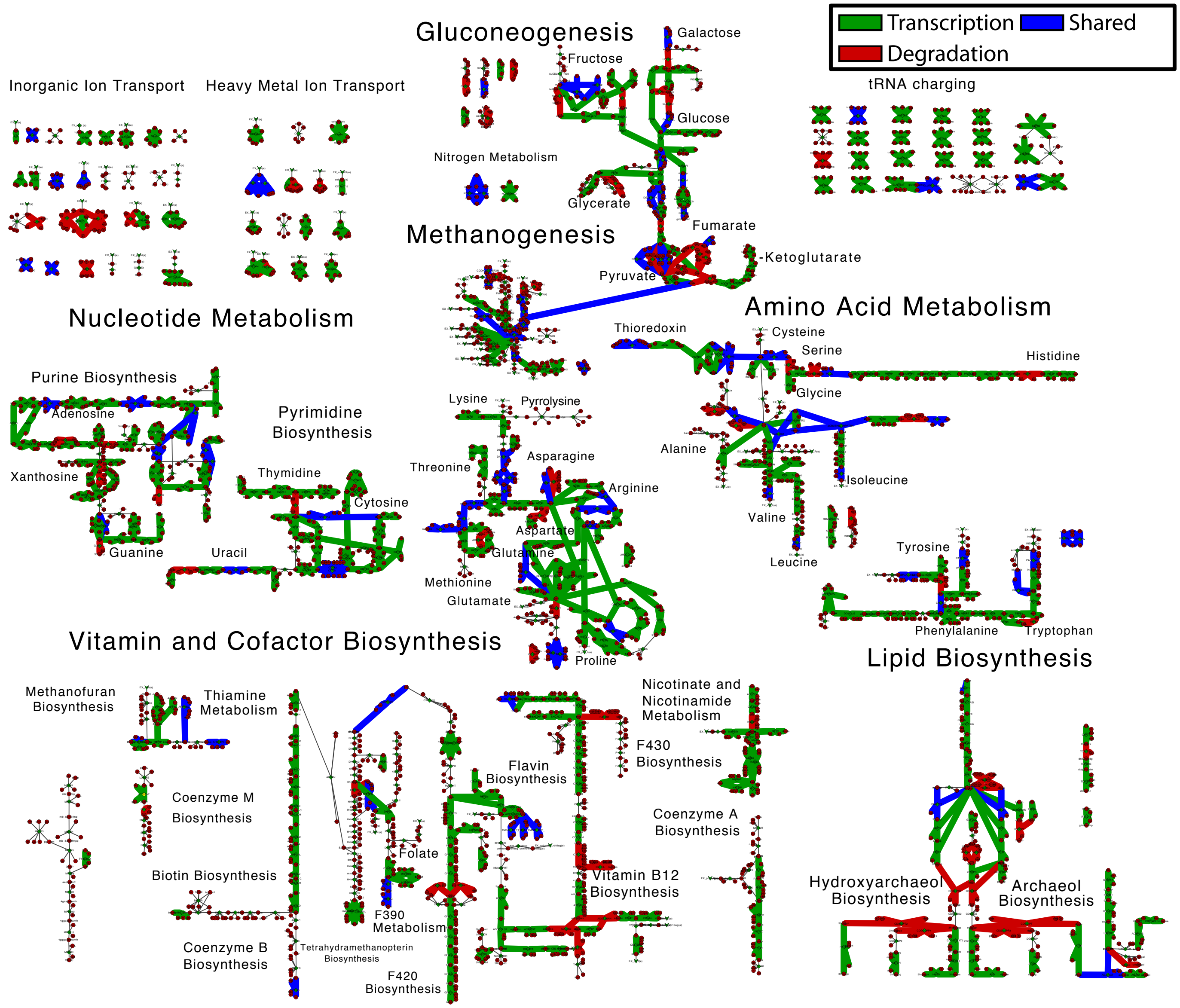

Figure. S20 A mapping of the control coefficients for changing mRNA expression levels between TMA and acetate. Red indicate reactions where mRNA levels are regulated by shifts in the degradation rate, while green indicates mRNA level shifts due to changes in transcription rate. Blue indicates reactions where mRNA levels are affected by both transcription and degradation rate.

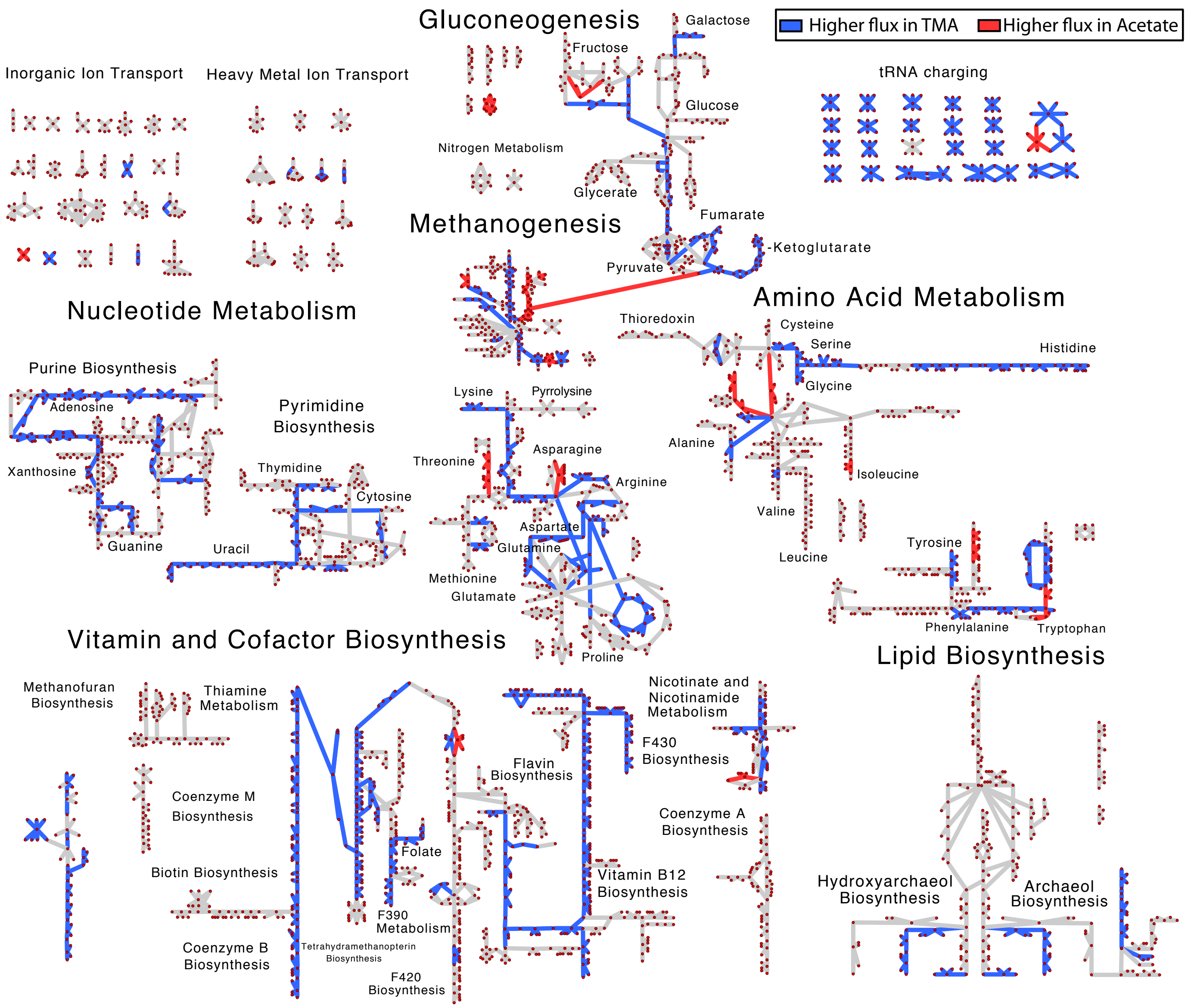

Figure. S21 Changes in metabolic pathway usage that is consistent with the differentially expressed genes comparing MeOH to acetate growth. Pathways with significant changes in fluxes are shown in red (up in acetate) and cyan (down in acetate) while reactions showing no change in flux (change in flux <2x) or having no associated genes in gray. Significant metabolic changes are observed across nearly all of metabolism, however no changes are are predicted for coenzyme A and M biosynthesis, thiamine metabolism, or leucine/isoleucine/methionine synthesis. Additionally, only changes in phosphoethanolamine and phosphoglycerol based lipids.

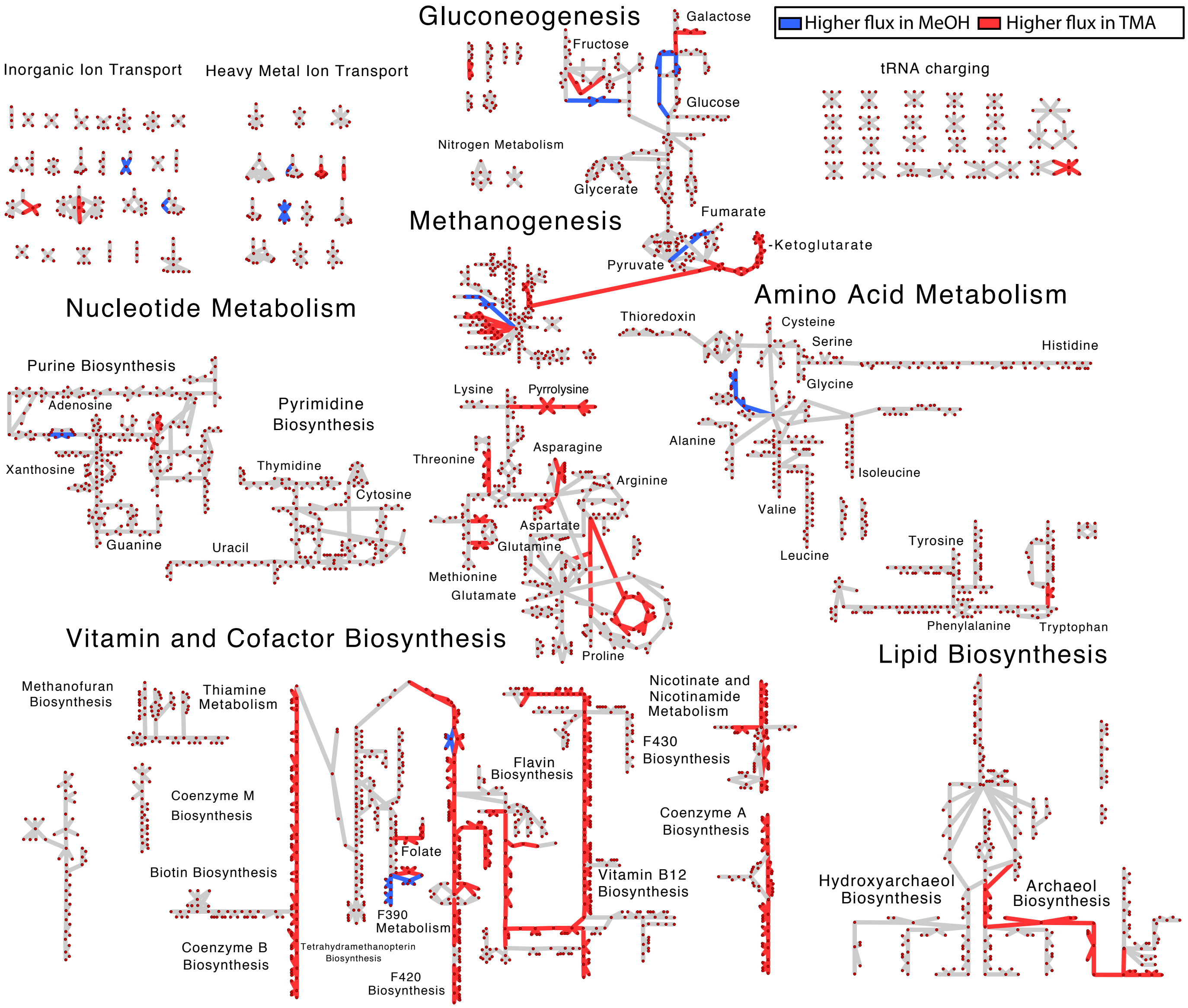

Figure. S22 Changes in metabolic pathway usage that is consistent with the differentially expressed genes comparing MeOH to TMA growth. Pathways with significant changes in fluxes are shown in red (up in TMA) and cyan (down in TMA) while reactions showing no change in flux (change in flux <2x) or having no associated genes in gray. Cofactor and vitamin metabolism show the most significant changes, along with central amino acid metabolism (including production of α-ketoglutarate and malate) and the pathway producing glucosaminyl archaetidyl-myo-inositol lipids.

References

[1] Fei, J., Singh, D., Zhang, Q., Park, S., Balasubramanian, D., Golding, I., Vanderpool, C.K., Ha, T.: Determination of in vivo target search kinetics of regulatory noncoding RNA. Science **347**(6228), 1371–1374 (2015). doi:10.1126/science.1258849

[2] Rogowska, A.T., Puchta, O., Czarnecka, A.M., Kaniak, A., Stepien, P.P., Golik, P.: Balance between Transcription and RNA Degradation Is Vital for *Saccharomyces cerevisiae* Mitochondria: Reduced Transcription Rescues the Phenotype of Deficient RNA Degradation. Mol. Biol. Cell **17**(3), 1184–1193 (2005). doi:10.1091/mbc.e05-08-0796

[3] Chiba, Y., Mineta, K., Hirai, M.Y., Suzuki, Y., Kanaya, S., Takahashi, H., Onouchi, H., Yamaguchi, J., Naito, S.: Changes in mRNA Stability Associated with Cold Stress in Arabidopsis Cells. Plant Cell Physiol. **54**(2), 180–194 (2012). doi:10.1093/pcp/pcs164

[4] Peterson, J.R., Cole, J.A., Fei, J., Ha, T., Luthey-Schulten, Z.A.: Effects of DNA replication on mRNA noise. Proc. Natl. Acad. Sci. U.S.A. **112**(52), 15886–15891 (2015). doi:10.1073/pnas.1516246112

[5] Bernstein, J.A., Khodursky, A.B., Lin, P.H., Lin-Chao, S., Cohen, S.N.: Global analysis of mRNA decay and abundance in *Escherichia coli* at single-gene resolution using two-color fluorescent DNA microarrays. Proc. Natl. Acad. Sci. U.S.A. **99**(15), 9697–9702 (2002)

[6] Selinger, D.W., Saxena, R.M., Cheung, K.J., Church, G.M., Rosenow, C.: Global RNA half-life analysis in *Escherichia coli* reveals positional patterns of transcript degradation. Genome Res. **13**(2), 216–223 (2003)

[7] Esquerré, T., Laguerre, S., Turlan, C., Carpousis, A.J., Girbal, L., Cocaign-Bousquet, M.: Dual role of transcription and transcript stability in the regulation of gene expression in *Escherichia coli* cells cultured on glucose at different growth rates. Nucleic Acids Res. **42**(4), 2460–2472 (2013). doi:10.1093/nar/gkt1150

[8] Dressaire, C., Picard, F., Redon, E., Loubière, P., Queinnec, I., Girbal, L., Cocaign-Bousquet, M.: Role of mRNA Stability during Bacterial Adaptation. PLoS ONE **8**(3), 59059 (2013). doi:10.1371/journal.pone.0059059

[9] Esquerré, T., Moisan, A., Chiapello, H., Arike, L., Vilu, R., Gaspin, C., Cocaign-Bousquet, M., Girbal, L.: Genome-wide investigation of mRNA lifetime determinants in *Escherichia coli* cells cultured at different growth rates. BMC Genomics **16**(1) (2015). doi:10.1186/s12864-015-1482-8

[10] Rustad, T.R., Minch, K.J., Brabant, W., Winkler, J.K., Reiss, D.J., Baliga, N.S., Sherman, D.R.: Global analysis of mRNA stability in *Mycobacterium tuberculosis*. Nucleic Acids Res. **41**(1), 509–517 (2012). doi:10.1093/nar/gks1019

[11] Hambraeus, G., von Wachenfeldt, C., Hederstedt, L.: Genome-wide survey of mRNA half-lives in *Bacillus subtilis* identifies extremely stable mRNAs. Mol. Genet. Genomics **269**(5), 706–714 (2003). doi:10.1007/s00438-003-0883-6

[12] Kristoffersen, S.M., Haase, C., Weil, M.R., Passalacqua, K.D., Niazi, F., Hutchison, S.K., Desany, B., Kolstø, A.B., Tourasse, N.J., Read, T.D., Økstad, O.: Global mRNA decay analysis at single nucleotide resolution reveals segmental and positional degradation patterns in a gram-positive bacterium. Genome Biol **13**(4), 30 (2012). doi:10.1186/gb-2012-13-4-r30

[13] Bini, E., Dikshit, V., Dirksen, K., Drozda, M., Blum, P.: Stability of mRNA in the hyperthermophilic archaeon *Sulfolobus solfataricus*. RNA **8**(9), 1129–1136 (2002)

[14] Andersson, A.F., Lundgren, M., Eriksson, S., Rosenlund, M., Bernander, R., Nilsson, P.: Global analysis of mRNA stability in the archaeon *Sulfolobus*. Genome Biol. **7**(10), 99 (2006). doi:10.1186/gb-2006-7-10-r99

[15] Hundt, S., Zaigler, A., Lange, C., Soppa, J., Klug, G.: Global Analysis of mRNA Decay in *Halobacterium salinarum* NRC-1 at Single-Gene Resolution Using DNA Microarrays. J. Bacteriol. **189**(19), 6936–6944 (2007). doi:10.1128/jb.00559-07

[16] Bult, C.J., White, O., Olsen, G.J., Zhou, L., Fleischmann, R.D., Sutton, G.G., Blake, J.A., FitzGerald, L.M., Clayton, R.A., Gocayne, J.D., Kerlavage, A.R., Dougherty, B.A., Tomb, J.-F., Adams, M.D., Reich, C.I., Overbeek, R., Kirkness, E.F., Weinstock, K.G., Merrick, J.M., Glodek, A., Scott, J.L., Geoghagen, N.S.M., Weidman, J.F., Fuhrmann, J.L., Nguyen, D., Utterback, T.R., Kelley, J.M., Peterson, J.D., Sadow, P.W., Hanna, M.C., Cotton, M.D., Roberts, K.M., Hurst, M.A., Kaine, B.P., Borodovsky, M., Klenk, H.-P., Fraser, C.M., Smith, H.O., Woese, C.R., Venter, J.C.: Complete genome sequence of the methanogenic archaeon, methanococcus jannaschii. Science **273**(5278), 1058–1073 (1996). doi:10.1126/science.273.5278.1058

[17] Zhang, J., Olsen, G.J.: Messenger RNA processing in *Methanocaldococcus (Methanococcus) jannaschii*. RNA **15**(10), 1909–1916 (2009)

[18] Wang, Y., Liu, C.L., Storey, J.D., Tibshirani, R.J., Herschlag, D., Brown, P.O.: Precision and functional specificity in mRNA decay. Proc. Natl. Acad. Sci. U.S.A. **99**(9), 5860–5865 (2002)

[19] Grigull, J., Mnaimneh, S., Pootoolal, J., Robinson, M.D., Hughes, T.R.: Genome-Wide Analysis of mRNA Stability Using Transcription Inhibitors and Microarrays Reveals Posttranscriptional Control of Ribosome Biogenesis Factors. Mol. Cell. Biol. **24**(12), 5534–5547 (2004). doi:10.1128/mcb.24.12.5534-5547.2004

[20] Munchel, S.E., Shultzaberger, R.K., Takizawa, N., Weis, K.: Dynamic profiling of mRNA turnover reveals gene-specific and system-wide regulation of mRNA decay. Molecular Biology of the Cell **22**(15), 2787–2795 (2011). doi:10.1091/mbc.e11-01-0028

[21] Geisberg, J.V., Moqtaderi, Z., Fan, X., Ozsolak, F., Struhl, K.: Global Analysis of mRNA Isoform Half-Lives Reveals Stabilizing and Destabilizing Elements in Yeast. Cell **156**(4), 812–824 (2014). doi:10.1016/j.cell.2013.12.026

[22] Galagan, J.E., Nusbaum, C., Roy, A., Endrizzi, M.G., Macdonald, P., FitzHugh, W., Calvo, S., Engels, R., Smirnov, S., Atnoor, D., Brown, A., Allen, N., Naylor, J., Stange-Thomann, N., DeArellano, K., Johnson, R., Linton, L., McEwan, P., McKernan, K., Talamas, J., Tirrell, A., Ye, W., Zimmer, A., Barber, R.D., Cann, I., Graham, D.E., Grahame, D.A., Guss, A.M., Hedderich, R., Ingram-Smith, C., Kuettner, H.C., Krzycki, J.A., Leigh, J.A., Li, W., Liu, J., Mukhopadhyay, B., Reeve, J.N., Smith, K., Springer, T.A., Umayam, L.A., White, O., White, R.H., Conway de Macario, E., Ferry, J.G., Jarrell, K.F., Jing, H., Macario, A.J., Paulsen, I., Pritchett, M., Sowers, K.R., Swanson, R.V., Zinder, S.H., Lander, E., Metcalf, W.W., Birren, B.: The genome of *M. acetivorans* reveals extensive metabolic and physiological diversity. Genome Res. **12**(4), 532–542 (2002)

[23] Thauer, R.K., Kaster, A.-K., Seedorf, H., Buckel, W., Hedderich, R.: Methanogenic archaea: ecologically relevant differences in energy conservation. Nat. Rev. Micro. **6**(8), 579–591 (2008). doi:10.1038/nrmicro1931

[24] Rothman, D.H., Fournier, G.P., French, K.L., Alm, E.J., Boyle, E.A., Cao, C., Summons, R.E.: Methanogenic burst in the end-Permian carbon cycle. Proc. Natl. Acad. Sci. U.S.A. **111**(15), 5462–5467 (2014). doi:10.1073/pnas.1318106111

[25] Bapteste, E., Brochier, C., Boucher, Y.: Higher-level classification of the Archaea: evolution of methanogenesis and methanogens. Archaea **1**(5), 353–363 (2005)

[26] Maslov, S., Krishna, S., Pang, T.Y., Sneppen, K.: Toolbox model of evolution of prokaryotic metabolic networks and their regulation. Proc. Natl. Acad. Sci. U.S.A. **106**(24), 9743–9748 (2009). doi:10.1073/pnas.0903206106

[27] Matschiavelli, N., Oelgeschlager, E., Cocchiararo, B., Finke, J., Rother, M.: Function and Regulation of Isoforms of Carbon Monoxide Dehydrogenase/Acetyl Coenzyme A Synthase in *Methanosarcina acetivorans*. J. Bacteriol. **194**(19), 5377–5387 (2012). doi:10.1128/jb.00881-12

[28] Li, J., Qi, L., Guo, Y., Yue, L., Li, Y., Ge, W., Wu, J., Shi, W., Dong, X.: Global mapping transcriptional start sites revealed both transcriptional and post-transcriptional regulation of cold adaptation in the methanogenic archaeon *Methanolobus psychrophilus*. Sci Rep **5**, 9209 (2015)

[29] Jager, D., Sharma, C.M., Thomsen, J., Ehlers, C., Vogel, J., Schmitz, R.A.: Deep sequencing analysis of the *Methanosarcina mazei* Go1 transcriptome in response to nitrogen availability. Proc. Natl. Acad. Sci. U.S.A. **106**(51), 21878–21882 (2009). doi:10.1073/pnas.0909051106

[30] Jager, D., Pernitzsch, S.R., Richter, A.S., Backofen, R., Sharma, C.M., Schmitz, R.A.: An archaeal sRNA targeting cis- and trans-encoded mRNAs via two distinct domains. Nucleic Acids Research **40**(21), 10964–10979 (2012). doi:10.1093/nar/gks847

[31] Galperin, M.Y., Makarova, K.S., Wolf, Y.I., Koonin, E.V.: Expanded microbial genome coverage and improved protein family annotation in the COG database. Nucleic Acids Res. **43**(D1), 261–269 (2014). doi:10.1093/nar/gku1223

[32] Makarova, K., Wolf, Y., Koonin, E.: Archaeal Clusters of Orthologous Genes (arCOGs): An Update and Application for Analysis of Shared Features between Thermococcales, Methanococcales, and Methanobacteriales. Life **5**(1), 818–840 (2015). doi:10.3390/life5010818

[33] Mi, H., Muruganujan, A., Thomas, P.D.: PANTHER in 2013: modeling the evolution of gene function, and other gene attributes, in the context of phylogenetic trees. Nucleic Acids Res. **41**(D1), 377–386 (2012). doi:10.1093/nar/gks1118

[34] Robinson, M.D., McCarthy, D.J., Smyth, G.K.: edgeR: a Bioconductor package for differential expression analysis of digital gene expression data. Bioinform. **26**(1), 139–140 (2010)

[35] Li, J., Witten, D.M., Johnstone, I.M., Tibshirani, R.: Normalization, testing, and false discovery rate estimation for RNA-sequencing data. Biostats. **13**(3), 523–538 (2011). doi:10.1093/biostatistics/kxr031

[36] Love, M.I., Huber, W., Anders, S.: Moderated estimation of fold change and dispersion for RNA-seq data with DESeq2. Genome Biol. **15**(12) (2014). doi:10.1186/s13059-014-0550-8

[37] Rohlin, L., Gunsalus, R.: Carbon-dependent control of electron transfer and central carbon pathway genes for methane biosynthesis in the Archaean, *Methanosarcina acetivorans* strain C2A. BMC Microbiol. **10**(1), 62 (2010). doi:10.1186/1471-2180-10-62

[38] Li, Q., Li, L., Rejtar, T., Karger, B.L., Ferry, J.G.: Proteome of *Methanosarcina acetivorans* Part II: Comparison of Protein Levels in Acetate- and Methanol-Grown Cells. J. Proteome Res. **4**(1), 129–135 (2005). doi:10.1021/pr049831k

[39] Bose, A., Pritchett, M.A., Rother, M., Metcalf, W.W.: Differential Regulation of the Three Methanol Methyltransferase Isozymes in *Methanosarcina acetivorans* C2A. J. Bacteriol. **188**(20), 7274–7283 (2006). doi:10.1128/JB.00535-06. http://jb.asm.org/content/188/20/7274.full.pdf+html

[40] Li, L., Li, Q., Rohlin, L., Kim, U., Salmon, K., Rejtar, T., Gunsalus, R.P., Karger, B.L., Ferry, J.G.: Quantitative Proteomic and Microarray Analysis of the Archaeon *Methanosarcina acetivorans* Grown with Acetate versus Methanol. J. Proteome Res. **6**(2), 759–771 (2007). doi:10.1021/pr060383l

[41] Bose, A., Metcalf, W.W.: Distinct regulators control the expression of methanol methyltransferase isozymes in *Methanosarcina acetivorans* C2A. Mol. Microbiol. **67**(3), 649–661 (2008). doi:10.1111/j.1365-2958.2007.06075.x

[42] Anderson, K.L., Apolinario, E.E., MacAuley, S.R., Sowers, K.R.: A 5′ Leader Sequence Regulates Expression of Methanosarcinal CO Dehydrogenase/Acetyl Coenzyme A Synthase. J. Bacteriol. **191**(22), 7123–7128 (2009). doi:10.1128/JB.00731-09. http://jb.asm.org/content/191/22/7123.full.pdf+html

[43] Buan, N.R., Metcalf, W.W.: Methanogenesis by *Methanosarcina acetivorans* involves two structurally and functionally distinct classes of heterodisulfide reductase. Mol. Microbiol. **75**(4), 843–853 (2010). doi:10.1111/j.1365-2958.2009.06990.x

[44] Price, M.N., Huang, K.H., Alm, E.J., Arkin, A.P.: A novel method for accurate operon predictions in all sequenced prokaryotes. Nucleic Acids Res. **33**(3), 880–892 (2005). doi:10.1093/nar/gki232

[45] Taboada, B., Ciria, R., Martinez-Guerrero, C.E., Merino, E.: ProOpDB: Prokaryotic Operon DataBase. Nucleic Acids Res. **40**(D1), 627–631 (2011). doi:10.1093/nar/gkr1020

[46] McClure, R., Balasubramanian, D., Sun, Y., Bobrovskyy, M., Sumby, P., Genco, C.A., Vanderpool, C.K., Tjaden, B.: Computational analysis of bacterial RNA-Seq data. Nucleic Acids Res. **41**(14), 140 (2013)

[47] Mao, X., Ma, Q., Zhou, C., Chen, X., Zhang, H., Yang, J., Mao, F., Lai, W., Xu, Y.: DOOR 2.0: presenting operons and their functions through dynamic and integrated views. Nucleic Acids Res. **42**(D1), 654–659 (2013). doi:10.1093/nar/gkt1048

[48] Benedict, M.N., Gonnerman, M.C., Metcalf, W.W., Price, N.D.: Genome-Scale Metabolic Reconstruction and Hypothesis Testing in the Methanogenic Archaeon *Methanosarcina acetivorans* C2A. J. Bacteriol. **194**(4), 855–865 (2012). doi:10.1128/jb.06040-11

[49] Kumar, V.S., Ferry, J.G., Maranas, C.D.: Metabolic reconstruction of the archaeon methanogen methanosarcina acetivorans. BMC Systems Biology **5**(1), 28 (2011). doi:10.1186/1752-0509-5-28

[50] O'Donoghue, P., Sethi, A., Woese, C.R., Luthey-Schulten, Z.A.: The evolutionary history of Cys-tRNA^{Cys} formation. Proc. Natl. Acad. Sci. U.S.A. **102**(52), 19003–19008 (2005). doi:10.1073/pnas.0509617102

[51] Sauerwald, A., Zhu, W., Major, T.A., Roy, H., Palioura, S., Jahn, D., Whitman, W.B., 3rd, J.R.Y., Ibba, M., Söll, D.: RNA-Dependent Cysteine Biosynthesis in Archaea. Science **307**(5717), 1969–1972 (2005). doi:10.1126/science.1108329

[52] Miller, D., Wang, Y., Xu, H., Harich, K., White, R.H.: Biosynthesis of the 5-(Aminomethyl)-3-furanmethanol Moiety of Methanofuran. Biochem. **53**(28), 4635–4647 (2014). doi:10.1021/bi500615p

[53] Wang, Y., Xu, H., Harich, K.C., White, R.H.: Identification and Characterization of a Tyramine–Glutamate Ligase (MfnD) Involved in Methanofuran Biosynthesis. Biochem. **53**(39), 6220–6230 (2014). doi:10.1021/bi500879h

[54] Wang, Y., Jones, M.K., Xu, H., Ray, W.K., White, R.H.: Mechanism of the Enzymatic Synthesis of 4-(Hydroxymethyl)-2-furancarboxaldehyde-phosphate (4-HFC-P) from Glyceraldehyde-3-phosphate Catalyzed by 4-HFC-P Synthase. Biochem. **54**(19), 2997–3008 (2015). doi:10.1021/acs.biochem.5b00176

[55] Wang, Y., Xu, H., White, R.H.: Identification of the Final Two Genes Functioning in Methanofuran Biosynthesis in *Methanocaldococcus jannaschii.* Journal of Bacteriology **197**(17), 2850–2858 (2015). doi:10.1128/JB.00401-15

[56] Matschiavelli, N., Rother, M.: Role of a putative tungsten-dependent formylmethanofuran dehydrogenase in *Methanosarcina acetivorans.* Arch Microbiol **197**(3), 379–388 (2014). doi:10.1007/s00203-014-1070-3

[57] Fu, H., Metcalf, W.W.: Genetic Basis for Metabolism of Methylated Sulfur Compounds in Methanosarcina Species. J. Bacteriol. **197**(8), 1515–1524 (2015). doi:10.1128/jb.02605-14

[58] Isobe, K., Ogawa, T., Hirose, K., Yokoi, T., Yoshimura, T., Hemmi, H.: Geranylgeranyl reductase and ferredoxin from *methanosarcina acetivorans* are required for the synthesis of fully reduced archaeal membrane lipid in escherichia coli cells. Journal of Bacteriology **196**(2), 417–423 (2013). doi:10.1128/jb.00927-13

[59] Mori, T., Isobe, K., Ogawa, T., Yoshimura, T., Hemmi, H.: A phytoene desaturase homolog gene from the methanogenic archaeon *methanosarcina acetivorans* is responsible for hydroxyarchaeol biosynthesis. Biochemical and Biophysical Research Communications **466**(2), 186–191 (2015). doi:10.1016/j.bbrc.2015.09.001

[60] Ogawa, T., Emi, K.-i., Koga, K., Yoshimura, T., Hemmi, H.: A cis-prenyltransferase from *methanosarcina acetivorans* catalyzes both head-to-tail and nonhead-to-tail prenyl condensation. FEBS J **283**(12), 2369–2383 (2016). doi:10.1111/febs.13749

[61] Sowers, K.R., Gunsalus, R.P.: Halotolerance in Methanosarcina spp: Role of N-Acetyl-beta-Lysine, alpha-Glutamate, Glycine Betaine, and K+ as compatible solutes for Osmotic Adaptation. Appl. Environ. Microbiol. **61**(12), 4382–4388 (1995)

[62] Santiago-Martínez, M.G., Encalada, R., Lira-Silva, E., Pineda, E., Gallardo-Pérez, J.C., Reyes-García, M.A., Saavedra, E., Moreno-Sánchez, R., Marín-Hernández, A., Jasso-Chávez, R.: The nutritional status of *methanosarcina acetivorans* regulates glycogen metabolism and gluconeogenesis and glycolysis fluxes. FEBS J **283**(10), 1979–1999 (2016). doi:10.1111/febs.13717

[63] Wolfe, R.S.: Chapter one - techniques for cultivating methanogens. In: Rosenzweig, A.C., Ragsdale, S.W. (eds.) Methods in Methane Metabolism, Part A. Methods in Enzymology, vol. 494, pp. 1–22. Academic Press, ??? (2011). doi:10.1016/B978-0-12-385112-3.00001-9

[64] Smoot, M.E., Ono, K., Ruscheinski, J., Wang, P.-L., Ideker, T.: Cytoscape 2.8: new features for data integration and network visualization. Bioinformatics **27**(3), 431–432 (2010). doi:10.1093/bioinformatics/btq675

[65] King, Z.A., Dräger, A., Ebrahim, A., Sonnenschein, N., Lewis, N.E., Palsson, B.Ø.: Escher: A Web Application for Building, Sharing, and Embedding Data-Rich Visualizations of Biological Pathways. PLOS Comput. Biol. **11**(8), 1004321 (2015). doi:10.1371/journal.pcbi.1004321

[66] Redon, E., Loubiere, P., Cocaign-Bousquet, M.: Role of mRNA stability during genome-wide adaptation of *Lactococcus lactis* to carbon starvation. J. Biol. Chem. **280**(43), 36380–36385 (2005)

[67] Cao, Y., Li, J., Jiang, N., Dong, X.: Mechanism for Stabilizing mRNAs Involved in Methanol-Dependent Methanogenesis of Cold-Adaptive *Methanosarcina mazei* zm-15. Appl. Environ. Microbiol. **80**(4), 1291–1298 (2013). doi:10.1128/aem.03495-13

[68] Kratzer, C., Carini, P., Hovey, R., Deppenmeier, U.: Transcriptional Profiling of Methyltransferase Genes during Growth of *Methanosarcina mazei* on Trimethylamine. J. Bacteriol. **191**(16), 5108–5115 (2009). doi:10.1128/jb.00420-09

[69] Youngblut, N.D., Wirth, J.S., Henriksen, J.R., Smith, M., Simon, H., Metcalf, W.W., Whitaker, R.J.: Genomic and phenotypic differentiation among *Methanosarcina mazei* populations from Columbia River sediment. ISME J. **9**(10), 2191–2205 (2015). doi:10.1038/ismej.2015.31

[70] Boone, D.R., Mathrani, I.M., Liu, Y., Menaia, J.A.G.F., Mah, R.A., Boone, J.E.: Isolation and Characterization of *Methanohalophilus portucalensis* sp. nov. and DNA Reassociation Study of the Genus Methanohalophilus. Int. J. Syst. Bacteriol. **43**(3), 430–437 (1993). doi:10.1099/00207713-43-3-430

[71] Wilson, D., Charoensawan, V., Kummerfeld, S.K., Teichmann, S.A.: DBD taxonomically broad transcription factor predictions: new content and functionality. Nucleic Acids Res. **36**(suppl_1), 88–92 (2008). doi:10.1093/nar/gkm964. http://nar.oxfordjournals.org/cgi/reprint/36/suppl_1/D88.pdf

[72] Bose, A., Kulkarni, G., Metcalf, W.W.: Regulation of putative methyl-sulphide methyltransferases in *Methanosarcina acetivorans* C2A. Mol. Microbiol. **74**(1), 227–238 (2009). doi:10.1111/j.1365-2958.2009.06864.x

[73] Reichlen, M.J., Vepachedu, V.R., Murakami, K.S., Ferry, J.G.: MreA Functions in the Global Regulation of Methanogenic Pathways in *Methanosarcina acetivorans*. mBio **3**(4), 00189–120018912 (2012). doi:10.1128/mbio.00189-12

[74] Catlett, J.L., Ortiz, A.M., Buan, N.R.: Rerouting Cellular Electron Flux To Increase the Rate of Biological Methane Production. Appl. Environ. Microbiol. **81**(19), 6528–6537 (2015). doi:10.1128/aem.01162-15

[75] Yoon, S.H., Turkarslan, S., Reiss, D.J., Pan, M., Burn, J.A., Costa, K.C., Lie, T.J., Slagel, J., Moritz, R.L., Hackett, M., Leigh, J.A., Baliga, N.S.: A systems level predictive model for global gene regulation of methanogenesis in a hydrogenotrophic methanogen. Genome Res. **23**(11), 1839–1851 (2013). doi:10.1101/gr.153916.112

[76] Reichlen, M.J., Murakami, K.S., Ferry, J.G.: Functional Analysis of the Three TATA Binding Protein Homologs in *Methanosarcina acetivorans*. J. Bacteriol. **192**(6), 1511–1517 (2010). doi:10.1128/jb.01165-09

[77] Neidhardt, F.C., III, R.C., Ingraham, J.L., Lin, E.C.C., Low, K.B., Magasanik, B., Reznikoff, W.S., Riley, M., Schaechter, M., Umbarger, H.E. (eds.): Escherichia Coli and Salmonella: Cellular and Molecular Biology, 2nd edn. ASM Press, Washington, D.C. (1996)

[78] Zaigler, A., Schuster, S.C., Soppa, J.: Construction and usage of a onefold-coverage shotgun DNA microarray to characterize the metabolism of the archaeon haloferax volcanii. Molecular Microbiology **48**(4), 1089–1105 (2003). doi:10.1046/j.1365-2958.2003.03497.x

[79] Sonenberg, N., Hinnebusch, A.G.: Regulation of translation initiation in eukaryotes: Mechanisms and biological targets. Cell **136**(4), 731–745 (2009). doi:10.1016/j.cell.2009.01.042

[80] Lange, C., Zaigler, A., Hammelmann, M., Twellmeyer, J., Raddatz, G., Schuster, S.C., Oesterhelt, D., Soppa, J.: Genome-wide analysis of growth phase-dependent translational and transcriptional regulation in halophilic archaea. BMC Genomics **8**(1), 1–16 (2007). doi:10.1186/1471-2164-8-415

[81] Brenneis, M., Soppa, J.: Regulation of translation in haloarchaea: 5'- and 3'-utrs are essential and have to functionally interact *In Vivo*. PLoS ONE **4**(2), 1–8 (2009). doi:10.1371/journal.pone.0004484

[82] Jasso-Chávez, R., Santiago-Martínez, M.G., Lira-Silva, E., Pineda, E., Zepeda-Rodríguez, A., Belmont-Díaz, J., Encalada, R., Saavedra, E., Moreno-Sánchez, R.: Air-Adapted *Methanosarcina acetivorans* Shows High Methane Production and Develops Resistance against Oxygen Stress. PLOS ONE **10**(2), 0117331 (2015). doi:10.1371/journal.pone.0117331

[83] Sowers, K.R., Boone, J.E., Gunsalus, R.P.: Disaggregation of Methanosarcina spp. and Growth as Single Cells at Elevated Osmolarity. Appl. Environ. Microbiol. **59**(11), 3832–3839 (1993)

[84] Metcalf, W.W., Zhang, J.K., Shi, X., Wolfe, R.S.: Molecular, genetic, and biochemical characterization of the serC gene of *Methanosarcina barkeri* Fusaro. J. Bacteriol. **178**(19), 5797–5802 (1996)

[85] Peterson, J.R., Labhsetwar, P., Ellermeier, J.R., Kohler, P.R.A., Jain, A., Ha, T., Metcalf, W.W., Luthey-Schulten, Z.: Towards a Computational Model of a Methane Producing Archaeum. Archaea **2014**, 1–18 (2014). doi:10.1155/2014/898453

[86] Stewart, F.K., Ottesen, E.A., DeLong, E.F.: Development and quantitative analyses of a universal rRNA-subtraction protocol for microbial metatranscriptomics. ISME J. **4**(7), 896–907 (2010). doi:10.1038/ismej.2010.18

[87] Benson, D.A., Cavanaugh, M., Clark, K., Karsch-Mizrachi, I., Lipman, D.J., Ostell, J., Sayers, E.W.: GenBank. Nucleic Acids Res. **41**(D1), 36–42 (2012). doi:10.1093/nar/gks1195

[88] Millman, K.J., Aivazis, M.: Python for Scientists and Engineers. Comput. in Sci. & Eng. **13**(2), 9–12 (2011). doi:10.1109/mcse.2011.36

[89] Lorenz, R., Bernhart, S.H., zu Siederdissen, C.H., Tafer, H., Flamm, C., Stadler, P.F., Hofacker, I.L.: ViennaRNA Package 2.0. Algorithms Mol. Biol. **6**(1), 26 (2011). doi:10.1186/1748-7188-6-26

[90] Mathews, D.H., Disney, M.D., Childs, J.L., Schroeder, S.J., Zuker, M., Turner, D.H.: Incorporating chemical modification constraints into a dynamic programming algorithm for prediction of RNA secondary structure. Proc. Natl. Acad. Sci. U.S.A. **101**(19), 7287–7292 (2004). doi:10.1073/pnas.0401799101

[91] Andronescu, M., Condon, A., Hoos, H.H., Mathews, D.H., Murphy, K.P.: Efficient parameter estimation for RNA secondary structure prediction. Bioinformatics **23**(13), 19–28 (2007). doi:10.1093/bioinformatics/btm223

[92] Benedict, M.N., Henriksen, J.R., Metcalf, W.W., Whitaker, R.J., Price, N.D.: ITEP: An integrated toolkit for exploration of microbial pan-genomes. BMC Genomics **15**(1), 8 (2014). doi:10.1186/1471-2164-15-8

[93] Huerta-Cepas, J., Serra, F., Bork, P.: ETE 3: Reconstruction, analysis, and visualization of phylogenomic data. Mol Biol Evol **33**(6), 1635–1638 (2016). doi:10.1093/molbev/msw046

[94] Blazier, A.S., Papin, J.A.: Integration of expression data in genome-scale metabolic network reconstructions. Front. Physiol. **3** (2012). doi:10.3389/fphys.2012.00299

[95] Guss, A.M., Mukhopadhyay, B., Zhang, J.K., Metcalf, W.W.: Genetic analysis of mch mutants in two Methanosarcina species demonstrates multiple roles for the methanopterin-dependent C-1 oxidation/reduction pathway and differences in H(2) metabolism between closely related species. Mol. Microbiol. **55**(6), 1671–1680 (2005)

[96] Opulencia, R.B., Bose, A., Metcalf, W.W.: Physiology and Posttranscriptional Regulation of Methanol:Coenzyme M Methyltransferase Isozymes in *Methanosarcina acetivorans* C2A. J. Bacteriol. **191**(22), 6928–6935 (2009). doi:10.1128/jb.00947-09

[97] Sowers, K.R., Baron, S.F., Ferry, J.G.: *Methanosarcina acetivorans* sp. nov., an Acetotrophic Methane-Producing Bacterium Isolated from Marine Sediments. Appl. Environ. Microbiol. **47**(5), 971–978 (1984)

[98] Schuetz, R., Zamboni, N., Zampieri, M., Heinemann, M., Sauer, U.: Multidimensional Optimality of Microbial Metabolism. Science **336**(6081), 601–604 (2012). doi:10.1126/science.1216882

[99] Seyednasrollah, F., Laiho, A., Elo, L.L.: Comparison of software packages for detecting differential expression in RNA-seq studies. Brief. Bioinform. **16**(1), 59–70 (2013). doi:10.1093/bib/bbt086

[100] Rapaport, F., Khanin, R., Liang, Y., Pirun, M., Krek, A., Zumbo, P., Mason, C.E., Socci, N.D., Betel, D.: Comprehensive evaluation of differential gene expression analysis methods for RNA-seq data. Genome Biol. **14**(9), 95 (2013). doi:10.1186/gb-2013-14-9-r95

[101] Sundararaj, S.: The CyberCell database (CCDB): a comprehensive, self-updating, relational database to coordinate and facilitate in silico modeling of *escherichia coli*. Nucleic Acids Res. **32**(90001), 293–295 (2004). doi:10.1093/nar/gkh108

[102] Jain, A., Liu, R., Ramani, B., Arauz, E., Ishitsuka, Y., Ragunathan, K., Park, J., Chen, J., Xiang, Y.K., Ha, T.: Probing cellular protein complexes using single-molecule pull-down. Nature **473**(7348), 484–488 (2011). doi:10.1038/nature10016

[103] Ebrahim, A., Lerman, J.A., Palsson, B.Ø., Hyduke, D.R.: COBRApy: COnstraints-Based Reconstruction and Analysis for Python. BMC Sys. Biol. **7**(74) (2013). doi:10.1186/1752-0509-7-74

[104] Liu, Y., Beer, L.L., Whitman, W.B.: Methanogens: a window into ancient sulfur metabolism. Trends Microbiol. **20**(5), 251–258 (2012). doi:0.1016/j.tim.2012.02.002

[105] Hao, B., Gong, W., Ferguson, T.K., James, C.M., Krzycki, J.A., Chan, M.K.: A New UAG-Encoded Residue in the Structure of a Methanogen Methyltransferase. Science **296**(5572), 1462–1466 (2002). doi:10.1126/science.1069556

[106] Mahapatra, A., Patel, A., Soares, J.A., Larue, R.C., Zhang, J.K., Metcalf, W.W., Krzycki, J.A.: Characterization of a *Methanosarcina acetivorans* mutant unable to translate UAG as pyrrolysine. Mol. Microbiol. **59**(1), 56–66 (2006). doi:10.1111/j.1365-2958.2005.04927.x

[107] Gaston, M.A., Zhang, L., Green-Church, K.B., Krzycki, J.A.: The complete biosynthesis of the genetically encoded amino acid pyrrolysine. Nature **471**, 647–650 (2011). doi:10.1038/nature09918

[108] Heinie-Dobbernack, E., Schoberth, S.M., Sahm, H.: Relationship of Intracellular Coenzyme F420 Content to Growth and Metabolic Activity of *Methanobacterium bryantii* and *Methanosarcina barkeri*. Appl. Environ. Microbiol. **54**(2), 454–459 (1988)

[109] Baresi, L., Wolfe, R.S.: Levels of coenzyme F420, coenzyme M, hydrogenase, and methylcoenzyme M methylreductase in acetate-grown Methanosarcina. Appl. Environ. Microbiol. **41**(2), 388–391 (1981)

[110] Gorris, L.G.M., van der Drift, C.: Methanogenic cofactors in pure cultures of methanogens in relation to substrate utilization. In: Dubourguier, H.C., Albagnac, G., Montreuil, J., Romond, C., Sautiere, P., Guil-

laume, J. (eds.) Biology of Anaerobic Bacteria, pp. 144–150. Elsevier Science Publishing, Inc., Amsterdam (1988)

[111] Peck, M.W.: Changes in concentrations of coenzyme F420 analogs during batch growth of *Methanosarcina barkeri* and *Methanosarcina mazei*. Appl. Environ. Microbiol. **55**(4), 940–945 (1989)

[112] Edgeworth, F.Y.: XXII. on a new method of reducing observations relating to several quantities. Philosophical Magazine Series 5 **25**(154), 184–191 (1888). doi:10.1080/14786448808628170

[113] Pritchett, M.A., Metcalf, W.W.: Genetic, physiologic and biochemical characterization of multiple methanol methyltransferase isozymes in *methanosarcina acetiverans* c2a. Mol. Microbiol. **56**(5), 1183–1194 (2005). doi:10.1111/j.1365-2958.2005.04616.x

[114] Oelgeschlager, E., Rother, M.: *In vivo* role of three fused corrinoid/methyl transfer proteins in *methanosarcina acetivorans*. Mol. Microbiol. **72**(5), 1260–1272 (2009). doi:10.1111/j.1365-2958.2009.06723.x

[115] Bose, A., Pritchett, M.A., Metcalf, W.W.: Genetic analysis of the methanol- and methylamine-specific methyltransferase 2 genes of *methanosarcina acetivorans* c2a. J. Bacteriol. **190**(11), 4017–4026 (2008). doi:10.1128/JB.00117-08

[116] Sowers, K.R., Nelson, M.J., Ferry, J.G.: Growth of acetotrophic, methane-producing bacteria in a ph auxostat. Curr. Microbiol. **11**, 227–229 (1984)

[117] Summer, H.: Improved aproach for transferring and cultivating *methanosarcina acetivorans*. Lett. Appl. Microbiol. **48**(6), 786–789 (2009). doi:10.1111/j.1472-765X.2009.02592.x

[118] Oelgeschlager, E., Rother, M.: Influence of carbon monoxide on metabolite formation in *methanosarcina acetivorans*. FEMS Microbiol. Lett. **292**(2), 254–260 (2009). doi:10.1111/j.1574-6968.2009.01492.x

[119] Lessner, D.J., Lhu, L., Wahal, C.S., Ferry, J.G.: An engineered methanogenic pathway derived from the domains *bacteria* and *archaea*. MBio **1**(5), 1–4 (2010). doi:10.1128/mBio.00243-10
